# Interventions to increase youth employment: An evidence and gap map

**DOI:** 10.1002/cl2.1216

**Published:** 2022-02-15

**Authors:** Robert Apunyo, Howard White, Caroline Otike, Thomas Katairo, Susana Puerto, Drew Gardiner, Alison Annet Kinengyere, John Eyers, Ashrita Saran, Ekwaro A. Obuku

**Affiliations:** ^1^ Africa Centre for Systematic Reviews and Knowledge Translation, Makerere University College of Health Sciences Makerere University College of Health Sciences Kampala Uganda; ^2^ Campbell Collaboration New Delhi India; ^3^ International Labour Organization Geneva Switzerland; ^4^ Sir Albert Cook Medical Library Makerere University College of Health Sciences Kampala Uganda; ^5^ International Initiative for Impact Evaluation London UK

## Abstract

**Background:**

Globally, 13% of the youth are not in education, employment or training (NEET). Moreover, this persistent problem has been exacerbated by the shock of Covid‐19 pandemic. More youth from disadvantaged backgrounds are likely unemployed than those from better off backgrounds. Thus, the need for increased use of evidence in the design and implementation of youth employment interventions to increase effectiveness and sustainability of interventions and outcomes. Evidence and gap maps (EGMs) can promote evidence‐based decision making by guiding policy makers, development partners and researchers to areas with good bodies of evidence and those with little or no evidence. The scope of the Youth Employment EGM is global. The map covers all youth aged 15–35 years. The three broad intervention categories included in the EGM are: strengthening training and education systems, enhancing labour market and, transforming financial sector markets. There are five outcome categories: education and skills; entrepreneurship; employment; welfare and economic outcomes. The EGM contains impact evaluations of interventions implemented to increase youth employment and systematic reviews of such single studies, published or made available between 2000 and 2019.

**Objectives:**

The primary objective was to catalogue impact evaluations and systematic reviews on youth employment interventions to improve discoverability of evidence by decision makers, development patterners and researchers, so as to promote evidence‐based decision making in programming and implementation of youth employment initiatives.

**Search Methods:**

Twenty databases and websites were searched using a validated search strategy. Additional searches included searching within 21 systematic reviews, snowballing 20 most recent studies and citation tracking of 10 most recent studies included in the EGM.

**Selection Criteria:**

The study selection criteria followed the PICOS approach of population, intervention, relevant comparison groups, outcomes and study design. Additional criterion is; study publication or availability period of between 2000 and 2021. Only impact evaluations and systematic reviews that included impact evaluations were selected.

**Data Collection and Analysis:**

A total of 14,511 studies were uploaded in EPPI Reviewer 4 software, upon which 399 were selected using the criteria provided above. Coding of data took place in EPPI Reviewer basing on predefined codes. The unit of analysis for the report is individual studies where every entry represents a combination of interventions and outcomes.

**Main Results:**

Overall, 399 studies (21 systematic reviews and 378 impact evaluations) are included in the EGM. Impact evaluations (*n* = 378) are much more than the systematic reviews (*n* = 21). Most impact evaluations are experimental studies (*n *= 177), followed by non‐experimental matching (*n* = 167) and other regression designs (*n* = 35). Experimental studies were mostly conducted in both Lower‐income countries and Lower Middle Income countries while non‐experimental study designs are the most common in both High Income and Upper Middle Income countries. Most evidence is from low quality impact evaluations (71.2%) while majority of systematic reviews (71.4% of 21) are of medium and high quality rating. The area saturated with most evidence is the intervention category of ‘training’, while the underrepresented are three main intervention sub‐categories: information services; decent work policies and; entrepreneurship promotion and financing. Older youth, youth in fragility, conflict and violence contexts, or humanitarian settings, or ethnic minorities or those with criminal backgrounds are least studied.

**Conclusions:**

The Youth Employment EGM identifies trends in evidence notably the following:

- Most evidence is from high‐income countries, an indication of the relationship between a country's income status and research productivity.
- The most common study designs are experimental.
- Most of the evidence is of low quality. This finding serves to alert researchers, practitioners and policy makers that more rigorous work is needed to inform youth employment interventions.

Blending of interventions is practiced. While this could be an indication that blended intervention could be offering better outcomes, this remains an area with a research gap.

## 1 PLAIN LANGUAGE SUMMARY

### 1.1 The evidence for youth employment interventions is unevenly distributed by geography and population sub‐groups, and much of it is of poor quality

There is considerable evidence on training‐based youth employment interventions across all outcomes included in the evidence and gap map (EGM), but scarce evidence in the ‘information services’ and ‘decent work policies’ categories of interventions. Much of the evidence is of low quality.

#### 1.1.1 What is this EGM about?

Globally, approximately 13% of youth are ‘not in education, employment or training’ (NEET). This situation is aggravated by the shock of the Covid‐19 pandemic. This EGM shows the available evidence on youth employment from systematic reviews and impact evaluations.

Decisionmakers and implementers across all countries should use context‐specific evidence to increase effectiveness and sustainability of youth employment interventions and outcomes.

**What is the aim of this EGM?**

The aim of this EGM is to catalogue all the available evidence from impact evaluations and systematic reviews to increase youth employment across all countries.

#### 1.1.2 What studies are included?

The EGM includes systematic reviews and impact evaluations that assess effectiveness of youth employment interventions. The studies report interventions for young people aged 15‐35 years. They also report intended or unintended or adverse outcomes. The EGM contains 399 studies: 21 systematic reviews and 378 impact evaluations. The included impact evaluations are predominantly experimental studies.

#### 1.1.3 What are the main findings of this evidence and gap map?

There is uneven distribution of studies across intervention categories. The most frequent intervention category is ‘training’, reported by 283 out of 399 studies. It is followed by the ‘support to employment’ intervention domain with 182 out of 399 studies. There are relatively few studies for ‘information services’, ‘entrepreneurship promotion’ and ‘financing interventions’.

The most dominant outcomes are related to employment, such as ‘earnings & salary’ and ‘employment status & duration’, reported in 345 of the 399 studies. This is followed by ‘welfare’ (121) and ‘education and skills’ (97). There are few studies for ‘entrepreneurship’ outcomes.

At least 37% of the studies in the EGM combined multiple categories of interventions. The ‘training’ and the ‘support to employment’ intervention categories are most evident in different intervention combinations.

There is a general lack of high quality evidence given that the majority (73.4%) of impact evaluations have a low confidence quality rating. Three major flaws in impact evaluation reports are that:

1. many young people drop out of the interventions or authors fail to report that information
2. implementers fail to take into account other factors with the potential to affect interventions and outcomes
3. most studies have many variations in the characteristics of youth, like education level and age as a baseline or at the beginning of the interventions.

#### 1.1.4 What do the findings of the map mean?

Mastercard Foundation and the Youth Futures Foundation plan to use this EGM to promote innovation and knowledge‐sharing, and to inform their funding decisions on programmes and systematic reviews.

While the evidence base is relatively large, it is weak when it comes to disadvantaged populations of youth as well as interventions under ‘decent work policies’, which include labour standards and accountability systems, and ‘information services’, such as value chain development and labour market information.

The quality of systematic reviews and impact evaluations requires improvement. More studies from low‐income countries are needed, especially on disadvantaged youth.

#### 1.1.5 How up‐to‐date is this EGM?

The authors searched for studies published up to January 2020.

## 2 BACKGROUND

Globally, the population of young people is estimated at 1.2 billion, which is 16% of the world's population (United Nations, 2018). Worldwide, approximately 13% of young men and 30% of young women were classified as not in education, employment or training (NEET) in 2018. Youth who are NEET are almost excluded from the labour market because they are not gaining any skills to prepare them for future employment. Moreover, in the long run, a high NEET rate undermines the growth of a national economy over a sustained period (International Labour Organization [ILO], 2019a).

In 2017, the global rate of youth unemployment was estimated at 13%, though Sub‐Saharan Africa and, Latin America and the Caribbean had the most disturbing situations. While youth unemployment in Sub‐Saharan Africa was 11.7%, some countries experienced extraordinarily high rates of youth unemployment. For instance, in South Africa youth unemployment rate was about 57.4%, and the highest in the region. In Lesotho, Mozambique and Namibia youth unemployment rates were estimated at 38.5%, 42.7% and 45.5%, respectively. Similarly, in Latin America and the Caribbean, Argentina, and Brazil registered highest youth unemployment rates of 24.7% and 30.5% respectively, pushing the regional average to 18.9%, in 2017 (United Nations, 2018).

Regions of the world often have some unique causes of youth unemployment. For instance, young women are generally discriminately unemployed than young men, with the situation exceedingly high in Middle East. However, this phenomenon is flipped in Western Europe and Eastern Asia where unemployment is higher among young men than young women. In the case of Eastern Asia, the situation is driven largely by China's inclusion of young women in its expanding manufacturing sector (ILO, 2019a). In Middle East and North Africa, the higher unemployment of young women than men can be attributed to the conservative social norms that may determine opportunities considered appropriate for women. So, women may mainly seek out opportunities in the public sector and avoid jobs in industries dominated by men (ILO, 2019b). In Sub‐Saharan Africa, one of the major causes of youth unemployment stems from the weak education systems leading to huge skills mismatch between skills provided by the education systems and labour market needs. In addition, Sub‐Saharan Africa, has experienced a bulging young population despite the small sizes of economies (The African Capacity Building Foundation, 2017).

Moreover, most of the world and particularly Africa is experiencing high growth in youth population, where the situation is envisaged to increase the continent's labour force to 375 million by 2030. The implication is that by 2035, there will be more young people in Africa available for the labour market each year than in the rest of the world combined (Mastercard Foundation, 2019). At a macro level, some drivers to youth unemployment include huge increases in labour supply, low aggregate demand for labour and, a mismatch between economic growth path and skills requirements majorly as a result of training deficits (De Lannoy et al., 2018). Unfortunately, the predicament of youth has been exacerbated by the shock of Covid‐19 pandemic, which is worsening employment, livelihoods and poverty around the world.

Employment and economic empowerment are essential components of a strong foundation for all youth regardless of their gender identity and disadvantaged status. So, having decent work is crucial for young people and their future but it also has multiplier effects on local communities and the world as a whole (United Nations, 2018). Decent work refers to a composition of the aspirations of people in their working lives. It involves opportunities for work that are productive and deliver fair income, security in the workplace and social protection for families, better prospects for personal development and social integration (International Labour Organization, 2019a).

Addressing youth unemployment requires investment in job creation initiatives for the ever‐increasing population and tackling the skills mismatch problem contributing to the low school‐to‐work transition situation. Clearly, efforts to stimulate youth employment require diversification of employment sector through investment in education, skills training, bolstering small and medium‐sized enterprises and, microcredit provision (United Nations, 2018).

The UNDP Sustainable Development Goals (SDG) 8 and SDG 10 seek to reduce youth unemployment and inequality of all forms respectively (United Nations, 2019a). The SDG 8 sets three targets for youth employment. Target 8.5, expects countries, by 2030 to achieve full and productive employment and decent work for all women and men, including for young people and persons with disabilities, and equal pay for work of equal value. Target 8.6, expects countries, by 2020 to substantially reduce the proportion of youth not in employment, education or training and, target 8.b, by 2020, develop and operationalise a global strategy for youth employment and implement the Global Jobs Pact of the ILO (United Nations, 2019b). The SDG 10, ‘Reduce Inequality within and among countries’ in Target 10.2, expects countries by 2030, to achieve empowerment and promotion of social, economic and political inclusion of all people irrespective of age, sex disability, race, ethnicity and economic status (United Nations, 2019c).

Consequently, global, regional and country‐based initiatives have been put in place to deal with youth unemployment. For instance, the United Nations Youth Strategy has been developed with several priorities of which the third talks about prompting economic empowerment through decent work, by supporting young people's greater access to decent work and productive employment (United Nations, n.d.). Similarly, Mastercard Foundation through its ‘Young Africa Works Strategy’ has set out an ambitious goal to enable 30 million youth in Africa to find jobs by 2030 partly through the promotion of sharing evidence‐based knowledge and innovation with stakeholders; supporting use of technology to drive impact and scale and; empowering young women (Mastercard Foundation, 2019).

To respond to the above, there is an increased need to invest in making available evidence on youth employment interventions discoverable by decision makers, development partners, researchers and other stakeholders. Evidence and gap maps (EGMs) can contribute to achieving this by identifying areas in which there are good bodies of synthesised knowledge to inform policy, and those areas in which there is little or no evidence to guide commissioning of coordinated research programmes.

### 2.1 Intervention: All interventions that increase youth employment

All interventions or programmes or projects aimed at helping youth to find and sustain employment are the focus of this EGM. This EGM broadly categories the interventions into three domains: strengthening training and education systems, enhancing labour market and, transforming financial sector markets.

Strengthening training and education systems, includes the conventional education systems and various forms of training such as direct training often provided to the unemployed. The other types of training namely; up‐skilling and retraining/re‐skilling normally target youth who are already in employment, to ensure that their skill relevant to the changing demands of employers.

Enhancing labour markets category of interventions includes: decent work policies, support to employment services, and Information services. Decent work polices generally regulate the relationship between employers and employees in the employment environment through application of labour standards, regulations and accountability systems. Support to employment interventions are generally meant to help youth find jobs through provision of jobs via initiatives like programmes for overseas employment and public works programmes.

Transforming financial sector markets based interventions focus on entrepreneurship promotion and financing. This category of interventions tend to be popular for targeting disadvantaged youth especially those with low levels of education. As Datta et al. (2018) observe, labour market opportunities are significantly influenced by the reverence of skills for the existing job market. An intervention like self‐help or financing groups tends to be dominant among youth with less education and training. Self‐help groups are small groups that save a certain amount of money on weekly or monthly basis and issue loans to members out of their collective savings (Flynn, 2013).

The focus of this EGM is therefore broad, for example covering economic and welfare dimensions. So, outcomes of the various interventions go beyond the youth getting employment which is often the primary motivation of implementing youth employment programmes.

### 2.2 Scope of the youth employment EGM^1^

Geographically, this EGM is global in coverage, considering all countries regardless of their level of development. That means all world geographical regions and the World Bank country classification by income have been covered. The geographical regions are: Sub‐Saharan Africa, Latin America and Caribbean, East Asia and Pacific, Middle East and North Africa, South Asia, Europe and Central Asia, and North America. The World Bank country classification by income includes: low‐income countries, lower‐middle income, upper middle income, and high‐income countries (World Bank, 2020).

By population, the map covered all young women and men aged 15–35 years from all countries. The three broad intervention categories include in the EGM are: strengthening training and education systems, enhancing labour market and, transforming financial sector markets.

The outcomes fall into five categories: education and skills, entrepreneurship, employment, welfare and economic.

In terms of evidence, the map included impact evaluations of interventions aimed at increasing youth employment and systematic reviews of such single studies, published or made available between January 2000 and December 2019.

### 2.3 Why it is important to develop the EGM

EGMs guide policy makers, development partners and researchers to available evidence to inform programme design and implementation of development interventions. Decision‐makers and researchers often lack awareness about the extent of evidence base, so can maps help in increasing the discoverability, and thus use of that evidence for evidence‐informed decision making in international development policy and practice. The immediate potential application of the youth employment EGM is its contribution to the implementation of the Mastercard Foundation's strategy titled, ‘Africa Works—Mastercard Foundation Strategy 2018–2030’. The goal of the strategy is to enable 30 million youth in Africa to secure dignified and fulfilling employment by 2030. Sharing of evidence‐based knowledge and innovation with stakeholders is stated as one way through which the strategy can be implemented (Mastercard Foundation, 2019). So, the youth employment EGM is a useful resource in that regard, as it can guide policy makers, development partners and researchers to relevant available evidence on youth employment interventions.

For researchers, the youth employment EGM has identified research gaps for new primary research and new synthesis. This can inform strategic policy‐oriented approach in commissioning relevant and coordinated research programmes (White et al., 2020).

Before the production of this EGM, two pre‐existing evidence gap maps on youth employment‐related interventions were inadequate in a number of ways. For instance, each of those maps had a narrow scope (geographical, study publication period and, area/interventions and outcomes). The maps were limited to low‐ and middle‐income countries and publication period of 1990–2015. Moreover, development interventions are often best appreciated and understood in a contemporary context (Mawn et al., 2017). Further, both maps did not include economic outcomes while one did not include welfare outcomes. In addition, at least one of the maps suffered methodological limitation stemming, whereby the study search strategy and screening of studies were conducted by individuals rather in pairs for validation. This is stated to have led to some studies being missed. Never the less, the two pre‐existing maps provided a basis for the development of the current youth employment EGM, with a broader focus (geographical, study publication period and, area/interventions and outcomes). Methodological limitations were also avoided by better planning for sufficient time and human resources.

### 2.4 Objectives

The research question guiding the production of the youth employment EGM was stated as follows: What is the nature and extent of the evidence base of impact evaluations and systematic reviews on youth employment programmes in the world?

The primary objective was to catalogue impact evaluations and systematic reviews on youth employment interventions to enhance discoverability of evidence by decision makers, development patterners and researchers, so as to promote evidence‐based decision making in programming and delivery of youth employment initiatives. This EGM was considered a primary input into the implementation of Mastercard Foundation's strategy titled ‘Africa Works: Mastercard Foundation Strategy 2018–2030’, which pointed out sharing of evidence‐based knowledge and innovation with stakeholders as a key strategy to be used (Mastercard Foundation, 2019). The time frame for the development of the youth EGM ran from the last quarter of 2019 to December 2020.

The five secondary objectives were:

(i) To construct a framework for the classification of youth employment effectiveness studies.
(ii) To identify available evidence, and clusters of evidence on effectiveness studies (impact evaluations and systematic reviews of impact evaluations) on youth employment interventions.
(iii) To create a map of youth employment effectiveness studies equipped with an appealing user‐friendly web‐based search content visualisation using interactive mapping software.
(iv) To produce a narrative report of the youth employment EGM.
(v) To disseminate the EGM to users to increase awareness to support evidence‐informed decision‐making across countries.

### 2.5 Existing EGMs on youth employment interventions

Before the production of this EGM, there were two evidence gap maps on youth employment. The two EGMs were reviewed to inform the development of the framework for this EGM. The descriptions provided below for each of the maps pointed out associated strengthens and limitations which were of scope and methodological nature.

The first evidence gap map was the ‘Youth and Transferable Skills evidence gap map’. The map included 98 studies and is accessible at https://gapmaps.3ieimpact.org/evidence-maps/youth-transferable-skills-evidence-gap-map (Rankin et al., 2015). The map included studies published or made available between 1990 and 2015. The included studies were searched from January to February of 2015. The map is restricted to low‐ and middle‐income countries. In terms of youth employment as a development area, the map has a narrow focus, covering only transferable skills interventions and associated outcomes. For, instance economic outcomes are not covered, yet these set of outcomes for example ‘cost effectiveness’ often have important bearing on the implementation of programmes. The map also suffered methodological problems due to time constraints. It was mentioned that the use of a single specialist to supervise and compile the search work as well as reliance on one person to screen studies on titles and abstracts, could have led to some studies being missed (Rankin et al., 2015). The map had an accompanying published narrative report which provided detailed information on areas such as methodology and results, which is a strength. In addition, this map used an extensive study search strategy covering 34 websites and 4 research registries.

The second map was the ‘Youth employment evidence gap map’, produced by International Labour Organization. The map included 113 studies and is available at: https://gapmaps.3ieimpact.org/evidencemaps/youth-employment-evidence-gap-map (International Labour Organization, 2018). The map was restricted to low‐ and middle‐income countries and included studies published or made available between 1990 and 2014 which were contained in a systematic review by (Kluve et al., 2017), titled ‘Interventions to improve the labour market outcomes of youth: A systematic review of training, entrepreneurship promotion, employment services and subsidised employment interventions’. The map did not include economic and welfare outcomes. In addition, a narrative report accompanying the EGM was not accessible which is an indication of its absence. Although, a narrative report is an optional product in the production of an EGM (Saran & White, 2018), the absence or lack of access to such a document denies users vital information.

The above maps provided a basis for the development of the current youth employment EGM, with a broader focus (geographical, study publication period and, area/interventions and outcomes).

## 3 METHODS

### 3.1 Definition and purpose of EGMs

Saran and White (2018), define an EGM as ‘a systematic [visual] presentation of the availability of relevant evidence for a particular policy domain. The evidence is identified by a search following a prespecified, published search protocol. The map may be accompanied by a descriptive report to summarise the evidence for stakeholders such as researchers, research commissioners, policy makers, and practitioners’ (p. 11). It's important to note that EGMs summarise what evidence exists but not what the evidence says. For instance, an EGM catalogues studies in a particular policy domain in terms of outcomes and interventions but does not say the magnitude of outcomes reported by the studies.

EGMs are useful in many ways. First, they guide policy makers, development partners and researchers to relevant available evidence to inform the design and implementation of development interventions. Decision‐makers and researchers often lack awareness about the extent of evidence base, so maps help in increasing the discoverability, and thus use of that evidence for evidence‐informed decision making in international development policy and practice (White et al., 2020). Second, they create awareness among implementing agencies where relevant evidence for their interventions is lacking, so that they can act accordingly by collecting evidence for the intervention they are supporting. Finally, maps identify research gaps for new primary research and new synthesis. This can inform strategic policy‐oriented approach in commissioning relevant and coordinated research programmes (White et al., 2020).

### 3.2 Framework development and scope

Development of the framework is considered the first and most important part in the development of an evidence map (White et al., 2020). The framework was therefore the first activity undertaken in the production of this EGM, in the last quarter of 2019. The framework provided the structure or layout of the EGM and was a primary resource in the development of the search strategy, screening and coding tools. A typical framework for an effectiveness EGM refers to the matrix of interventions (in rows) and outcomes (in columns), developed through a review of existing maps on a related policy domain, policy literature and consultations with stakeholders (Rankin et al., 2015).

The development of the framework for this EGM was achieved through a consultative process involving authors of the map, Mastercard Foundation and stakeholders in the youth employment area. The consultative approach helped the capture of a wide range conceptual and contextual positions of Mastercard Foundation and stakeholders involved in youth employment programming and implementation. The steps followed are described below.

First, using a workshop approach in Uganda, the EGM authors constructed a draft framework by brainstorming and reviewing existing EGMs that included impact evaluations of interventions to improve youth labour market outcomes and systematic reviews of such single studies. Dr. Howard White who is an expert in development evaluation and Dr. Ekwaro A. Obuku, an expert in evidence synthesis, led this activity.

Second, the draft framework was shared with Mastercard foundation to capture their input. It's important to note that Mastercard Foundation (funder) was engaged all through the project life by ensuring that they reviewed study tools (study screening tool, coding sheet and, a dictionary of outcomes and interventions), that were developed by the EGM authors.

Finally, the EGM authors led by Dr. Saran Ashrita, a methods expert had a training workshop in Uganda which, incorporate Mastercard Foundation's feedback on the draft framework into the final framework. Additional activities that were undertaken in this stage include drafting of the coding sheet, definitions of interventions and outcomes to guide coding of studies. Training of people who coded studies using EPPI Reviewer 4, a web‐based software program for managing and analysing data in literature reviews, was also carried. The framework was also piloted with about 100 studies.

### 3.3 Stakeholder engagement

Meetings and workshops to engage stakeholders were planned to be conducted in Uganda, in the last quarter of 2019. The target stakeholders were relevant officials in Uganda from government ministries, departments, agencies, private sector agencies, civil society organisations, vocational training institutes, international development agencies as well as academia. Unfortunately, the COVID‐19 global lockdown constrained stakeholder engagement.

However, keeping in touch with Mastercard Foundation policy leads and the literature were informative in arriving at reasonable priority list of the interventions and outcomes for this EGM.

### 3.4 How youth employment interventions are supposed to work

This section provides theoretical pass‐ways in which youth employment interventions included in this EGM may put more youth into employment through both job seeking or entrepreneurship. The principal assumption of participating in youth employment interventions is that youth can acquire relevant skills for labour market and, support (financial, guidance and information access to facilitate employment. Participation in youth employment interventions is expected to address constraints to youth employment, some of which, Datta et al. (2018), identifies, as summarised in (Box 1).

Box 1 **Constraints to youth employment**

- Gaps and mismatches in technical, cognitive and socioemotional skills that results from deficient education and training systems.
- Asymmetric information, whereby youth often lack information due to information gaps, little or no work experience and limited access to social networks.
- Lack of assets and limited access to credit; which excludes young people from engaging in productive self‐employment opportunities especially among rural youth and economies where agriculture is the most dominant productive activity.
- Regulatory constraints to hiring youth. Decent work policies can deter employers from hiring young new employees. For instance, employee protection legislation and mandatory social benefits may discourage hiring first‐time job seekers who may be higher risk.
- Limited access to credit and lack of assets. Young people usually have low savings, and limited assets for securing loans from formal financial institutions. These constraints exclude youth from financial inclusion and becoming entrepreneurs.

The Theory of change (ToC) provided in (Supporting Information Annex 8; Figure 17) highlights some of the pathways though which the interventions are expected to lead to outcomes. This approach identifies connections between interventions and outcomes that are contained in the Youth Employment EGM which is the subject of this report. The interventions and outcomes of the EGM later revisited in the methods section in Tables 1 and 2, respectively, are grouped into categories and sub‐categories as well as examples. The three intervention categories are: strengthening training and education systems; enhancing labour markets and; transforming financial sector markets. The following paragraphs attempt to show the linkage between intervention sub‐categories and outcomes, with supporting literature.

**Table 1:** Intervention categories, sub‐categories and examples/descriptions

Category	Subcategory	Example
Strengthening training and education systems	Training, up‐skilling and retraining/re‐skilling	Prior learning assessment and recognition (PLAR)
		Education, technical and vocational training (TVET)
		Internship and apprenticeship
		Training centre accreditation and certification
		Training of trainers and teachers
		Business skills training
		Life skills training
Enhancing labour markets	Support to employment	Employee mentoring (Work integrated learning; on job training)
		Career offices/advisory services/career days
		Programme for overseas employment
		Public works programs
		Support to employee mobility and placements
		Wage subsidies
	Decent work policies	Labour standards
		Social protection and social security
		Accountability systems
	Information	Labour market information
		Digital services and SMS coaching
		Social media campaigns and awareness campaigns
		Value chain development
		Access to services and markets (value chains)
Transforming financial sector markets	Entrepreneurship promotion and financing	Small and medium sized enterprise finance (SME)
		Microfinance (to individuals)
		Social impact bonds
		Crowd funding
		Loan guarantees
		Grants
		Self‐financing groups
		Micro‐franchising

**Table 2:** Outcome categories and sub‐categories

Category	Subcategory
Economic	Costs
	Cost Benefit
	Cost effectiveness
	Multiplier, displacement and spill over effects (Effects not directly in the programme, e.g., youth spending earnings to improve local commerce, employment displacement)
Education and skills	Education completion and qualifications
	Access to/in education
	Education quality
	Technical skills and vocational training
	Digital skills
	Transferable skills (including life and social skills e.g., networking, negotiation)
Entrepreneurship	Access to financial services
	Business creation
	Business performance
	Job creation (Jobs for other people e.g., number of employees)
Employment	Vacancies
	Actively seeking employment
	Employment expectation
	Employment status (including duration)
	Employment consistent with education/training
	Hours worked
	Job quality (includes formal vs. informal here)
	Earnings and salary
Welfare	Economic outcomes (except earnings). This also includes income at household level
	Criminal and delinquent behaviour (antisocial behaviour)
	Citizenship, values and social behaviour [Social behaviour is such things as taking part in community activities (clarifying to distinguish from antisocial behaviour). Social behaviour: alcohol/drugs, hanging out with friends]
	Family health and education
	Inclusion and empowerment (social network). [Engagement in community activities is here (not social behaviour)]

First, the ‘strengthening training and education systems’ category of interventions equip youth with skills which are necessary for increasing employment opportunities in the labour market (United Nations, 2018). Actually globally, skills training is the most widely implemented set of labour market interventions for youth (Kluve et al., 2017). Training programmes often equip youth with skills required by employers. However, training and formal education interventions often do not reach marginalised youth especially young women, indigenous groups, youth with disabilities. This leaves such youth without the skills needed to realise their potential (United Nations, 2018). Skills attained through training can be categorised into technical skills, business skills and life or soft skills (Kluve et al., 2017). Technical skills are achieved by individuals attending training initiatives such as technical and vocational education and training (TVET) and, internship and apprenticeship. Business skills training is normally provided to increase entrepreneurial activities among youth (Kluve et al., 2017). In the case of life skills training, the objective is to strengthen trainees' self‐esteem and work habits to help them achieve the goals set by employers (Lippman et al., 2015). The ‘training and skills development interventions results chain’, diagnostic framework documented by Kluve et al. (2017) is a useful resource for mapping the relationship between training and education intervention and potential outcomes. Participation in training and education is expected to create mainly three categories of outcome: (i) employment outcomes (i.e., change in employment status, earning and salary); (ii) education and skills outcomes such as acquisition of technical and vocational skills.

Second, the ‘enhancing labour market’ category of interventions are grouped into three sub‐categories including: support to employment; enhancing labour market; and transforming financial sector market. Support to employment interventions are generally meant to help youth find jobs through provision of jobs via initiatives like programmes for overseas employment and public works programmes. Employment services also facilitate the youth in the process of finding jobs through career guidance and supporting employee mobility needs. Decent work polices generally regulate the relationship between employers and employees in the employment environment through application of labour standards, regulations and accountability systems. Another enhancer of labour markets, is provision of a wide range information about the labour market and associated services. Potential outcomes from this category of interventions tend to be largely associated with employment outcomes such as job quality and change in employment status.

Finally, ‘transforming financial sector’ category of interventions focus on entrepreneurship promotion and financing. The category of interventions can be considered the most popular for targeting disadvantaged youth especially those excluded from training and education programmes. Datta et al. (2018) note that labour market opportunities are significantly influenced by the reverence of skills for the existing job market. An intervention like self‐help or financing groups tends to be dominant among youth with less education and training. Self‐help groups are small groups that save a certain amount of money on weekly or monthly basis and issue loans to members out of their collective savings (Flynn, 2013). The category of interventions (transforming financial sector) tend to be mainly associated with welfare outcomes and employment outcomes than economic outcomes.

### 3.5 Dimensions

The primary dimensions of the map are interventions (in rows) and outcomes (in columns), presented in a matrix. There are three broad intervention categories, each with subcategories. The intervention categories include: Strengthening training and education systems; enhancing labour markets; and transforming financial sector markets. The outcomes are arranged in a typology of five categories: education and skills, entrepreneurship, employment, welfare, and economic. The interventions and outcomes are later described in detail in Tables 1 and 2, respectively.

The secondary dimensions of the map are the taskbar menu (Filters, About and View records), which help a user to navigate the map. A detailed description of the taskbar menu is provided later below (Figure 4).

### 3.6 Types of study design

Only studies with the following study designs were included in the EGM: Experimental designs, Nonexperimental matching designs, Regression‐based approaches and, Systematic reviews.

The selected study designs are the appropriate designs for estimating effectiveness of program interventions. The designs (experimental and nonexperimental) are for conducting impact evaluations of development interventions. Examples of the following non‐evaluation‐based study designs were excluded from the EGM: ethnography, case control and cross‐sectional. For instance, the focus of a cross‐sectional study is limited to data from a particular population with variables of interest, at a given point in time. Cross‐sectional studies are observational in nature and not causal implying that they are not applicable for determining the effect of an intervention.

(a) Experimental designs

Experimental designs fall into two types, namely randomised controlled trials (RCTs) and natural experiments.

(i) RCTs: A typical RCT design involves randomising study participants into two or groups (an experimental/treatment/intervention group and control group) in which the researcher introduces an intervention and measures its impact on the dependent variable at least two times namely pre‐ and posttest measurements (White & Sabarwal, 2014). A key weakness of an RCT study design is that it suffers from missing outcomes resulting from changes that occur postrandomisation of study participants. For instance, withdrawal of subjects from the study and noncompliance with established study protocols or guidelines would lead to missing outcomes (White & Sabarwal, 2014). Therefore, application of Intention‐to‐treat (ITT) analysis in RCTs attempts to address this problem by including in the analysis every subject who is randomised according to randomised treatment assignment and ignoring anything that happens after randomisation (Gupta, 2011). So, studies using ITT analysis have been included in the EGM under RCT study design.
(ii) Natural experiments: Despite the lack of universally accepted definition of the term natural experiment, researchers are in agreement that a natural experiment happens where and/or when an intervention is implemented without the control of a researcher (Butler et al., 2018). Therefore, a natural experiment is an experiential study design in which clearly defined sub‐populations are exposed to the experimental and control conditions that are determined by nature or other factors outside the control of the researchers. The allocation into experimental and control conditions allows researchers to use natural or unplanned variation in exposure to draw inferences about causality.

For example, a policy development emphasising promotion of TVET in Uganda can be seen as a natural experiment. TVET is a school‐based intervention, which makes it a commonplace intervention and thus underpinned by equity considerations. Therefore, a randomised experiment/RCT design is often considered to be politically and ethically not feasible. In this case a natural experiment is a preferred evaluation study design.

(b) Nonexperimental matching designs

Nonexperimental designs are used where random assignment is not feasible for various reasons. For instance, where the need for evaluation arises when the program is completed or on going. Nonexperimental designs can be generally categorised as quasi‐experimental and regression‐based approaches. Quasi‐experimental methods create comparison groups by statistical methods, rather than by random assignment. These methods include difference‐in‐differences (DiD), propensity score matching (PSM), regression discontinuity designs (RDD), and synthetic controls designs (White & Raitzer, 2017).

(i) PSM: In PSM, the matching enables construction of an artificial comparison group with almost similar characteristics as the treatment group. The artificially created comparison group from untreated observations is matched to treatment observations from the untreated sample, based on observable characteristics. The treated units are matched to untreated units with a similar propensity score. The matching approach is considered adequate to attain an unbiased impact estimate (White & Sabarwal, 2014).
(ii) DiD: In DiD approach, impact is estimated by comparing the changes in outcome over time between treatment and comparison groups. The method is also known as controlled before and after studies or ‘double difference’ method (White & Sabarwal, 2014).
(iii) RDD: This is a popular approach used in econometrics due to situations that make randomisation unfeasible to determine causal effects of interventions by assigning a cut‐off or threshold above or below which an intervention is assigned. The threshold refers to the criterion that participants must met before being included in the intervention. The threshold is usually based on a continuous variable (White & Sabarwal, 2014). For instance, adults above or below a particular age for enroling in a training programme. RDD approach compares observations on either side of the threshold to estimate average treatment effects of an intervention. The major limitation of RDD is that its greatly affected by confounding variables. For instance, average treatment effects of a local sanitation intervention may be affected by a regional related intervention if they were implemented at the same time.

(c) Regression‐based approaches

All approaches based on regression models not listed above are included here. These include (but are not restricted to):

(i) Instrumental variable (IV): ‘A statistical technique for estimating causal relationships when an RCT is not feasible or when an intervention does not reach every participant/unit in an RCT’ (White & Sabarwal, 2014, p. i).

(d) Systematic reviews

A systematic review summarises bodies of literature with summary statements of the findings of that literature. The term ‘systematic’ in systematic review refers to the research process that is intended to minimise the biases that may occur in a traditional literature review. Key characteristics of a systematic review include: a clear scope for the review; set of research questions; clear study inclusion and exclusion criteria; systematic search strategy used to identify the single studies that would meet the eligibility criteria; and results. Therefore, studies included in the EGM as systematic reviews had the above‐mentioned characteristics even if they did not use the term ‘systematic’ in the titles. Studies using the term ‘systematic’ but lack key features of a systematic review were excluded. For instance, scoping reviews or literature scans and Meta‐analysis of evidence were excluded on study design criterion.

### 3.7 Types of intervention/problem

The EGM has three broad intervention categories, each with subcategories. The intervention categories include: Strengthening training and education systems; enhancing labour markets; and transforming financial sector markets. Table 1 lists the intervention categories, subcategories as well as examples to aided study search and coding. The intervention category of ‘strengthening training and education systems’, here refers to a group of sub‐interventions covering improvements in training, upskilling and retraining or re‐skilling components of education and training systems. Specifically, up‐skilling is the process of teaching workers new skills. Retraining is a practice where employers may ensure that their workers learn new skills specially to avoid stagnant workforce. So here, some examples of interventions to be captured include TVET, business skills training and, internship and apprenticeship (Table 1).

A detailed compilation of definitions of the interventions are provided in Supporting Information Annex 1, with a reference list provided as Supporting Information Annex 3.

### 3.8 Types of population

The only criterion used for selecting the target population is age. So, the target population is all youth or young women and men aged 15–35 years from all countries. The diversity of varying national definitions of the term youth was acknowledged. For instance, while the United Nations (UN) defines youth as young women and men aged 15–24 years, in South Africa youth fall in the age group 14–35 years (South Africa, 2015), in Zimbabwe it's from 15 to 35 years of age (Zimbabwe. Ministry of Youth Development, 2000) and in Uganda youth are within the age bracket of 15–30 years (Uganda. Ministry of Gender Labour and Social Development, 2016). This EGM uses a more encompassing classification to identify youth as young women and young men aged between 15 and 35 years.

Population subgroups included: both females and males; youth with disabilities; youth in fragility, conflict and violence (FCV) contexts; youth from disadvantaged background (low‐income families or low education); criminal background; ethnic minorities; and humanitarian settings. The population sub‐groups permitted the identification of studies reporting evidence on equity.

### 3.9 Types of outcome

Table 2 contains outcome categories and subcategories. The outcomes are arranged in a typology of five categories: education and skills, entrepreneurship, employment, welfare, and economic. For instance, the education and skills outcomes refer to notable achievements in education, satisfaction with quality of education programs; transformative experiences, career readiness and performance.

The welfare outcomes are flagged to ensure that even welfare outcomes not directly associated with employment are captured. For instance, some welfare outcomes (criminal and delinquent behaviour as well as citizenship values) may happen as result of participating in an intervention, though such youth could still be unemployed. The protocol also provided for inclusion of adverse and unintended outcomes in the map. That was important to avoid one‐sided summaries of the evidence. An example of unintended employment outcomes include youth offending such as, increased rate of alcohol abuse due to income resulting from change in employment status. An example of an adverse outcome is accidents and disease resulting from employment hazards. Multiplier effects were also coded. These refer to effects not directly from the programme such as job displacement and youth spending earnings to improve local commerce. Job displacement in this case refers to the loss of jobs by current employees due to the recruitment of new workers for example with more relevant skills for the employer.

Definitions of outcomes are provided in Supporting Information Annex 2, with associated reference list provided as Supporting Information Annex 3.

### 3.10 Eligibility criteria

**Figure 1:**
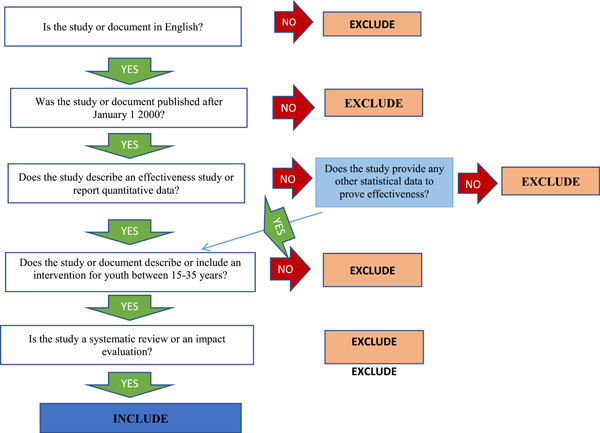
Screening tool

A review of 15 agencies by Saran and White (2018), established that the inclusion criteria for EGMs generally follows the PICOS approach of population, intervention, relevant comparison groups, outcomes and study design. Therefore, the study inclusion criteria for this EGM also used a similar approach. Table 3 shows a summary of PICOS. Additional criterion of study publication period was included. The document should have been published or made available between January 2000 and December 2019. The inclusion and exclusion criteria is illustrated in (Figure 1).

**Table 3:** Summary description of PICOS elements in the EGM

PICOS element	Description
Population	All youth or young women and men aged 15–35 years from all countries.
Intervention	All interventions that fall under: strengthening training and education system; enhancing labour market and; transforming financial sector market.
Comparison	Active or passive (placebo or non‐intervention) alternate intervention in the comparison group.
Outcomes	All outcomes categorised under economic, education and skills, entrepreneurship, employment, and welfare.
Studies	The studies were impact evaluations of youth employment interventions or systematic reviews, which included studies on youth employment interventions.

Unpublished studies were included in the EGM to reduce the effects of publication bias, which refers to the failure to publish a study on the basis of the strength of the study findings.

For studies with interventions that combined youth and non‐youth populations (older adults and children), majority (over 51%) of the study population had to be youth. In addition, studies containing only subsets of eligible interventions were included in the map only when revenant outcomes were reported.

For a single study with multiple reports, it was included in the EGM multiple times only if the study employed different eligible study designs or reported different outcomes. However, if the multiple reports of a single study were for example a working paper and journal article, only one with the most detailed information (interventions and outcomes) was included in the EGM. For instance, where a journal article presented partial outcomes, a working paper was included in the EGM.

### 3.11 Types of settings

The coding tool provided for the following types of settings for interventions: high school, tertiary education institutions, training centres, firms, and the option for others identified when coding studies.

### 3.12 Search methods and sources

The Protocol which is the basis for the production of the Youth Employment EGM is published in the Campbell Systematic Reviews database (Apunyo et al., 2021).

#### 3.12.1 Search strategy

A standardised search strategy provided in Supporting Information Annex 4 was used to search over 20 databases and institutional websites, in English. The rationale for limiting the strategy to the English language documents was based on the consideration that the vast majority of studies were written in English and, translation from other languages to English would be compromised by comprehension limitations.

The search strategy was developed from the entire coding sheet (Supporting Information Annex 5), containing filters (i.e., population demographics and socioeconomic characteristics); selected study designs; interventions and; outcomes. For instance, impact evaluation‐based study designs were included in the search terms, since the focus of the map was effectiveness of youth employment interventions.

While scholarly databases identified peer reviewed articles, institutional websites provided mainly grey literature in the form of evaluation reports and working papers. All identified studies were screened on title and abstract as well as on full text. Completed but unpublished or studies with midterm outcomes were included in the map.

The search strategy was pre‐tested multiple times and peer reviewed by the two information science specialists. After ascertaining the effectiveness of the search strategy, the final search was performed on 29 January 2020. The studies were then uploaded into EPPI Reviewer 4 software, for screening and coding. The confinement of publication period to two decades (2000–2019) was informed by an observation that development interventions are often best appreciated and understood in a contemporary context (Mawn et al., 2017).

#### 3.12.2 Additional search methods

To ensure the more comprehensive identification of studies included in the EGM, additional three activities (searching systematic reviews, snowballing and citation tracking), described below were undertaken. These activities were carried out towards the end of the project, after coding studies identified by the search strategy.

There was a search within reference lists of the 21 systematic reviews included in the EGM and existing evidence gap maps on youth employment. There was no systematic approach of contacting individuals and organisations to access full articles that were not accessible online. Due to the big number of studies included in the map, a targeted search of reference lists of included studies was conducted through snowballing and citation tracking.

Snowballing and citation tracking were conducted after studies identified by the standardised search strategy and searching within systematic reviews had been coded. While snowballing involved reference tracking the 20 most recent publications, citation tracking was limited to the 10 most current publications. Snowballing involved searching the reference lists of included studies and identifying studies that met the eligibility criteria of the study. Citation was conducted in ‘Google Scholar’ by pasting the ‘reference text’ of each study in google scholar search area to show a list of studies which had cited that particular study.

### 3.13 Data collection

#### 3.13.1 Screening and study selection

Screening of studies was carried out in EPPI‐Reviewer 4, which is a web‐based software program for managing and analysing data in literature reviews. The study references identified from databases searched, were imported into EPPI‐Reviewer 4, where duplicates were removed before screening. For studies identified through searching reference lists of systematic reviews, snowballing and citation tracking, bibliographic information was manually captured in EPPI Reviewer 4.

Studies were screened using a five‐criteria screening tool developed by Dr Howard White, an expert in impact evaluation and evidence synthesis. Included studies were those written in the English; published or made available after 1 January 2000 and by 31 December 2019; described an intervention for youth between 15 and 35 years and; should have been a systematic review or an impact evaluation (Figure 1).

There were two levels of screening studies, on the basis of titles and abstracts and on full texts. At first level, titles and abstracts were screened independently by each of the two reviewers against the inclusion criteria. A reconciliation report comparing the results of the two reviewers was generated from EPPI Reviewer‐4 for identification of disagreements which were resolved through discussion by the reviewers. To add rigour, where the two reviewers did not reach consensus the matter was forwarded to the third reviewer. At second level, full text papers were again screened by two reviewers independently and disagreements reconciled through discussion as in the first level of screening.

The screening tool was piloted through a number of sessions with each of the sessions using about 100 studies. In the first session reviewers were trained at Africa Centre for Systematic Reviews and Knowledge Translations, College of Health Sciences—Makerere University in Uganda, by Dr. Ashrita Saran, a methods expert.

#### 3.13.2 Data extraction and management

The studies were coded on the basis of the information contained in the coding sheet, provided in Supporting Information Annex 5. Guidance was provided to reviewers involved in coding the studies, through piloting coding and checklists. For instance, reviewers used the most current World Bank classification of countries by income level to code the World Bank Regions. A dictionary defining interventions and outcomes was also provided for reviewers involved in coding studies.

The coding sheet was piloted before full scale coding of studies. Five piloting sessions were conducted. In each session, each study was independently coded by a pair of reviewers. After each pilot session, the entire EGM team discussed the results of pilot coding to humanise the application of the coding sheet. Post pretest coding of studies was conducted again by pairs of reviewers who reconciled disagreements through discussion. Where the two coders did not reach consensus, the mater was forwarded to the third reviewer/tie breaker.

### 3.14 Quality appraisal of studies/risk of bias

Critical appraisal of each study (impact evaluations and systematic reviews) was conducted independently by a pair of reviewers who again followed the same procedure used at screening and coding phases, to reconcile disagreements.

Impact evaluation studies were assessed using the ‘Quality assessment of Impact Evaluations’ tool developed by Dr. Howard White and Dr. Saran Ashrita. The tool is a checklist of seven items with additional guidance on rating items, expressed as: high confidence, medium confidence or low confidence. However, of the seven items only four (study design (potential confounders taken into account); level of attrition or losses to follow up^2^; definition of outcomes; and baseline balance^3^ reports), were the most critical for making decisions.

A Measurement Tool to Assess systematic Reviews (AMSTAR 2) was used to conduct critical appraisal of systematic reviews. AMSTAR has been developed to facilitate the development of high‐quality reviews by guiding the conduct and evaluation of reviews. The AMSTAR 2 checklist^4^ contains 16 items, each with concise sentence questions having supplementary guidance on selecting response options (expressed as: ‘yes’, partial yes and ‘no’). Overall, the AMSTAR 2 tool rates confidence in components of a systematic review as; High: no or one no‐critical weakness, Moderate: more than one noncritical weakness, Low: one critical flaw with or without noncritical weaknesses and, Critically low: more than one critical flaw with or without noncritical weaknesses (Shea et al., 2017).

### 3.15 Methods for mapping

EPPI Reviewer 4—a web based software program for managing and analysing data in literature reviews (EPPI Centre, n.d.) was used for screening and coding studies as well conducting analysis. Screening and coding was based on predefined codes extracted from the eligibility criteria and coding sheet provide in Supporting Information Annex 5. The study flow diagram is presented later in Figure 5. The analysis involved generating frequency tables, cross‐tabulated tables, graphs and charts.

EPPI Mapper was used to generate the EGM. EPPI‐Mapper is a tool for visualising ‘maps’ of research evidence (EPPI Centre, n.d.). The tool is used generate an evidence and gap as follows. After coding data in EPPI Reviewer, a JSON file form is exported and uploaded to EPPI Mapping tool to generate the EGM, via: http://eppimapper.digitalsolutionfoundry.co.za/.

### 3.16 Analysis and presentation

#### 3.16.1 Report structure

The report structure provided below is an outline containing major sections with included tables, figures and boxes, in the main body of the report. Additional tables, figures and boxes are provided in the annexes as Supporting Information.

**Table:** 

**Figure 2:**
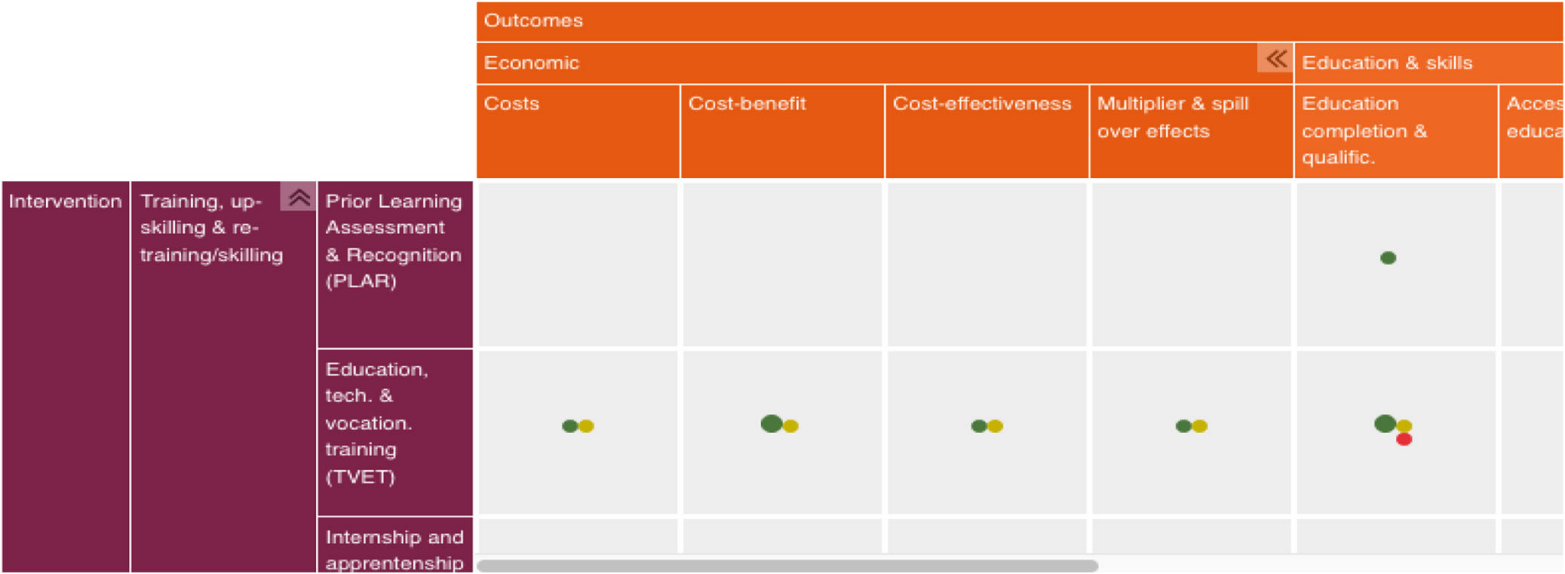
Snapshot of a section of the Youth Employment Evidence and Gap Map. *Source*: https://youthfuturesfoundation.org/our-work/identify/evidence-and-gap-map/

**Figure 3:**
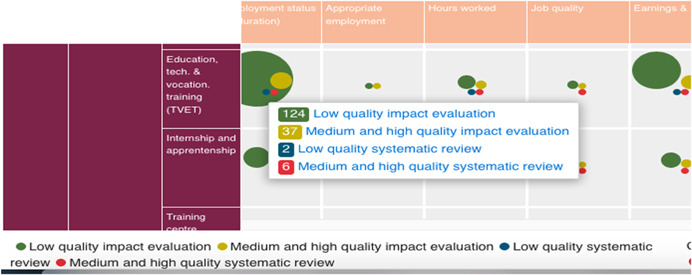
Hovering over a cell to get a list of studies. *Source*: https://youthfuturesfoundation.org/our-work/identify/evidence-and-gap-map/

**Figure 4:**
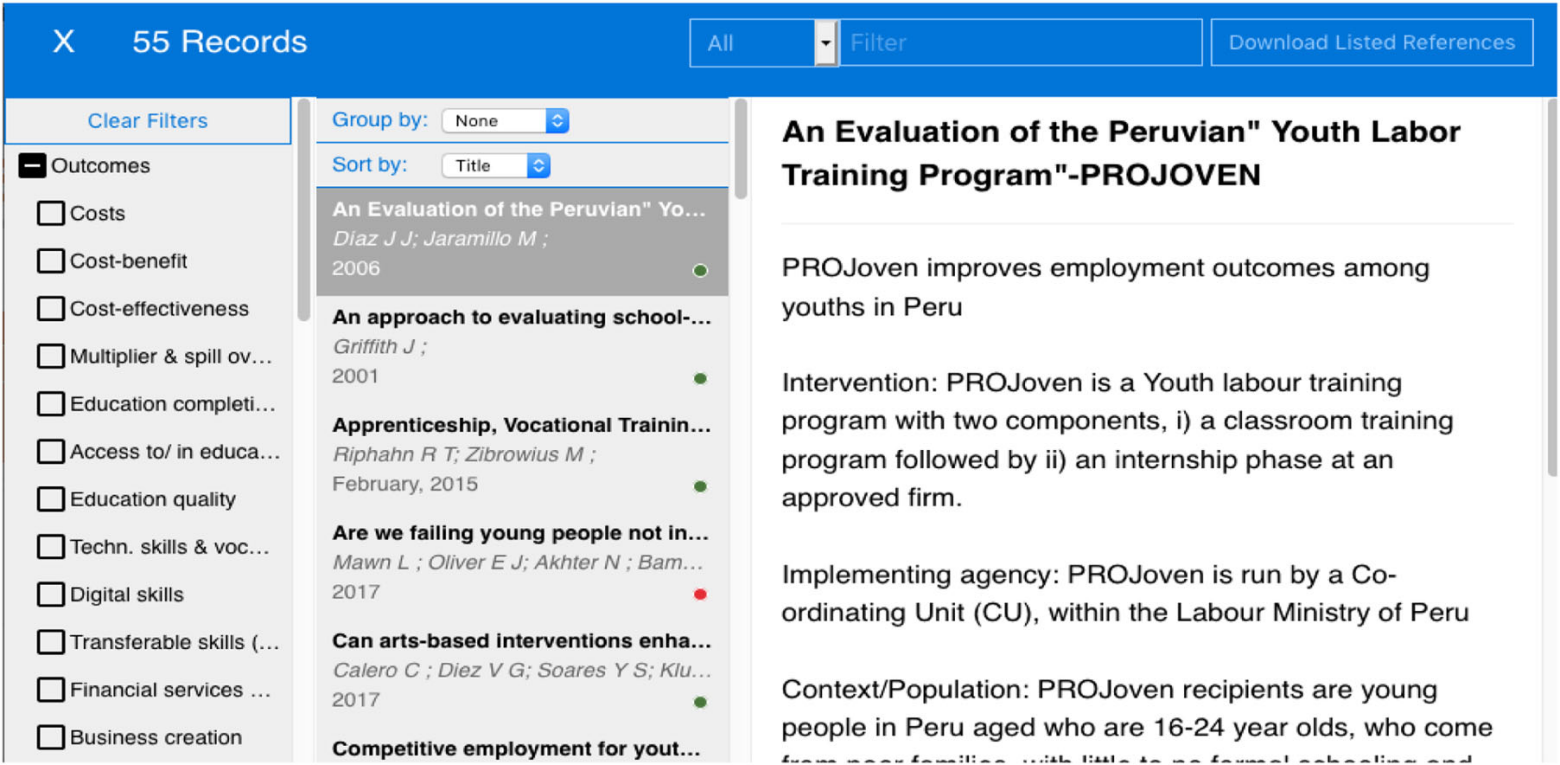
Clicking on a cell gives a list of studies. *Source*: https://youthfuturesfoundation.org/our-work/identify/evidence-and-gap-map/

**Figure 5:**
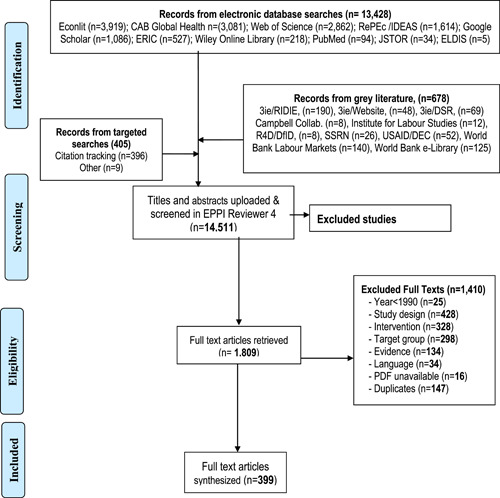
Flow diagram for Youth Employment Evidence and Gap Map

**Figure 6:**
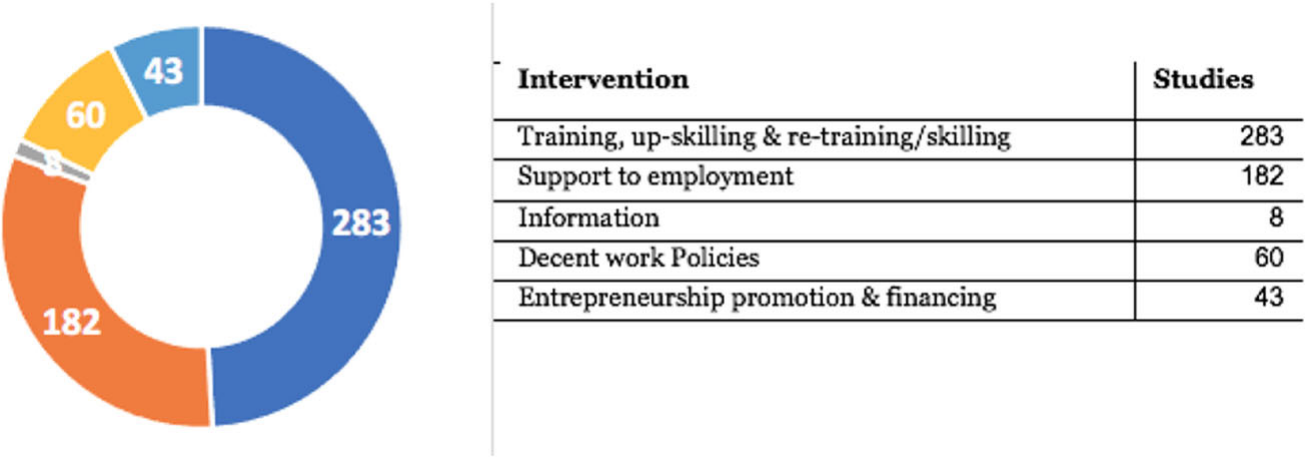
Interventions sub‐ categories, number of studies

**Figure 7:**
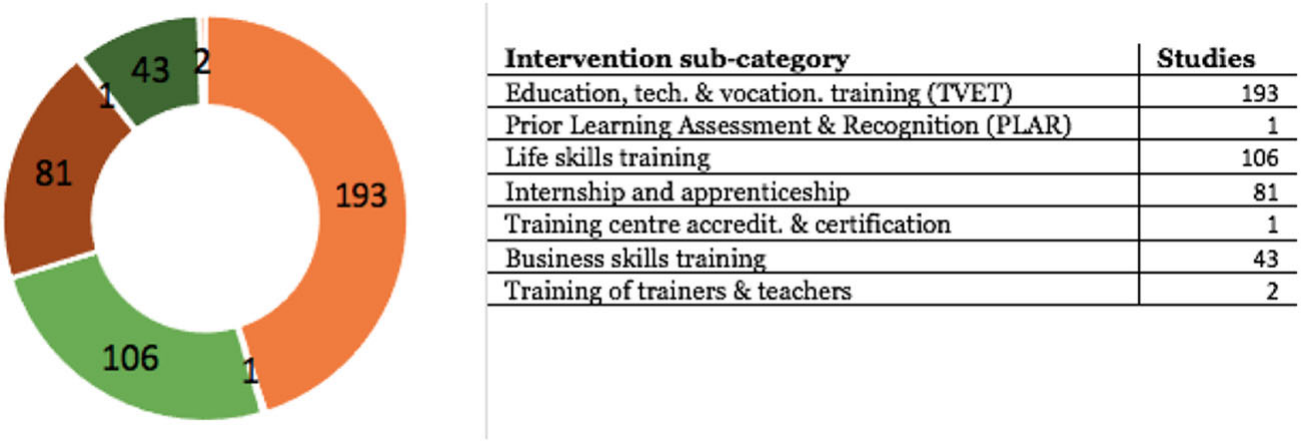
Intervention sub‐categories for the ‘training and up‐skilling’ category, (number of studies)

**Figure 8:**
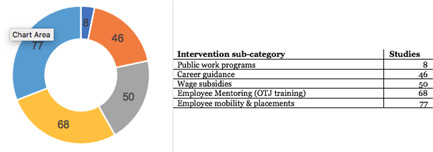
Intervention subcategory for Support to employment

**Figure 9:**
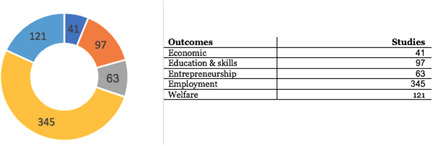
Outcome categories, number of studies

**Figure 10:**
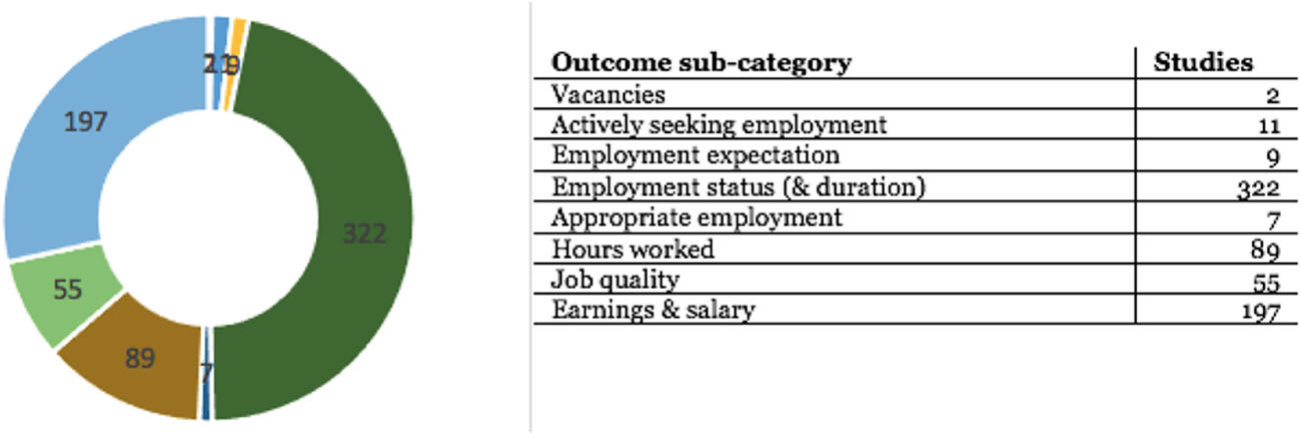
Employment outcomes domain

**Figure 11:**
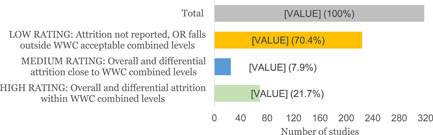
Attrition rating of impact evaluations

**Figure 12:**
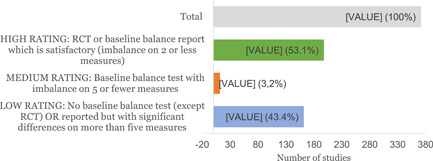
Baseline balance tests of impact evaluations

Abstract
Plain Language Summary
Background
Objectives
Methods
Table 1: Intervention categories, sub‐categories and examples/descriptions
Table 2: Outcome categories and sub‐categories
Table 3: Summary description of PICOS elements in the EGM
Table 4: Reasons for exclusion of studies on full texts
Figure 1: Screening Tool
Figure 2: Snapshot of a section of the Youth Employment EGM
Figure 3: Hovering over a cell to get a list of studies
Figure 4: Clicking on a cell gives a list of studies
Results
Table 4: Reasons for exclusion of studies on full texts
Box 2: Examples of excluded studies
Table 5: Types of evidence
Table 6: Evidence types by income status based on World Bank classification
Table 7: Setting for interventions and, Sectors of interventions
Table 8: Intervention implementer
Table 9: Evidence types by social population factors
Table 10: Evidence about regions of the world by social population factors
Table 11: Multi‐component youth employment interventions
Table 12: Aggregate map of evidence gaps
Figure 5: Flow diagram for Youth Employment Evidence and Gap Map
Figure 6: Interventions sub‐ categories, number of studies
Figure 7: Intervention sub‐categories for the ‘training and up‐skilling’ category
Figure 8: Intervention sub‐category for Support to employment
Figure 9: Outcome categories, number of studies
Figure 10: Employment outcomes domain
Figure 11: Attrition rating of impact evaluations
Figure 12: Baseline balance tests of impact evaluations
Risk of Bias in Included Studies
Table 13: Quality of evidence/risk of bias of included studies
Table 14: Quality of evidence by intervention sub‐categories and outcome categories
Table 15: Critical flaws in systematic reviews rated as low quality
Discussion
Potential Biases in the Mapping Process
Conclusions
Acknowledgements
Roles and Responsibilities
Funding
Potential Conflicts of Interest
Plans for updating the EGM
Differences between the Protocol and EGM
Link to online interactive EGM
References
Annexes

#### 3.16.2 Dependency/unit of analysis

The unit of analysis for the report is individual studies where every entry represents a combination of interventions and outcomes. The findings are descriptive, showing the distribution of studies in terms of study design, regions of world, study quality, settings, interventions and outcomes. The aggregate map is presented in a coloured table showing well‐evidenced areas and low evidenced areas.

#### 3.16.3 Filters and presentation

The filters are populations groups such as disadvantaged youth and age groups of youth; regions of the world by income status; countries; study settings and; implementers of interventions. The interactive EGM is presented as follows.

The youth employment EGM is a matrix of interventions (in rows) and outcomes (in columns), populated with studies that provide evidence for each cell's outcome and intervention combination. Each study was placed in each cell for which the study provides evidence. That means that majority of studies appear in the map multiple times because they have multiple outcomes and interventions. Each study has been tied to a weblink which directs the user of the map to an online database where the full text or paper of the study is uploaded. The map has primary and secondary dimensions which provide an appealing user‐friendly content visualisation.

The primary dimensions of the map are interventions (in rows) and outcomes (in columns), presented in a matrix. Figure 2 shows a snapshot of a section of Youth Employment EGM. Interventions were grouped into categories, subcategories. The outcomes are arranged in five categories: education and skills, entrepreneurship, employment, welfare and economic. For instance, the ‘economic’ category of outcomes contains the following outcome subcategories: Cost, cost benefit, cost effectiveness and multiplier effects. In the case of interventions, the ‘training’ category comprises; TVET; internship and apprenticeship; Training centre accreditation and certification; training of trainers and teachers; business skills training; and life skills training.

The map is interactive. By hovering over a cell, you can see the number of studies in that cell. At the bottom of the map, the colour‐coding of bubbles represents study quality based on critical appraisal of included studies. Study quality ratings include: low quality impact evaluation; medium and high‐quality impact evaluation; low quality systematic review; or medium and high‐quality systematic review. Pointing the cursor at the cell, identifies single studies from systematic reviews, according to study quality rating (Figure 3).

By clicking on a cell, you can see a list of the studies in that cell. Clicking on a study in the list (middle panel) gives a summary of that study as well as the URL which is a gateway to that study (Journal database or website), where the uploaded paper can be assessed. Clicking on a row or column heading gives the list of studies in that row or column. By checking a filter (left hand side) you can filter studies displayed (Figure 4).

The secondary dimension of the map is the taskbar menu (Filters, About and View records), which help a user to navigate the map. Filters are search aids which help the user of the map to quickly find records matching criteria of interest. The filters include study design, regions of the world, country and population subgroups. The population subgroups include youth with disabilities, youth in FCV contexts and, youth from disadvantaged background (low‐income families or low education); criminal background; ethnic minorities and; humanitarian settings. Clicking on the ‘About’ tab in the taskbar menu displays the ‘about’ text which describes the map. Clicking on the ‘View Records’ tab displays a record of all studies in the map, offering the user options to export the reference list which is compatible with other reference management software such as Endnote and EPPI Reviewer.

## 4 RESULTS

The results section covers the following broad sections: Description of studies, Synthesis of included studies, Risk of bias in included studies and, Discussion of the results. The presentation is aided by tables and figures provided in the main document as well as those contained in the Annexes, as supporting information.

### 4.1 Description of studies

#### 4.1.1 Results of the search

Evidence was mapped for the period of two decades, 2000 to 2019. More evidence was available in the last decade, with the period 2014–2019 accounting for over 38% of included studies. There was a positive trend in the number of studies made available or published over the two decades (Supporting Information Annex 8; Table 16).

There were 14,511 studies uploaded into the EPPI Reviewer‐4 management software. Majority of studies were uploaded into the software for screening in January 2020 after validation of the search strategy by the two information science specialists who developed and conducted the search strategy. These studies were majorly from 20 databases especially from Econlit: https://www.aeaweb.org/econlit/, Web of Science: http://Thomsonreuters.Com/Social-Sciences-Citationindex/, CAB Global Health (Ovid): https://www.ovid.com/product-details.30.html, RePEc (Research Papers in Economics)/IDEAS Economics and Finance Research: https://ideas.repec.org/and ERIC: https://eric.ed.gov/. These altogether contributed 13,428 articles. A full list of databases searched is provided in supporting information (Supporting Information Annex 4).

A total of 1,809 titles and abstracts (12.5%) were included for full text screening of which only 399 (22.1%) made it to the final coding and synthesis. The review flow details are illustrated in the EGM PRISMA diagram (Figure 5). A list of included studies, with quality rating is provided in Supporting Information Annex 7.

#### 4.1.2 Excluded studies

The reasons for exclusion of studies at full text screening are captured in Table 4. The reasons were mainly, ineligible study designs (*n* = 428), inappropriate interventions (*n* = 328) or target group (*n* = 298). Box 2 shows examples of excluded studies a reader might reasonably have expected to find in the EGM. The reasons for exclusion of these studies are: ineligible study designs and lack of interventions.

**Table 4:** Reasons for exclusion of studies on full texts

Studies	Number	Share (%)
Include	399	22.1
Exclude	1410	77.9
Study design	428	30.4
Target group	298	21.1
Language	34	2.4
Intervention	328	23.3
Year	25	1.8
Evidence	134	9.5
Duplicates	147	10.4
PDF inaccessible	16	1.1
Total	1809	100

Box 2 **Examples of excluded studies**

**Table:** 

**Author**	**Title**	**Excluded on**
Gonzalez Pandiella, A. (2013)	Getting Irish Youth on the Job Track (No. 1101). OECD Publishing.	Study design.
		Intervention.
Ajwad et al. (2014)	The skills road: skills for employability in Tajikistan.	Intervention.
Angel‐Urdinola, D. F., Belghazi, S., & Hilger, A. (2013)	Building effective employment programs for unemployed youth in the Middle East and North Africa.	Intervention
Audas, R., Berde, E., & Dolton, P. (2005).	Youth unemployment and labour market transitions in Hungary. Education Economics, 13(1), 1‐25.	Intervention.

### 4.2 Synthesis of included studies

#### 4.2.1 Types of evidence

By types of evidence, impact evaluations designs (*n* = 378) were much more than the systematic reviews (*n* = 21). The leading impact evaluations were experimental studies (*n* = 177), closely followed by nonexperimental matching (*n* = 167) and other regression designs (*n* = 35) (Table 5).

**Table 5:** Types of evidence

Studies	Studies	Percent
Systematic reviews	21	5.3
Experimental	177	44.4
Nonexperimental	167	41.9
Other regression	35	8.8
Total	399	100.3^a^

^a^ Percentage is more than 100 as an impact evaluation may be coded more than once for study designs.

Experimental studies were the most conducted in Lower‐income countries and Lower Middle Income countries while nonexperimental study designs were the most common in High Income and Upper Middle Income countries (Table 6). Among the experimental studies, randomised controlled trials (RCTs) represent the highest quality of impact evaluations when done properly. Taken together, most RCTs were conducted in sub‐Saharan countries (*n* = 42), followed by the Americas (north and south), with *n* = 38 and *n* = 24, respectively. In sub‐Saharan Africa, Uganda had the leading number of RCTs (*n* = 16) (Supporting Information Annex 8; Table 17). USA (*n* = 13, 61.9%) and India (*n* = 8, 38.1%) were the countries studied most in the systematic reviews (*n* = 21) (Supporting Information Annex 8; Table 18).

**Table 6:** Evidence types by income status based on World Bank classification

		Impact evaluations (number)		
Income status	Systematic reviews	Experimental	Nonexperimental	Other regression
Low‐income	5	35	5	1
Lower‐middle	7	25	10	4
Upper‐middle	9	36	38	10
High‐income	19	84	115	20

*Note*: Numbers in this table do not add to the actual number of respective types of studies/evidence as an impact evaluation or systematic review may be coded more than once for income regions. For instance, cross‐country studies such as systematic reviews may be coded for more than one region.

Geographically, Europe and Central Asian studies were the most predominant (*n* = 152), with the MENA region having least representation *n* = 17 and therefore with most glaring evidence gaps in the literature of youth employment. There was a relationship between volumes of evidence and World Bank income regions. High‐income country studies were more (59%), with low‐income countries accounting for only 11.5% of the evidence base (Supporting Information Annex 8; Table 19).

The top 10 countries contributed about 64.2% of the studies, with the USA leading and having double the number of studies for Germany, the second ranked out of a total number of 94 countries studied. Among these Peru, India and Uganda were the countries with most studies in Latin America, Asia and sub‐Saharan Africa, respectively. Argentina, South Africa, Colombia and France were the other top 10 countries (Supporting Information Annex 8; Table 20).

In terms of authorship, studies were ranked by author, country and region of the world. There were 45 authors with two or more publications with Europe accounting for 46.7% while Africa and Asia each disproportionately contributing only 4.4% (Supporting Information Annex 8; Table 21). A total of 13 authors had three or more publications. The top 13 authors were from USA (*n* = 6), Germany (*n* = 3), France (*n* = 2) with UK and Argentina having one author each in the top 13. These top 13 authors were responsible for 55 publications of the included studies (13.9%) (Supporting Information Annex 8; Table 22).

#### 4.2.2 Setting for interventions and, sectors of interventions

Settings for interventions were concentrated in firms (*n* = 205). Other intervention settings were training centres (*n* = 154), tertiary education (*n* = 44) and high schools (*n* = 37). Most studies did not report sectors (*n* = 307). Among those that did, the services sector was the most studied (*n* = 74) followed by the nonmanufacturing (*n* = 58) and agriculture (*n* = 33) came fourth after the manufacturing sector, *n* = 43 (Table 7).

**Table 7:** Setting for interventions and sectors of interventions

Settings for interventions	Studies
High School	37
Tertiary Education	44
Training centre	154
Firm	205

**Sectors of interventions**	**Studies**
Agriculture	33
Services	74
Industry: Nonmanufacturing	58
Industry: Manufacturing	43
Sector not reported	307

#### 4.2.3 Implementers of interventions

Looking at implementers, most (85.7%) or 342 of 399 studies reported implementers of interventions. The majority of the interventions were implemented by the government (66.7%). The other implementers included: Government, Business/Industry, private enterprises, researchers, training institutions, NGOs, CBOs, Multinational organisations, Not for profit organisation and individual labour migrant (Table 8).

**Table 8:** Intervention implementer

Implementers of interventions	Studies	Share (%)
Business/Industry	3	0.9
Government	228	66.7
Government and private	13	3.8
NGO and Government, Researchers and Training institution	31	9.1
NGO, CBOs and Multinational Organisations	46	13.5
Private sector, Not for profit organisation and individual labour migrants	21	6.1
Total	342	100.0

#### 4.2.4 By population

Overall, literature was sparse about youth in fragile, conflict and violence‐affected contexts; humanitarian settings, ethnic minorities and those with criminal backgrounds.

Looking at age and gender, youth between 15 and 19 years old commanded the evidence base with an even gender distribution. Where gender was specified it was approximately 80% for both male and female (Supporting Information Annex 8: Table 23). Evidence was substantially less available for ‘older youth’ groups, specifically 30‐35 years compared to those below 25 years.

Evidence gaps were also identified in terms of social factors of all the youth. There is rich literature about youth from disadvantaged backgrounds (*n* = 139) (low‐income families or low education), followed by those disabilities (*n* = 33) with hardly any literature on youth in fragile, conflict and violence context, *n* = 8; humanitarian settings (n = 6), criminal background (*n* = 3), and ethnic minorities (*n* = 3) (Table 9).

**Table 9:** Evidence types by social population factors

	Social status						Residence	
Studies	FCV	Disability	Crimin.	Disadv.	Human.	Ethnic.	Rural	Urban
Experimental	6	14	2	77	5	1	36	73
Nonexperimental Matching	2	11	0	46	1	2	30	46
Other regression	0	0	0	13	0	0	7	11
Systematic reviews	0	8	1	4	0	0	3	5
Total	8	33	3	140	6	3	76	135

Abbreviations: Crimin., criminal background; Disadv., disadvantaged background (low‐income families or low education); Ethnic., ethnic minority; FCV, fragility, conflict and violence; Human., humanitarian settings.

There were variations in the types of evidence by population as well as country income status. Experimental evidence was concentrated around youth in urban locations (*n* = 73) and those from disadvantaged backgrounds (*n* = 77) (Supporting Information Annex 8: Table 23). There were no systematic reviews (zero) capturing youth employment interventions in fragile, conflict and violent or humanitarian settings, or among ethnic minorities (Table 9). In terms of regions of the world by income status, there were no studies (zero) about employment interventions in youth with disability, criminal background, humanitarian setting or ethnic minority in low‐income countries. The only three studies about youth with criminal background were all in high‐income countries (Table 10).

**Table 10:** Evidence about regions of the world by social population factors

	Social status					
Studies	FCV	Disability	Criminal background	Disadvantaged background	Humanitarian setting	Ethnic minority
High Income	2	30	3	62	2	3
Upper Middle Income	2	2	0	44	0	0
Upper Low Income	0	2	0	24	0	0
Low Income	4	0	0	14	4	0
Total	8	34	3	144	6	3

### 4.3 Intervention categories

The five intervention sub‐categories are support to employment; decent work policies; training; information and finance and entrepreneurship. Training (in full training, up‐skilling and retraining or reskilling) was by far the most researched area (*n* = 283). This was followed by support into employment (*n* = 182). Together these two interventions are assessed in over 75% of the included studies. There are far fewer studies about the effect of information services, decent work policies, entrepreneurship promotion and financing on youth employment (Figure 6). The Figure 7 provides detailed coverage of intervention sub‐categories for the ‘training, upskilling and re‐training intervention category’ while Figure 8 shows intervention sub‐categories for the ‘support to employment’ intervention category.

Under the training and skilling category, most of the evidence was about education, technical and vocational training (TVET), *n* = 193, followed by the life skills training (*n* = 105) and internship and apprenticeship (*n* = 81) intervention sub‐categories. There are few studies regarding Prior Learning Assessment and Recognition (PLAR), *n* = 1, training of trainers and teachers (*n* = 2), and training centre accreditation and certification (*n* = 1) (Figure 7).

In support to employment intervention category, employee mobility and placement (*n* = 77) and mentoring (*n* = 67) were the leading intervention sub‐categories whilst career guidance (*n* = 46) and wage subsidies (*n* = 51) studies had little difference. There was an obvious evidence gap in the area of public works programmes (*n* = 8) (Figure 8).

#### 4.3.1 Multi‐component interventions

A total of 148 (37.%) out of 399 studies included in the EGM combined multiple categories of interventions. Of the 148 studies that combined at least two interventions sub‐categories, majority (68.2%) combined the ‘training, up‐skilling and re‐training/skilling’ and, the ‘support to employment’ intervention categories. Further examination of (Table 11), shows that the intervention sub‐category of the ‘training, up‐skilling and re‐training/skilling’ and, the support to employment are mainly evident in the combinations of most of the other intervention categories. However, the ‘information’ intervention category was the least common in the different combinations of interventions.

**Table 11:** Multi‐component youth employment interventions

Multi‐component Intervention	Studies (number)	Percent (%)
Training, up‐skilling and re‐training/skilling + Support to employment	101	68.2
Training, up‐skilling and re‐training/skilling + Entrepreneurship promotion and financing	9	6.1
Training, up‐skilling and re‐training/skilling + Support to employment + Decent work Policies	6	4.1
Training, up‐skilling and re‐training/skilling + Support to employment + Entrepreneurship promotion and financing	5	3.4
Training, up‐skilling and re‐training/skilling + Support to employment + Decent work Policies + Information	4	2.7
Training, up‐skilling and re‐training/skilling + Support to employment + Entrepreneurship promotion and financing	4	2.7
Training, up‐skilling and re‐training/skilling + Entrepreneurship promotion and financing	4	2.7
Support to employment + Decent work Policies	4	2.7
Training, up‐skilling and re‐training/skilling + Information	3	2.0
Training, up‐skilling and re‐training/skilling + Decent work Policies	2	1.4
Training, up‐skilling and re‐training/skilling + Support to employment + Information + Entrepreneurship promotion and financing	2	1.4
Training, up‐skilling and re‐training/skilling + Decent work Policies + Entrepreneurship promotion and financing	2	1.4
Training, up‐skilling and re‐training/skilling + Support to employment + Decent work Policies + Entrepreneurship promotion and financing	1	0.7
Support to employment + Decent work Policies	1	0.7
Total (studies that combined interventions)	148 (37.1%)	100.0
TOTAL (all studies included in the EGM)	399	

### 4.4 Outcome categories

There are five outcome domains reported in the map: education and skills, entrepreneurship, employment, welfare and economic. Employment is the most reported outcome (*n* = 345) followed by welfare (*n* = 121) and, education and skills (*n* = 97) (Figure 9).

Among the outcome sub‐categories for employment outcomes, evidence is concentrated on employment status (including employment duration (*n* = 322), earnings and salary (*n* = 197), and hours worked (*n* = 89) (Figure 10). Within the outcome category of education and skills, education completion and qualification, *n* = 36 (Figure 14); and business creation, *n* = 54, under entrepreneurship category (Supporting Information Annex 8: Figure 15). For the outcome category welfare, the three most studies outcomes are citizenship, value and social behaviour, *n* = 62, inclusion and empowerment, *n* = 59, and economic outcomes (not including earnings), *n* = 54. The most reported economic outcomes are cost–benefit, *n* = 41 (Supporting Information Annex 8: Figure 16).

The clear evidence gaps in the employment outcome domain literature were about: vacancies, actively seeking employment, employment expectation and appropriate employment (Figure 10). Multiplier and spill over effects, digital skills and job creation sub‐categories were the obvious gaps in the evidence around economic, education and skills and entrepreneurship major outcome domains (Supporting Information Annex 8: Figures 13–15).

### 4.5 Aggregate map of evidence gaps

Table 12 is the aggregate map that shows the number of studies in each intervention and outcome category.

**Table 12:** Aggregate map of evidence gaps

	Outcomes				
Interventions	Economic	Education and skills	Entrepreneurship	Employment	Welfare
Training, up‐skilling and re‐training/skilling	36	82	54	239	98
Support to employment	26	45	24	165	54
Decent work Policies	3	5	4	59	6
Information	0	2	4	6	3
Entrepreneurship promotion and financing	8	11	22	31	25

Well‐evidenced areas with more than 75 studies are the employment, welfare, as well as education and skills outcomes of training interventions. This level also is employment outcomes of support to employment interventions.

Moderately evidenced areas, with between 25 and 75 studies, are largely identified for support to employment interventions across a broader range of outcomes, except employment. Training interventions also have moderate level of evidence for economic and entrepreneurship outcomes. Similarly, entrepreneurship promotion and financing interventions had moderate level of evidence for employment and welfare outcomes. The evidence base is weak (fewer than 25 studies) especially for information intervention category across all outcomes.

### 4.6 Risk of bias in included reviews

The results from the quality assessment of systematic reviews using the AMSTAR 2 tool and, impact evaluation studies basing on the ‘Quality assessment of Impact Evaluations’ tool are presented below. These study quality assessment tools are detailed earlier in (Section 3.7). A detailed risk of bias or study quality table containing a list of 399 studies is provided in Supporting Information Annex 7.

Overall, most systematic reviews (71.4% of 21) were of medium and high quality while 28.6% were graded as low quality by AMSTAR score (Table 13). Across the intervention sub‐categories and outcomes categories, evidence quality was mainly rated as ‘medium/moderate and high quality’ (Table 14). The reviews with a low quality rating had at least one critical flaw (Table 15). For instance, all the reviews rated as low quality did not explicitly state if the review methods were established before the conduct of the review and did not also justify any significant deviations from the protocol. The inability of review authors accounting for risk of bias (RoB) in individual studies when interpreting/discussing the results of the review, was the second most dominant critical flaw (Table 15). A detailed report of RoB assessment for systematic reviews is provided in Supporting Information Annex 6. The conduct of systematic therefore calls for improvements in a number areas such as proper design of methods and their publication for transparency, undertaking risk of bias analysis and use of comprehensive literature search strategies.

**Table 13:** Quality of evidence/risk of bias of included studies

Studies	Low quality (%)	Medium and high quality (%)
Systematic reviews	6 (28.6)	15 (71.4)
Impact evaluations	278 (73.4)	101 (26,6)
Experimental	103 (58.2)	74 (41.8)
Nonexperimental matching	144 (86.2)	23 (13.7)
Other regression	31 (88.6)	4 (11.4)
Total	284 (71.2%)	116 (29.1%)

*Note*: Total percentage adds to more than 100, as an impact evaluation study may be coded for more than one design.

**Table 14:** Quality of evidence by intervention sub‐categories and outcome categories

	Impact evaluations		Systematic reviews	
	Low quality primary study	Medium and high quality primary study	Low quality systematic review	Medium and high quality systematic review
*Intervention sub‐categories*				
Training, up‐skilling and re‐training/skilling	187	77	6	13
Support to employment	121	48	3	10
Decent work Policies	51	6	0	3
Information	1	4	1	2
Entrepreneurship promotion and financing	19	20	1	3
*Outcome categories*				
Economic	29	12	0	0
Education and skills	63	28	1	5
Entrepreneurship	31	25	2	5
Employment	249	84	2	10
Welfare	64	45	4	8

**Table 15:** Critical flaws in systematic reviews rated as low quality

	Systematic review (authors)						
AMSTAR 2 Checklist	Cobb (2009)	Grimm (2015)	Catalano (2019)	Hanif (2017)	Ke (2018)	Jennings (2014)	Critical flaws
2*. Did the report of the review contain an explicit statement that the review methods were established before the conduct of the review and did the report justify any significant deviations from the protocol?	*no*	*no*	*no*	*no*	*no*	*no*	**6**
7*. Did the review authors provide a list of excluded studies and justify the exclusions?	*no*						**1**
4*. Did the review authors use a comprehensive literature search strategy?			*no*	*no*			**2**
13*. Did the review authors account for RoB in individual studies when interpreting/discussing the results of the review?	*no*	*no*	*no*		*no*	*no*	**5**
15*. If they performed quantitative synthesis did the review authors carry out an adequate investigation of publication bias (small study bias) and discuss its likely impact on the results of the review?	*no*						**1**

Looking at impact evaluations, at least 73.4% of these category studies were of low confidence quality rating (Table 13). In terms of Intervention sub‐categories and Outcomes categories, the bulk of the evidence was of low quality impact evaluations across the board (Table 14). These studies obtained a low‐quality rating on at least one of the following three items: (1) high attrition rate; (2) design (potential confounders not taken into account and; (3) weak baseline balance performance. However, attrition bias and low baseline balance rating were the two main reasons for the low‐quality rating of impact evaluations.

Results of attrition assessment presented in Figure 11, show that most (70.4%) impact evaluations scored a low rating because either attrition was not reported or the reported measures fell outside What Works Clearinghouse (WWC) Standards acceptable combined levels. Ex‐post factor studies were not rated on this attrition parameter.

Regarding the ‘baseline balance parameter’,^5^ still a big number of studies (43.4%) scored a low rating because they did not have baseline balance data or were reported with significant differences on more than five measures (Figure 12). It means that most studies with treatment and comparison groups did not have the same average characteristics of participants at baseline. Such imbalances in participants' characteristics such as in the levels of education often affect the impact of the interventions.

The ‘before versus after’ studies were not rated on this parameter as it is not applicable. The RCTs were automatically rated high on this parameter.

### 4.7 Discussion

#### 4.7.1 Overview of the map

This map primarily targets policy makers and youth development practitioners as well as researchers who require evidence to inform policy and implementation of youth employment interventions. On the one hand and to the governments and international partners, this map is a useful guide to identifying existing evidence related to youth employment interventions under consideration. Researchers and research commissioners are able to identify areas of available evidence and commission syntheses where there are gaps in knowledge, they would commission new impact evaluations as appropriate.

#### 4.7.2 Principal findings

The EGM of global literature on youth employment interventions over two decades was conducted. The EGM contains 399 eligible studies mostly (59%) from high‐income countries in Europe, Central Asia and North America. This suggested that country income persisted in research productivity, yet it is in Africa that youth unemployment will likely be more impactful in decades to come. Moreover, these low and middle‐income regions have the biggest population of youth NEET. However, the reasons for this situation are beyond the scope of this EGM. It's therefore an evidence gap for research investment. Naturally, this trend persisted among the lead authors with hardly any from Africa having two or more publications. Majority of the studies were experimental impact evaluations with few systematic reviews and interventions were located in firms or youth training centres.

It was observed that the bulk of evidence on interventions studied was from low quality impact evaluations (71.2%). Fortunately, most systematic reviews (71.4% of 21) were of medium and high quality. There were two key reasons for failings of impact evolutions. First, they had very high levels of attrition by study participants or attrition data were not reported. The second, was the large differences in the baseline characteristics between treatment groups and control groups of participants. In the case of systematic reviews, there were critical flaws characterised by uncertainties about whether such reviews had protocols before their conduct and finally, risk of bias assessment for included studies was not carried in the systematic reviews with low quality rating. This finding serves to alert researchers, practitioners and policy makers that more rigorous work is needed to inform youth employment interventions.

Overall, evidence was generally scarce in specific areas: Social impact bonds, crowd funding, micro‐franchising, overseas employment, public works, information access and accountability systems. Additional gaps were in specific population sub‐groups, interventions and outcome categories that research commissioners may consider in their agendas. These findings could possibly be a function of context‐specific capacities to conduct the research or priorities.

Older youth, youth in fragility, conflict and violence contexts, or humanitarian settings, or ethnic minorities or those with criminal backgrounds were least studied. In fact, there were no systematic reviews at all capturing youth employment interventions among these unique population groups or even impact evaluations in low‐income countries. The underrepresentation of evidence availability for youth interventions in such contexts and settings could undermine evidence‐based policymaking.

It was observed that three main intervention sub‐categories were underrepresented; information services, decent work policies and entrepreneurship promotion and financing. Despite generally available literature at main intervention category level, there were gaps at intervention sub‐categories. For example, training of trainers, and training centre accreditation and certification under training and skilling main categories. It's also finding that public works was under‐researched or simply governments engaged more the private sector. Generally, there was a clear evidence gap in the economic outcome domain literature that was really about costs and, multiplier and spill over effects. Other areas of concern were digital skills and job creation sub‐categories under education and skills and entrepreneurship major outcome domains.

Specific areas were saturated with evidence. The intervention category with most evidence is training. Within the training intervention category, the subcategory of ‘education, technical and vocational training (TVET)’, has the largest prevalence of evidence. These include literature about youth below 25 years from high‐income countries and with disadvantaged background, that is, from low‐income families or with low education. Training and skilling and, support to employment were well endowed with impact evaluations. Employee mentoring, mobility and placements were common studies in the support to employment intervention whilst life skills, education, technical and vocational training were predominant in the skilling and training literature. Literature on outcomes was concentrated around employment and welfare‐related outcomes, such as earnings, hours worked, inclusion and empowerment, family health and education and, citizenship values.

The prominence of multi‐component interventions has been observed in that many studies combined at least two youth employment interventions. Two intervention sub‐categories namely; ‘training, up‐skilling and re‐training/skilling’ and, ‘support to employment’, stood out as the most common combination of interventions. Moreover, the same two intervention sub‐categories still populate the several other combinations of interventions. While this finding could be an indication that blended interventions offer better outcomes, this remains an area with a research gap. This EGM therefore provides a basis for the establishment of a typology of youth employment interventions.

### 4.8 Potential biases in the mapping process

#### 4.8.1 Strengths

The map provides more recent information. This is the most up to date EGM on youth employment interventions. It contains a total of 399 impact evaluations and systematic reviews that were published or made available between the year 2000 and 2019. In addition, at least one in five of these studies were published after the last major two evidence syntheses, from 2016 to 2019. None of the other two existing EGMs on youth employment interventions included studies published after 2015. Youth and transferable skills EGM by 3ie included 104 studies while the Youth Employment EGM by ILO was based a systematic review by Campbell Collaboration that reviewed 113 reports.

Further, there were methodological strengths including a participatory approach in protocol development involving the whole synthesis project team with African leads, a broad search strategy of 20 data bases with citation tracking and a broader age range of the definition of youth. All studies identified at different stages of study search were screened and coded by pairs of reviewers all of which improved the trust in this evidence synthesis. Not least, this map identifies key evidence gaps for future prioritisation.

#### 4.8.2 Limitations

Due to the COVID‐19 lockdown, reviewers were unable to conduct face‐to‐face stakeholder engagements in Kampala, Uganda as stated in the study protocol. Keeping in touch with Mastercard Foundation policy leads and the literature were informative in arriving at reasonable priority list of the interventions and outcomes for this EGM.

Another key weakness in this map is that the bulk of the impact evaluations were of low quality. Attrition bias and low baseline balance measures were the two main reasons for the low quality rating of impact evaluations which generally account for a huge bulk (378 of 399) of studies that are included in the EGM. Nonetheless, as EGMs do not intend to communicate evidence of effects of interventions, this finding serves to alert researchers, practitioners and policy makers that more rigorous work is needed to inform youth employment interventions. Researchers need to improve conduct and reporting of impact evaluations. Improvements may start within postgraduate programs funded by Mastercard Foundation by linking postgraduate students to impact evaluations of youth employment interventions.

Finally, this information is time bound not earlier than 2000 for which we could have missed important information. However, the purpose of this EGM was to build on existing syntheses that captured the earlier work of youth employment interventions globally.

### 4.9 Conclusions

This report provides a summary of findings of the Youth Employment EGM that focuses on interventions aimed at increasing youth employment in any country of the world. The map was funded by Mastercard Foundation and Youth Futures Foundation and, developed by The African Centre for Systematic Reviews and Knowledge Translation—Makerere University of College of Health Sciences in partnership with the Campbell Collaboration Secretariat. The map is an effectiveness map. Hence included studies are impact evaluations of interventions to improve youth labour market outcomes or systematic reviews of such studies. In total 399 studies are included (21 systematic reviews and 378 impact evaluations).

The map identifies trends in evidence notably the following:

- Most evidence is from North America and Europe and as such, high‐income countries have the largest share of evidence.
- The most common study designs are experimental.
- Most of the evidence is of low quality. This finding serves to alert researchers, practitioners and policy makers that more rigorous work is needed to inform youth employment interventions.
- Blending of interventions is practiced. While this could be an indication blended intervention could be offering better outcomes, this remains an area with a research gap.

Inspecting the number of studies in each cell of the map shows clusters of evidence and gaps, as follows:

- Well‐evidenced areas, with more than 75 studies, are the employment, welfare, as well as education and skills outcomes of training interventions. This level also is employment outcomes of support to employment interventions.
- Moderately evidenced areas, with between 25 and 75 studies, are largely identified for support to employment interventions across a broader range of outcomes, except employment. Training interventions also have moderate level of evidence for economic and entrepreneurship outcomes. Similarly, entrepreneurship promotion and financing interventions had moderate level of evidence for employment and welfare outcomes.
- The evidence base is weak (fewer than 25 studies), for information and decent work policies.

The map reveals areas with well‐populated evidence as well as those with gaps. Hence to a great extent the map enhances discoverability of evidence by stakeholders engaged in the promotion of youth employment interventions.

## CONFLICT OF INTERESTS

The authors declare that there are no conflict of interests.

## FUNDING

The funding for the map is from by Mastercard Foundation and Youth Futures Foundation.

## ROLES AND RESPONSIBILITIES

**Project Director was** Dr. Ekwaro Obuku, who has vast experience in management evidence synthesis teams. He provided generation administration to the project team. Ekwaro is the Director of Africa Centre for Systematic Reviews and Knowledge Translation, based at the Makarere University College of Health Sciences (Africa Centre MakCHS), Uganda. He is a coauthor of several systematic reviews and tutors Systematic Reviews at Makerere University. He recently started a course in Evidence Synthesis for Masters Students in Clinical Epidemiology and Biostatistics at Makerere University, Uganda.

**Content and methods expert**: Dr. Howard White is a development economist with a tremendous track record in conducting development evaluations. He has studied labour markets in Africa. Howard played a key role of quality assurance though out the project implementation, in addition to providing content and methods expertise for the project.

**Project Manager** was Mr. Robert Apunyo. He has vast experience in managing research projects involving multidisciplinary teams. He is a Research Fellow at Africa Centre MCHS.

**Methods expert was** Dr. Ashrita Saran. She has vast experience in systematic review methodology and theory‐based synthesis. She was instrumental in the development of the EGM framework and training of screeners and coders of studies.

**Information retrieval was conducted by** Dr. Alison Annet Kinengyere and John Eyres. Alison and John have previously supported various systematic reviews projects.

**Advisors**: Susana Puerto and Drew Gardiner both from ILO provided technical advice throughout the project life.

**Coders**: Ms. Caroline Otike and Mr Thomas Katairo did most of the coding. Caroline and Thomas are Research Fellows at Africa Centre MCHS.

## PLANS FOR UPDATING THE EGM

Youth Futures Foundation and MasterCard Foundation have shown interest in updating the EGM. The Youth Futures Foundation has already availed financial support for updating the EGM with process evaluation in last quarter of 2021. The EGM will be updated on an annual basis.

## DIFFERENCES BETWEEN PROTOCOL AND REVIEW

Stakeholder consultations were not conducted due to the COVID‐19 global lockdown. Meetings and workshops to engage stakeholders were planned to be conducted in Uganda in the last quarter of 2019 with relevant officials in Uganda from government ministries, departments, agencies, private sector agencies, civil society organisations, vocational training institutes, international development agencies as well as academia. However, keeping in touch with Mastercard Foundation policy leads and the literature were informative in arriving at reasonable priority list of the interventions and outcomes for this EGM.

## LINK TO ONLINE INTERACTIVE EGM

https://youthfuturesfoundation.org/wp-content/uploads/2021/09/Evidence-and-Gap-Map.html.

## Supporting information

Supporting information.

## Footnotes

^1^
‘Youth employment EGM’ is the short title adopted from the main title, ‘interventions to increase Youth employment: An evidence and gap map’.

^2^

*Attrition or losses to follow up*: can be a major source of bias in studies, especially if there is differential attrition between the treatment and comparison group (called the control group in the case of RCTs) so that the two may no longer be balanced in pre‐intervention characteristics. The US Institute of Education Sciences What Works Clearing House (WWC) has developed standards for acceptable levels of attrition, in aggregate and the differential, which are applied in this EGM.

^3^
Baseline balance means that the treatment and comparison groups have the same average characteristics at baseline, not only for outcomes but other factors which may affect the impact of the programme such as a prior history of parental alcohol abuse.

^4^

http://www.amstar.ca/Amstar_Checklist.php.

^5^
Baseline balance means that the treatment and comparison groups should have almost similar average characteristics at baseline, not only for outcomes but other factors which may affect the impact of the programme such as a prior history of parental alcohol abuse.
