# Supplementary material for: Interventions to increase youth employment: An evidence and gap map
Source: Campbell Syst Rev. 2022 Feb 15;18(1):e1216. doi: 10.1002/cl2.1216 (PMC8847398; doi:10.1002/cl2.1216)
Supplement: Supplementary file 1 — Supporting information. [file CL2-18-e1216-s001.docx]

# Abbreviations and acronyms

| AMSTAR | : | A Measurement Tool to Assess systematic Reviews |
| --- | --- | --- |
| DiD | : | Difference in difference study design |
| EGM | : | Evidence and Gap Map |
| FCV | : | Fragility, Conflict and Violence |
| ILO | : | International Labour Organisation |
| MakCHS | : | Makerere University College of Health Sciences |
| NEET | : | Not in Education, Employment or Training |
| PICOS | : | Population, Intervention, Comparator, Outcomes and Study design |
| PLAR | : | Prior Learning Assessment and Recognition |
| PSM | : | Propensity Score Matching |
| RCT | : | Randomised Controlled Trial |
| RDD | : | Regression Discontinuity Design |
| SDG | : | Sustainable Development Goals |
| SME | : | Small and Medium sized Enterprise finance |
| TVET | : | Education, technical and vocational training |
| UK | : | United Kingdom |
| UNDP | : | United Nations Development Programme |
| USA | : | United States of America |

## Annexes

**Annex 1: Definitions of Interventions**

| **Intervention category** | **Intervention sub-category** |
| --- | --- |
| **Training, up-skilling and retraining / re-skilling:** Training is teaching, or development in oneself or others, any skills and knowledge or fitness that relate to specific useful competencies. Up-skilling-the process of learning new skills or of teaching workers new skills (Cambridge) Retraining is a practice employer may require for their workers to make them learn new skills especially to avoid stagnant workforce. | **Prior Learning Assessment and Recognition (PLAR):** Prior learning assessment and recognition defines processes that allow individuals to identify, document, have assessed and gain recognition for their prior learning. The learning may be formal, informal, non-formal, or experiential. The context of the learning is not key to the process as the focus is on the learning. PLAR processes can be undertaken for several purposes, including self-knowledge, credit or advanced standing at an academic institution, for employment, licensure, career planning or recruitment. Tools such as challenge exams, demonstrations, structured interviews, simulations and portfolios can be used alone or in combination, for experiential learning and competency assessment in such instances (CAPLA,^[[1]](#footnote-1)^ 2019). |
|  | **Technical and vocational training (TVET):** Technical and vocational education and training’ (TVET) is understood as comprising education, training and skills development relating to a wide range of occupational fields, production, services and livelihoods. TVET, as part of lifelong learning, can take place at secondary, post-secondary and tertiary levels and includes work-based learning and continuing training and professional development, which may lead to qualifications. TVET also includes a wide range of skills development opportunities attuned to national and local contexts. Learning to learn, the development of literacy and numeracy skills, transversal skills and citizenship skills are integral components of TVET (UNESCO, 2019a). Stipends during training should be coded here. |
|  | **Internship:**  These are short-term periods of temporary work experience, typically lasting for a few weeks or months mainly to gain on-the-job experience of working in a particular role, organization or industry sector *(*Finch, (2018) & International Labour Organization, (2012*).*  **Apprenticeship**: An apprenticeship is a formal employment program that provides training to do a specific job. Unlike internships, apprenticeships employ people who already know which career path they wish to follow. If they join a program, they will sign a contract with the employer and learn specific skills during their apprenticeship. Training last longer – a few years (Finch, 2018). |
|  | **Training centre accreditation and certification:**  **Accreditation** is the process by which a (non) governmental or private body evaluates the quality of a higher education institution as a whole or of a specific educational programme in order to formally recognize it as having met certain predetermined minimal criteria or standards. Types of accreditation include Institutional, Regional and Specialized accreditation (Vlăsceanu *et al.,* 2004).  **Certification** is the process by which an agency or an association acknowledges the achievement of established quality standards and usually grants certain privileges or written assurance to the target individual – student (Vlăsceanu *et al.,* 2004). |
|  | **Training of trainers and teachers:** Training of Trainers (TOT) is a high-level professional learning process for qualified trainers who provide training and capacity-building assistance (Program Success Centre for Sexual & Reproductive Health, 2019) e.g., on youth employment skills. An example is the TOTs on youth empowerment for advocacy and campaigning on youth unemployment. |
|  | **Business skills training:** This involves training in Financial management, Marketing, sales and customer service, Leadership, Project management and planning, Delegation and time management, Problem solving and, Networking (Small Business Development Corporation, 2019). |
|  | **Life skills training:** Life skills training is intended to strengthen trainees’ self-esteem and work habits by equipping them with a wide set of behaviours, attitudes, and personal qualities so that they develop the ability to navigate their environment, work well with others, perform well, and achieve set goals. Examples life skills or soft skills include social skills, communication skills, problem solving skills, decision making skills and elf-control (Lippman et al., 2015). Life skills training is deferent from vocational training in that the former is meant to address the technical training needs of local employers (Ibarraran, et al., 2012). |
| Support to employment | **Employee Mentoring (Work integrated learning, On-job-training (OJT):** The relevant/applicable meaning of OJT is: a Work Based Training or In-service Training or On-Site Training (UNESCO, 2019b) for employees. It excludes apprentices and interns. |
|  | **Career offices/advisory services/career days:** Career centres/offices provide information or assist individuals in making and implementing informed educational and occupational choices. A career guidance and counselling program develops an individual's competencies in self-knowledge, educational and occupational exploration and career planning (U.S.A. Department of Education, 2019). |
|  | **Programme for overseas employment:** Overseas employment refers to employment of a migrant worker in a foreign country, where workers often sign agreements with their employers for a specified period of time. Overseas employment programmes are mainly set up and run government agencies of developing countries in attempt to address high rates of unemployment at home buy exporting labour to foreign countries. Philippines and Jamaica provide some of the most elaborate examples of overseas employment programmes. |
|  | **Public work programs:** Public works in general means construction, alteration, demolition, installation, or repair work done under contract and paid in whole or in part out of public funds (USA. State of California Department of Industrial Relations 2020). Primary beneficiaries of public works programs are the hard-to-employ and socially vulnerable groups who, by being engaged in public works, are offered a chance to earn regular income, leastwise in a short run, and maintain and improve their skills (EU, 2017). |
|  | **Support to employee mobility and placements:** Including financial assistance for job search, job search assistance.  Geographical mobility (employee relocation); Occupational mobility (employee movement into and out of different job species), Organizational mobility (movement of individuals up and down or laterally amongst organization) (Scism, 1974).  A job placement is when an unemployed person, is put into a job that is suitable to their skills and interests, usually for a temporary period (Longman, 2019)**.** |
|  | **Wage subsidies:** These are transfers on non-wage employment costs. Their main goal is to provide incentives for employers to hire members of the target group such as unemployed youth (Bordos *et al.,* 2015). Governments are the major providers of wage subsidies. These could be direct money transfers to firms or tax deductions for firms employing youth. |
| Decent work Policies (Policy reforms influencing professional practices & shaping policy for data sharing).  Decent work sums up the aspirations of people in their working lives. It involves opportunities for work that is productive and delivers fair income, security in the workplace and social protection for families, better prospects for personal development and social integration (International Labour Organization, 2019). | **Labour standards:** These are legal instruments drawn up by the ILO's constituents (governments, employers and workers) and setting out basic principles and rights at work. They are either Conventions (or Protocols), which are legally binding international treaties that may be ratified by member states, or Recommendations, which serve as non-binding guidelines. In many cases, a Convention lays down the basic principles to be implemented by ratifying countries, while a related Recommendation supplements the Convention by providing more detailed guidelines on how it could be applied. Recommendations can also be autonomous, i.e., not linked to a Convention (International Labour Organization, 2019). |
|  | **Social protection and social security:** Social protection includes benefits for unemployment, employment injury etc. Social protection systems address all policy areas by a mix of contributory schemes (***social insurance***) and non-contributory tax-financed benefits, including ***social assistance*** (International Labour Organization, 2017). **Social security** involves access to health care and income security, particularly in cases of unemployment, work injury, maternity or loss of a main income earner (International Labour Organization, 2020). |
|  | **Accountability systems:** Accountability is the obligation to demonstrate that policy has been designed and implemented effectively, and to report on results in a timely and accurate manner. The employment policy accountability system generally comprises seven elements: (a) national level priorities with goals and targets, (b) work plans with specific targets and measures, (c) a budget allocation, (d) a performance framework with delivery plan and indicators of outcome, (e) statistics and reporting, (f) monitoring and evaluation, and (g) adjustment and improvement of employment policy (International Labour Organization, n.d.). |
|  |  |
| Information | **Labour market information:** Labour market information covers the principal elements of the labour market and its operations. For instance, labour market information on current demand, broken down by occupation and skills level, including early identification of sectoral trends and of changes in technology and occupations leading to changing skills composition (International Labour Organization, 2011) |
|  | **Digital services and SMS coaching:** Digital government services (also called e-government) are defined as service delivery within government — as well as between government and the public — using information and communication technologies. Common digital services range from filling tax returns to renewing a driver’s license to applying for a pet license. Nearly any government form or service can be offered digitally (Granicus, 2019). |
|  | **Social media campaigns and awareness campaigns:** Social media campaigns are marketing campaigns on platforms like Facebook, Twitter, Linkedin or Instagram. They have the potential to engage followers, boost brand awareness e.g., on matters of youth employment (Heavey, 2017). |
|  | **Access to services and markets (value chains):** Market access in services is more complex than market access for trade in goods. For trade in goods, market access is about reducing mainly border measures such as tariffs that are imposed on goods as they enter a market. For trade in services, market access is about reducing government policy interventions, which are less visible and may be applied after a service supplier has entered the market. These measures take the form of government regulations that are usually aimed at domestic policy objectives rather than trade policy objectives (McGuire, 2002). |
|  | **Value chain development:** The ILO Value Chain Development approach looks at market dynamics and relationships between the different actors in the chain with the objective of strengthening the whole market system - enterprises, business relationships, financial networks, supporting functions, rules and norms, and the business environment – in a way that ensures greater benefits for the poor from economic growth and development (Nutz & Sievers, 2015). |
| Entrepreneurship promotion and Financing | **Small and medium sized Enterprise finance (SME):** Improving SMEs’ access to finance and finding innovative solutions to unlock sources of capital (World Bank 2020). This may include lending operations or organizations/agencies and countries setting a side specific funding for SMEs. |
|  |  |
|  |  |
|  | **Microfinance (to individuals):** Microfinance, also called microcredit​, is a type of banking service provided to unemployed or low-income individuals or groups who otherwise would have no other access to financial services (Investopedia, 2019b). |
|  | **Social impact bonds:** A social impact bond (SIB) is a contract with the public sector or governing authority, whereby it pays for better social outcomes in certain areas, such as youth employment, and passes on the part of the savings achieved to investors (Investopedia, 2019). Implementers are paid for results (youth employed) not activities. |
|  | **Crowd funding**: Crowdfunding is the use of small amounts of capital from a large number of individuals to finance a new business venture. Crowdfunding makes use of the easy accessibility of vast networks of people through social media and crowdfunding websites to bring investors and entrepreneurs together, with the potential to increase entrepreneurship by expanding the pool of investors beyond the traditional circle of owners, relatives and venture capitalists (Investopedia, 2019a). |
|  | **Loan guarantees:** A guaranteed loan is a loan that a third party (e.g., government agency) guarantees – or assumes the debt obligation for – in the event that the borrower defaults. Guaranteed loan agreements may be made when borrowers such as unemployed youth are considered unattractive candidates for regular bank loans. It is a way for people (unemployed youth) who need financial assistance to secure funds when they otherwise may not qualify to acquire them (Kagan, 2019). |
|  | **Grants:** These are funds that do not have to be paid back by the recipients, under most conditions. Grants meant for youth employment can include; entrepreneurship grants, education grants and research money. Some **grants** have waiting periods, called lock-up or vesting periods, before the grantee can take full ownership of the financial reward (Chen, 2018). |
|  | **Self-financing groups:** Self-Help or financing groups are small groups, often mostly or only women, who save a certain amount of money each week or each month and have group meetings weekly or monthly. Out of these collective savings they issue each other loans. Groups decide democratically how much interest they can charge, what the penalties will be for not coming to the meetings or showing up late to the meetings, and what amount they will save each week or month. They decide what the requirements are for dropping out of the group and the requirements for new members joining (Flynn, 2013). |
|  | **Micro franchising**: Micro-franchising is a business model that applies traditional franchising to very small businesses. It is a systemized approach to replicating micro-enterprises like drive-in coffee kiosks, mall products and services, food stands, and just about any other type of business that sells low-cost products or services, primarily in high traffic areas (Forbes, 2019). |

Annex 2: Definitions of *O*utcomes

| **Economic outcomes:**  These include cost, cost benefits, cost effectiveness and, multiplier and spill-over effects. |
| --- |
| **Cost:** Costs are the necessary expenditures that have to be paid or given up in order to get something (Business Dictionary 2020) or for implementation of a youth employment intervention. For example, cost/monetary value of wage subsidy (Betcherman, et al 2010). |
| **Cost Benefits:** Cost Benefit Analysis is an economic evaluation technique that compares the cost of the intervention with the benefit incurred, where the benefit is measure by monetary unit. Here, both costs and consequences are measured in monetary unit. Net Benefit= Benefits – Costs |
| **Cost effectiveness:** Cost Effectiveness Analysis (CEA) is an economic evaluation technique that compares ‘cost per consequence’ of two or more interventions, where the consequences are measured by ‘natural’ units (life years gained, saved years of life). CEA focuses on non-monetary outcomes. Cost effectiveness ratio (CER) = Cost of Intervention/Effect of Intervention (Cellini& Kee, 2015) |
| **Multiplier and spillover effects:** Effects not directly in the programme e.g., youth spending earnings to improve local commerce and displacement of employment. |
| **Education and skills outcomes:**  Educational outcomes broadly refer to notable achievements in education, satisfaction with quality of education programs; transformative experiences and, career readiness and performance (Fleming, 2015). Specific outcomes defined below include (education completion, education quality and access to education).  The ‘Skills set’ of outcomes in this context include attained technical and transferable skills. Technical skills refer to those got after undergoing a training and such skills are meant to address the technical training needs of local employers (Ibarraran, et al., 2012). However, transferable skills include life and social skills such as networking skills, are meant help the individual navigate their environment. |
| **Education completion:** Percentage of a cohort of children or young people aged 3-5 years above the intended age for the last grade of each level of education who have completed that grade. The intended age for the last grade of each level of education is the age at which pupils would enter the grade if they had started school at the official primary entrance age, had studied full-time and had progressed without repeating or skipping a grade. For example, if the official age of entry into primary education is 6 years, and if primary education has 6 grades, the intended age for the last grade of primary education is 11 years. In this case, 14-16 years (11 + 3 = 14 and 11 + 5 = 16) would be the reference age group for calculation of the primary completion rate (UNESCO, 2019c). |
| **Access to education:** Access to education includes: on-schedule enrolment and progression at an appropriate age, regular attendance, learning consistent with national achievement norms, a learning environment that is safe enough to allow learning to take place, and opportunities to learn that are equitably distributed (Lewin, 2015). |
| **Education quality:** A good quality education is one that provides all learners with capabilities they require to become economically productive, develop sustainable livelihoods, contribute to peaceful and democratic societies and enhance individual well-being. The learning outcomes that are required vary according to context but at the end of the basic education cycle must include threshold levels of literacy and numeracy, basic scientific knowledge and life skills including awareness and prevention of disease. Capacity development to improve the quality of teachers and other education stakeholders is crucial throughout this process (vvob, 2019). |
| **Technical skills and vocational training (TVET)**: Technical and vocational education and training’ (TVET) is understood as comprising education, training and skills development relating to a wide range of occupational fields, production, services and livelihoods. TVET, as part of lifelong learning, can take place at secondary, post-secondary and tertiary levels and includes work-based learning and continuing training and professional development, which may lead to qualifications. TVET also includes a wide range of skills development opportunities attuned to national and local contexts. Learning to learn, the development of literacy and numeracy skills, transversal skills and citizenship skills are integral components of TVET (UNESCO, 2019d). |
| **Digital skills:** Digital skills are defined as a range of abilities to use digital devices, communication applications, and networks to access and manage information. They enable people to create and share digital content, communicate and collaborate, and solve problems for effective and creative self-fulfillment in life, learning, work, and social activities at large (UNESCO 2019e). |
| **Transferable skills (including life skills e.g., networking, negotiation):** A transferable skill is a skill learned one another context (in the case of research) and used in another (for example future employment whether in research, business etc.). They can serve as a bridge from study to or from one career to another as they enable the subject and related skills to be applied and developed effectively in different work environments. They include skills such as communication skills and organizational skills (Organisation for Economic Co-operation and Development (OECD), 2012). |
| **Entrepreneurship outcomes:**  These broadly refer to business creation, business performance, job creation as well as access to financial services. |
| **Access to financial services:** The availability to a given person of affordable and appropriate financial services e.g., to youth investors. Access is often seen as the goal of financial inclusion (Centre for Financial Inclusion, 2019). |
| **Business creation:** Creating a new business is a process which starts with an idea and involves many stages before launching a new product or process on the market (Innovaccess - Intellectual Property Portal, 2019) |
| **Business performance:** Including profits, sales, number of employees and jobs created, capital and investment, business creation and business survival (Kluve, *et al* 2014). |
| **Job creation:** The process by which the number of jobs in an economy increases. Job creation often refers to government policies intended to reduce unemployment. Job creation programs may take a variety of forms. For example, a government may lower taxes and reduce regulation to make hiring less expensive. On the other hand, a government may hire workers itself, for example, to build a road (Farlex Financial Dictionary, 2012) |
| **Employment outcomes:**  These refer to the following: Employment status, seeking employment, vacancies, employment expectation, employment consistent with education/training, Hours worked, job quality and earnings. |
| **Vacancies:** Number of unoccupied positions for a job. |
| **Actively seeking employment:** This includes actions undertaken by a youth during participation in a youth employment programme, in attempt to find employment: job applications submitted; attendance of job fairs, reviewing job advertisements and, attending job interviews etc. (Azevedo, et al., 2013). |
| **Employment expectation:** This may entail expectations of improved future employment conditions (Acevedo, 2017), likelihood of being offered job and, likelihood of being deployed if offered a job (Beam, 2016). |
| **Employment status (including duration):** **Employment status** is the status of a worker in a company on the basis of the contract of work or duration of work done. A worker may be a full-time employee, part-time employee, or an employee on a casual basis or unemployed. S/he could be employed temporarily for a specific project only, or on a permanent basis. Part-time wage labor could combine with part-time self-employment. The worker could be employed also as an apprentice (US Legal, 2019).  **Employment duration:** Longevity; duration of service or employment. Often used to indicate how long an employee has worked at a company or an individual has belonged to an organization (Business Directory, 2019). |
| **Employment consistent with education/training:** This refers to employment which is related to the skill learned or owned by an employee (Ahmed et al., 2014). Skills match with tasks or worker is not under/over qualified for the job (Abebe et al., 2017). |
| **Hours worked:** In general, ‘hours worked’ includes all time an employee must be on duty, or on the employer's premises or at any other prescribed place of work. Also included is any additional time the employee is allowed to work, (i.e., overtime, hourly wage, double time) (USA. Department of Labour, 2019). |
| **Job quality:** Job quality complements measures of job quantity to provide an assessment of employment strategy. Job quality is a multidimensional concept where different policy agenda and disciplines emphasize different dimensions. Job quality is measured at the level of the job. It includes job features captured from an objective perspective, which can be observed and are related to meeting people’s needs from work. It is made up of all the characteristics of work and employment that have been proven to have a causal relationship with health and well-being. Positive and negative features of the jobs are included. These indicators reflect the job resources (physical, psychological, social or organizational aspects) and job demands, or the processes that influence them (Eurofund, 2019). Job quality includes informal vs informal jobs. |
| **Earnings:** money obtained in return for labour or services. Including reported earnings and income, household income, consumption and salary and/or wage (Kluve *et al.,* 2014). |
| **Welfare outcomes:**  These include the following: Criminal and delinquent behavior; citizenship; values and social behavior, family health and education and; Inclusion and empowerment (social network. |
| **Criminal and delinquent behavior (anti-social behavior):** Crime can involve violence, sex or drugs but also discrimination, road rage and burglary. Crime is any behaviour and any act, activity or event that is punishable by law (Government of Netherlands, 2019). |
| **Citizenship, values and social behavior:** Personal values are reliable cross-situational predictors of attitudes and behaviour. Personal values are individual conceptions of the desirable that guide behavior in little things like donating to charity or spending time with the family and in life-defining decisions (Ponizovskiy *et al.,* 2019). Social behaviour is such things as taking part in community activities. |
| **Economic outcomes (except earnings):** These include assets, savings and business profits of firms etc. (Blattman, 2014, Fiala, 2014 & Hirshleifer et al., 2014). It also includes income at household level. |
| **Family health and education:** This includes reproductive health, medical expenses, household nutrition, hygiene, education expenditure etc. (Blattman at al., 2014). |
| **Inclusion and empowerment (social network):** Personal empowerment is about taking control of your own life, and making positive decisions based on what you want (Mind Tools, 2019). Outcomes include but not limited to quality of family relationships, partner relations and autonomy e.g.,' to spend earnings buy without permission from partner (Blattman at al. ,2014). Engagement in community activities should be coded here. |

Annex 3: Reference List for definitions of Interventions and Outcomes

1. Abebe, G. T., Caria, S., Fafchamps, M., Falco, P., Franklin, S., Quinn, S., & Shilpi, F. (2017). Job fairs: Matching firms and workers in a field experiment in Ethiopia. *World Bank Policy Research Working Paper*, (8092). Report: http://www.simonrquinn.com/JobFairsExperiment.pdf. Accessed 9 December 2019
2. Acevedo, P., Cruces, G., Gertler, P., & Martinez, S. (2017). *Living up to expectations: How job training made women better off and men worse off* (No. w23264). National Bureau of Economic Research. Report: http://www.nber.org/papers/w23264. Accessed 3 March 2020.
3. Ahmed, A., Chakravarty, S., Lundberg, M., & Nikolov, P. (2014). *The Role of Training Programs for Youth Employment in Nepal: Impact Evaluation Report on the Employment Fund.* World Bank. Report: http://users.nber.org/~nikolovp/pubs/Neap_paper.pdf. Accessed 28 February 2020.
4. Azevedo, T. A. D., Davis, J., & Charles, M. (2013). Testing what works in youth employment: Evaluating Kenya's Ninaweza program. *Baltimore, Maryland: International Youth Foundation*.
5. Beam, E. A. (2016). Do job fairs matter? Experimental evidence on the impact of job-fair attendance. *Journal of Development Economics*, *120*, 32-40.
6. Betcherman, G., Daysal, N. M., & Pagés, C. (2010). Do employment subsidies work? Evidence from regionally targeted subsidies in Turkey. *Labour Economics*, *17*(4), 710-722. https://www.sciencedirect.com/science/article/abs/pii/S092753710900150X
7. Blattman, C., Fiala, N., & Martinez, S. (2014). Generating skilled self-employment in developing countries: Experimental evidence from Uganda. *The Quarterly Journal of Economics*, *129*(2), 697-752.
8. Bordos, K., Csillag, M., & Scharl, A. (2015). *What works in wage subsidies for young people: A review of issues, theory, policies and evidence. ILO Working Papers, (994898973402676). https://www.ilo.org/wcmsp5/groups/public/ed_emp/documents/publication/wcms_466538.pdf. Report: Accessed 19 December 2019.*
9. Business Dictionary. (2020). *Cost.* http://www.businessdictionary.com/definition/cost.html. Accessed 3 March 2020.
10. Business Directory. (2019). *Length of Service.* http://www.businessdictionary.com/definition/length-of-service.html. Accessed 20 December 2019.
11. CAPLA. (2019). *What is Prior Learning Assessment & Recognition (PLAR)/ Recognition of Prior Learning (RPL)?* http://capla.ca/what-is-rpl/. Accessed 19 December 2019.
12. Cellini, S. R., & Kee, J. E. (2010). Cost-effectiveness and cost-benefit analysis. *Handbook of practical program evaluation*, *3*. https://onlinelibrary.wiley.com/doi/10.1002/9781119171386.ch24. Accessed 10 December 2019.
13. Centre for Financial Inclusion. (2019). *Financial Inclusion Glossary*. https://www.centerforfinancialinclusion.org/financial-inclusion-glossary. Accessed 20 December 2019.
14. Chen, A. 2018, Grant, Investopedia. https://www.investopedia.com/terms/g/grant.asp, Accessed 28 February 2020.
15. Clarke, M., & Chalmers, I. (1998). Discussion sections in reports of controlled trials published in general medical journals: islands in search of continents?. *Jama*, *280*(3), 280-282.
16. EU. (2017). *EU Delegation to the Republic of Serbia: Public Works- a solution to unemployment.* https://www.searchnewworld.com/search/search2.html?partid=rolbng&p=public+works+and+unemployment&subid=004. Accessed 3 March 2020.
17. Eurofund. (2019). *Job Quality*. https://www.eurofound.europa.eu/topic/job-quality. Accessed 2o December 2019.
18. Farlex Financial Dictionary. (2012). *Job Creation*. https://financial-dictionary.thefreedictionary.com/Job+Creation. Accessed 20 December 2019.
19. Fiala, N. (2013). Stimulating microenterprise growth: Results from a loans, grants and training experiment in Uganda. *Grants and Training Experiment in Uganda (December 4, 2013)*.
20. Finch, C. (2018) *Difference Between Internship & Apprenticeship.* https://work.chron.com/difference-between-internship-apprenticeship-29606.html. Accessed 20 December 2019.
21. Flynn, R. (2013). *A Case Study of Rural Finance Self-Help Groups in Uganda and Their Impact on Poverty Alleviation and Development. Report: https://digitalcollections.sit.edu/isp_collection/1688. Accessed 20 December 2019.*
22. Forbes. (2019). *Small Business Ownership: Start Out Small With A Microfranchise*, https://www.forbes.com/sites/allbusiness/2013/11/27/small-business-ownership-start-out-small-with-a-microfranchise/#65e6553e5a3c . Accessed 20 December 2019.
23. Government of Netherlands. (2019). *Forms of crime*. https://www.government.nl/topics/crime-and-crime-prevention/forms-of-crime . Accessed 20 December 2019.
24. Granicus. (2019). *What Is Digital Government Service?* https://granicus.com/dictionary/digital-government-services/. Accessed 20 December 2019.
25. Heavey, D. (2017). *Examples of Successful Media Campaigns*. https://thrivehive.com/examples-social-media-campaigns/. Accessed 28 February 2020.
26. Hirshleifer, S., McKenzie, D., Almeida, R., & Ridao‐Cano, C. (2016). The impact of vocational training for the unemployed: experimental evidence from Turkey. *The Economic Journal*, *126*(597), 2115-2146.
27. HR. (2019). *The Differences between Reskilling and Upskilling. Blog*. https://www.hrinasia.com/general/the-differences-between-reskilling-and-upskilling/. Accessed 20 December 2019.
28. Ibarraran, P., Ripani, L., Taboada, B., Villa, J. M., & Garcia, B. (2014). Life skills, employability and training for disadvantaged youth: Evidence from a randomized evaluation design. *IZA Journal of Labor & Development*, *3*(1), 1-24.
29. International Labour Organization. (2010). *A skilled workforce for strong, sustainable and balanced growth: a G20 training strategy. Report:* https://www.ilo.org/skills/pubs/WCMS_151966/lang--en/index.htm. Accessed 20 December 2019.
30. International Labour Organization. (2017). *World social protection report 2017–19: Universal social protection to achieve the Sustainable Development Goals. Report: https://www.ilo.org/global/publications/books/WCMS_604882/lang--en/index.htm. Accessed 28 February 2020.*
31. International Labour Organization. (2019). *Conventions and Recommendations*. https://www.ilo.org/global/standards/introduction-to-international-labour-standards/conventions-and-recommendations/lang--en/index.htm. Accesed19 December 2019.
32. International Labour Organization. (2020). *Social Protection.* https://www.ilo.org/global/topics/social-security/lang--en/index.htm. Accessed 28 February 2010.
33. International Labour Organization. (n.d.). Employment Research Brief: Employment Policy Implementation Mechanisms across countries. https://www.ilo.org/wcmsp5/groups/public/---ed_emp/documents/publication/wcms_613372.pdf. Accessed 3 March 2020.
34. Innovaccess - Intellectual Property Portal. (2019). *Business creation.* http://www.innovaccess.eu/business-creation. Accessed 20 December 2019.
35. International Labour Office. Skills and Employability Department (EMP/SKILLS). (2012). *Upgrading informal apprenticeship: a resource guide for Africa*. International Labour Office, Geneva, Switzerland. Report: https://www.ilo.org/skills/pubs/WCMS_171393/lang--en/index.htm. Accessed 19 December 2019.
36. Investopedia. (2019a). *Crowdfunding*. https://www.investopedia.com/terms/c/crowdfunding.asp. Accessed 20 December 2019.
37. Investopedia. (2019b). *Microfinance*. https://www.investopedia.com/terms/m/microfinance.asp. Accessed 20 December 2019.
38. Invetopedia 2019, Social Impact Bond (SIB). https://www.investopedia.com/terms/s/social-impact-bond.asp. Accessed 20 December 2019.
39. Kagan, J.(2019). *Guaranteed Loan – Definition, Inestopedia.* https://www.investopedia.com/terms/g/guaranteed-loan.asp. Accessed 28 February 2020.
40. Kluve, J., Puerto, S., Stoeterau, J., Weidenkaff, F., Witte, M., Robalino, D., ... & Rother, F. (2014). Protocol: Interventions to improve labour market outcomes of youth: A systematic review of active labour market programmes. *Campbell Systematic Reviews*, *10*(1), 1-109. Protocol: https://onlinelibrary.wiley.com/doi/full/10.1002/CL2.12320. Accessed 10 December 2019.
41. Lewin, K. M. Educational access, equity, and development: planning to make rights realities. https://unesdoc.unesco.org/ark:/48223/pf0000235003. Accessed 10 December 2019.
42. Lippman, L. H., Ryberg, R., Carney, R., & Moore, K. A. (2015). Workforce Connections: Key “soft skills” that foster youth workforce success: toward a consensus across fields. *Washington, DC: Child Trends*. https://www.childtrends.org/wp-content/uploads/2015/06/2015-24WFCSoftSkills1.pdf. Accessed 10 February 2020.
43. Longman. (2019). *Longman Business Dictionary*. https://www.ldoceonline.com/dictionary/job-placement. Accessed 20 December 2019.
44. McGuire, G. (2002). *Trade in services: market access opportunities and the benefits of liberalization for developing economies* (Vol. 19). Univ of California Press.
45. Mind Tools. (2019). *What Is Personal Empowerment?* https://www.mindtools.com/pages/article/personal-empowerment.htm. Accessed 20 December 2019.
46. Nutz, N., & Sievers, M. (2015). *A rough guide to value chain development: How to create employment and improve working conditions in targeted sectors*. ILO. *Report: https://www.ilo.org/wcmsp5/groups/public/---ed_emp/---emp_ent/---ifp_seed/documents/publication/wcms_366005.pdf. Accessed 20 December 2019.*
47. Organization for Economic Co-operation and Development (OECD). (2012). *Transferable Skills Training for Researchers: Supporting Career Development and Research*. OECD Publishing. https://read.oecd-ilibrary.org/science-and-technology/transferable-skills-training-for-researchers_9789264179721-en#page4. Accessed 20 December 2019.
48. Ponizovskiy, V., Grigoryan, L., Kühnen, U., & Boehnke, K. (2019). Social construction of the value–behavior relation. *Frontiers in psychology*, *10*, 934. https://doi.org/10.3389/fpsyg.2019.00934. Accessed 20 December 2019.
49. Program Success Center for Sexual & Reproductive Health. (2019). *Training of Trainers (TOT).* https://www.etr.org/ebi/training-ta/types-of-services/training-of-trainers/. Accessed 19 December 2019.
50. Scism, T. E. (1974). Employee Mobility in the Federal Service: A Description of Some Recent Data. *Public Administration Review*, *34*(3), 247-254
51. Small Business Development Corporation. (2019). *Essential business skills*. https://www.smallbusiness.wa.gov.au/business-advice/starting-your-business/business-skills. Accessed 19 December 2019.
52. UNESCO. (2012). *International Standard Classification of Education ISCED 2011*. Montreal. https://www.google.com/url?sa=t&rct=j&q=&esrc=s&source=web&cd=4&cad=rja&uact=8&ved=2ahUKEwj176K-h8LmAhVuD2MBHf8mAcIQFjADegQIARAC&url=http%3A%2F%2Fuis.unesco.org%2Fsites%2Fdefault%2Ffiles%2Fdocuments%2Finternational-standard-classification-of-education-isced-2011-en.pdf&usg=AOvVaw2BYYJ4Vx3zdUbGuqIGbNCD. Accessed 19 December 2019.
53. UNESCO. (2019*). Glossary****:*** *Non-formal education*. http://uis.unesco.org/en/glossary-term/non-formal-education. Accessed 19 December 2019.
54. UNESCO. (2019a). *TVETipedia Glossary.* https://unevoc.unesco.org/go.php?q=TVETipedia+Glossary+A-Z&term=Technical+and+vocational+education+and+training. Acccessed 19 December 2019.
55. UNESCO. (2019b). *TVETipedia Glossary, On-the-job training (OJT)*. https://unevoc.unesco.org/go.php?q=TVETipedia+Glossary+A-Z&filt=&id=346. Accessed 19 December 2019.
56. UNESCO. (2019c). *Glossary: Completion rate.* http://uis.unesco.org/en/glossary-term/completion-rate. Accessed 20 December 2019.
57. UNESCO. (2019d). *TVETipedia Glossary.* https://unevoc.unesco.org/go.php?q=TVETipedia+Glossary+A-Z&term=Technical+and+vocational+education+and+training. Accessed 20 December 2019.
58. UNESCO. (2019e). *Digital skills critical for jobs and social inclusion.* https://en.unesco.org/news/digital-skills-critical-jobs-and-social-inclusion. Accessed 20 December 2019.
59. USA Legal. (2019). *Employment Status Law and Legal Definition*. https://definitions.uslegal.com/e/employment-status/. Accessed 20 December 2019.
60. USA. Department of Education. (2019*). Career Guidance and Counseling Programs.* https://www2.ed.gov/about/offices/list/ovae/pi/cte/cgcp.html. Accessed 20 December 2019.
61. USA. State of California Department of Industrial Relations. (2020). *Public Works*. https://www.dir.ca.gov/Public-Works/PublicWorks.html. Accessed 28 February 2020.
62. Vlăsceanu, L., Grünberg, L., & Pârlea, D. (2004). *Quality assurance and accreditation: A glossary of basic terms and definitions* (p. 25). Bucharest: Unesco-Cepes. https://www.aracis.ro/wp-content/uploads/2019/08/Glossary_07_05_2007.pd
63. Vvob. (2019). *Our vision on quality education*. https://www.vvob.org/en/education/our-vision-on-quality-education. Accessed 20 December 2019.
64. World Bank. (2020). *Small and Medium Enterprises (SMEs) Finance.* https://www.worldbank.org/en/topic/smefinance. Accessed 28 February 2020.

Annex 4: Search Strategy for the Youth Employment Evidence and Gap Map

1. **ERIC DATABASE:** https://eric.ed.gov/

**With filters: Publication date: Jan 2000-Dec2019; Boolean/phrase/English**

**(Selected Boolean phrase)**

| **Results** | **Type** |
| --- | --- |
| S1 | Train* or retrain* or re-train* or retool* or re-tool* or skill* or educat* or formal or on-the-job or vocational or TVET or OTJ or apprenticeship* or mentor* or internship or upskilling or career or financ* or microfinance or guarantees or policies or policy or micro-franchising or “international labour standard*” or “international labor standard*” or employ*” or ALMPs or “active labour market program*” or “active labor market program*” or “value chain “ or entrepreneur* or cost-effectiveness or "cost per job" or “social protection” or “social security” or “accountability systems” or scholarship* or earn* |
| S2 | Youth* or Teen* or “young people” or “young adult*” or “young person*” or adolescen* or “early adult*” or 'young women' or 'young men' or “aged from 15” or 'over 15 years' or 'under 35 years' |
| 3 | 1 AND 2 |
| 4 | eval* or assess* or analy* or estimate or effect or intervention* or measure* |
| 5 | “random* controlled trial” or “controlled clinical trial” or RCT or “control* trial” or “random allocat*” or “difference in difference*” or “difference-in-difference*” or “double difference*” or “regression discontinuity” or “instrumental variable*” or “propensity score” or quasi-experiment* or “quasi experiment*” or QED or QES or matching or “IV estimation” or “instrumental variable” |
| 6 | “systematic review*” or meta-analy* or “meta analy*” |
| 7 | 4 OR 5 OR 6 |
| 8 | 3 AND 7 |
| 9 | Limiters - Date Published: 20000101-20191231 |
| 10 | Limiters – English language |

1. **JSTOR:** https://www.jstor.org/

| **Results** | **Type** |
| --- | --- |
| 1 | ((ti:(train* OR educat* OR mentor* OR job* OR skill* OR lab*r market OR employ* OR job OR empower*) AND ti:(youth* or teen* or "young people" or adolesc*)) AND ti:(eval* or assess* or analys* or "systematic review*" or RCT)) AND la:(eng OR en) |
| 2 | Limiters – Date published: 20000101-20191231 |

1. **3ie- Impact evaluations:** https://www.3ieimpact.org/sitewide-search?search_api_fulltext=&sort_by=search_api_relevance

| **Results** | **Type** |
| --- | --- |
| 1 | (youth OR young people) AND (educat* OR train* OR technical or vocational) AND (employment OR “labor market” OR “labour market”) |

1. **3ie Database of Systematic Reviews:** http://www.3ieimpact.org/evidence/systematic-reviews/

| **Results** | **Type** |
| --- | --- |
| 1 | (youth OR "young people") AND (educat* OR train* OR technical OR vocational) AND (employ* OR "labor market" OR “labour market ) |

1. **3ie Registry for International Development Impact Evaluations - (RIDIE):** http://ridie.3ieimpact.org/

| **Results** | **Type** |
| --- | --- |
| 1 | (youth or "Young people") + (educat* or train* or "technical and vocational education" or entrepreneur*) + (employ* OR "labor market" OR “labour market”) |

1. **USAID - Development Experience Clearinghouse:** https://dec.usaid.gov/

| **Results** | **Type** |
| --- | --- |
| 1 | Youth employment |

1. **Google Scholar:** https://scholar.google.com/

| **Results** | **Type** |
| --- | --- |
|  | (train* OR educat* OR skill* OR re-tool* OR technical OR vocational OR TVET OR scholarship* OR apprentice*) AND youth* AND (employ* OR empower* OR ALMP OR AND (labour OR labor) OR entrepreneur*) AND (eval* OR “systematic review") |

1. SSRN (Social Science Research Network): http://www.ssrn.com/

| **Results** | **Type** |
| --- | --- |
| 1 | youth employment AND training AND evaluation |

1. Wiley Online: https://onlinelibrary.wiley.com/

| **Results** | **Type** |
| --- | --- |
| 1 | (train* OR skill* OR educat* OR apprenticeship*) in Title AND (youth* OR "young adult*" OR teen* OR "young people") in Title AND (employment OR "labour market" OR “labor market” OR enterpreneur*) anywhere AND (eval* OR “systematic review” OR RCT) anywhere |
|  | Limiters: 2000-2019 |

1. **University of Chicago Journals:** https://www.journals.uchicago.edu/

| **Results** | **Type** |
| --- | --- |
| 1 | (youth* OR "young adult*" OR "young people") AND (employment OR "labour market" OR "labor market") AND (educat* OR train*) in abstract |

1. **World Bank Labor Markets**: http://www.worldbank.org/labormarkets

| **Results** | **Type** |
| --- | --- |
| 1 | In keywords: (youth* OR "young adult*" OR "young people" OR teen*) AND In keyword: (employment OR "labour market" OR "labor market") AND In Keyword: (educat* OR train* OR skill*) AND In keyword: (evaluation OR “systematic review”) |

1. IBSS (International Bibliography of the Social Sciences): https://about.proquest.com/en/products-services/ibss-set-c/
2. **REPEC & World Bank e-library (through EBSCO Discovery):** https://econpapers.repec.org/

| **Results** | **Type** |
| --- | --- |
| S1 | Train* or retrain* or re-train* or retool* or re-tool* or skill* or educat* or formal or on-the-job or vocational or TVET or OTJ or apprenticeship* or mentor* or internship or upskilling or career or financ* or microfinance or guarantees or policies or policy or micro-franchising or “international labour standard*” or “international labor standard*” or employ*” or ALMPs or “active labour market program*” or “active labor market program*” or “value chain “ or entrepreneur* or cost-effectiveness or "cost per job" or “social protection” or “social security” or “accountability systems” or scholarship* or earn* |
| S2 | TI ( (Youth* or Teen* or "young people" or "young adult*" or "young person*" or adolescen* or "early adult*" or "aged from 15" or "aged under 35") ) OR SU ( (Youth* or Teen* or "young people" or "young adult*" or "young person*" or adolescen* or "early adult*" or “young women” or “young men” or "aged from 15" or “over 15 years” or "aged under 35" or ) ) Limiters - Date of Publication: 20000101-20191231  Database - Discovery Service for 3ie, Inc. |
| S3 | TI ( (eval* or assess* or analy* or estimat* or effect or intervention* or measure*) ) OR AB ( (eval* or assess* or analy* or estimat* or effect or intervention* or measure*) ) OR SU ( (eval* or assess* or analy* or estimat* or effect or intervention* or measure*) ) Limiters - Date of Publication: 20000101-20191231  Database - Discovery Service for 3ie, Inc. |
| S4 | TI ( ("random* controlled trial" or "controlled clinical trial" or RCT or "control* trial" or "random* allocat*" or "difference in difference*" or difference-in-difference* or "double difference*" or "regression discontinuity" or "instrumental variable*" or "propensity score" or quasi-experiment* or "quasi experiment*" or QED or QES or matching or "IV estimation") ) OR AB ( ("random* controlled trial" or "controlled clinical trial" or RCT or "control* trial" or "random* allocat*" or "difference in difference*" or difference-in-difference* or "double difference*" or "regression discontinuity" or "instrumental variable*" or "propensity score" or quasi-experiment* or "quasi experiment*" or QED or QES or matching or "IV estimation") ) OR SU ( ("random* controlled trial" or "controlled clinical trial" or RCT or "control* trial" or "random* allocat*" or "difference in difference*" or difference-in-difference* or "double difference*" or "regression discontinuity" or "instrumental variable*" or "propensity score" or quasi-experiment* or "quasi experiment*" or QED or QES or matching or "IV estimation") ) Limiters - Date of Publication: 20000101-20191231  Database - Discovery Service for 3ie, Inc. |
| S5 | TI ( ("systematic review*" or (systematic* N2 review*) or meta-analy* or "meta analy*") ) OR AB ( ("systematic review*" or (systematic* N2 review*) or meta-analy* or "meta analy*") ) OR SU ( ("systematic review*" or (systematic* N2 review*) or meta-analy* or "meta analy*") ) Limiters - Date of Publication: 20000101-20191231  Database - Discovery Service for 3ie, Inc. |
| S6 | S3 OR S4 OR S5  Database - Discovery Service for 3ie, Inc. |
| S7 | S1 AND S2 AND S6  Database - Discovery Service for 3ie, Inc. |
|  | Final Result limited to Repec |
|  | Final Result limited to World Bank e-library |

1. **Institute for the Study of Labour (IZA):** http://www.iza.org

| **Results** | **Type** |
| --- | --- |
| 1 | youth employment AND (evaluation OR systematic review or meta analysis) |

1. **Campbell Collaboration:** https://www.campbellcollaboration.org/

| **Results** | **Type** |
| --- | --- |
| 1 | youth AND employment |

1. **EPPI CENTRE:** https://eppi.ioe.ac.uk/cms/Default.aspx?tabid=185

| **Results** | **Type** |
| --- | --- |
| 1 | Youth |
| 2 | Teenagers |
| 3 | Young adults |
| 4 | Young people |
| 5 | Young women |
| 6 | Young men |
| 7 | Aged from 15 |
| 8 | Over 15 years |
| 9 | Under 35 years |
| 10 | 1-9/or |
| 11 | Education |
| 12 | skill* |
| 13 | Internship |
| 14 | Upskilling |
| 15 | Career |
| 16 | empower* |
| 17 | Micro-franchising |
| 18 | Cost-effectiveness |
| 19 | Cost per job |
| 20 | train* |
| 21 | 11-20/or |
| 22 | 10 AND 21 |
| 23 | Evaluation |
| 24 | “Systematic review” |
| 25 | 12 OR 13 |
| 20 | 11 AND 14 |

1. **ELDIS:** https://www.eldis.org/

| **Results** | **Type** |
| --- | --- |
|  | (Training OR skill* OR educat*) AND Youth AND (employ* OR income) AND evaluation |

19 **Research for Development (DfID’s outputs d/base for funded projects)**: https://www.gov.uk/dfid-research-outputs

| **Results** | **Type** |
| --- | --- |
|  | (educat* OR train* OR skill*) AND youth* AND (employment OR "labour market" OR "labor market") AND ("impact evaluation” OR “systematic review”) |

20. UNDP International Policy Centre for Inclusive Growth (IPC-IG): http://www.ipc-undp.org/

| **Results** | **Type** |
| --- | --- |
|  | Youth employment |

21. International Labour Organization: https://www.ilo.org/Search5/search.do

| **Results** | **Type** |
| --- | --- |
|  | ("youth OR "young people") AND employment AND training AND ("labour market" OR "labor market") AND evaluation |

**22. EconLit:** https://www.aeaweb.org/econlit/

| **Results** | **Type** |
| --- | --- |
| 1 | (Train* or retrain* or re-train* or retool* or re-tool* or skill* or educat* or internship or upskilling or career or formal or on-the-job or vocational or TVET or OTJ or apprenticeship* or mentor* or financ* or microfinance or guarantees or policies or policy or "international labour standard*" or "international labor standard*" or employ* or ALMPs or "active labour market*" or "active labor market*" or "value chain" or entrepreneur* or "social protection" or "social security" or (accountab* adj3 system*) or scholarship* or earn*).ti. |
| 2 | I21 or I22 or I26 or J21 or L26 or M53).cc. |
| 3 | or/1-2 |
| 4 | (Youth* or Teen* or "young people" or "young adult*" or "young person*" or adolescen* or "early adult*" or "aged from 15" or "aged under 35").ti,kw. |
| 5 | J13.cc. |
| 6 | or/4-5 |
| 7 | eval* or assess* or analy* or estimat* or effect or intervention* or measure*).ti,ab. |
| 8 | ("random* controlled trial" or "controlled clinical trial" or RCT or "control* trial" or "random* allocat*" or "difference in difference*" or difference-in-difference* or "double difference*" or "regression discontinuity" or "instrumental variable*" or "propensity score" or quasi-experiment* or "quasi experiment*" or QED or QES or matching or "IV estimation").ti,ab,kw. |
| 9 | ("systematic review*" or (systematic* adj2 review*) or meta-analy* or "meta analy*").ti,ab,kw. |
| 10 | or/7-9 |
| 11 | 3 and 6 and 10 |
| 12 | limit 11 to yr="2000 - 2019" |

**23. CAB Global Health:** https://www.cabi.org/publishing-products/global-health/

| **Results** | **Type** |
| --- | --- |
| 1 | or financ* or microfinance or guarantees or policies or policy or "international labour standard*" or "international labor standard*" or employ* or ALMPs or "active labour market*" or "active labor market*" or "value chain" or entrepreneur* or "social protection" or "social security" or (accountab* adj3 system*) or scholarship* or earn*).ti. |
| 2 | employment/ or employment opportunities/ or labour economics/ or labour market/ or inservice training/ or apprenticeship/ or on-the-job training/ or labour market/ or vocational training/ or job skills/ or entrepreneurship/ |
| 3 | or/1-2 |
| 4 | (Youth* or Teen* or "young people" or "young adult*" or "young person*" or adolescen* or "early adult*" or "aged from 15" or "aged under 35").ti. |
| 5 | adolescents/ or youth/ or young workers/ or rural youth/ or young adults/ |
| 6 | or/4-5 |
| 7 | (eval* or assess* or analy* or estimat* or effect or intervention* or measure*).ti,ab. |
| 8 | ("random* controlled trial" or "controlled clinical trial" or RCT or "control* trial" or "random* allocat*" or "difference in difference*" or difference-in-difference* or "double difference*" or "regression discontinuity" or "instrumental variable*" or "propensity score" or quasi-experiment* or "quasi experiment*" or QED or QES or matching or "IV estimation").ti,ab. |
| 9 | ("systematic review*" or (systematic* adj2 review*) or meta-analy* or "meta analy*").ti,ab. |
| 10 | or/7-10 |
| 11 | 3 and 6 and 11 |
| 12 | limit 12 to yr="2000 - 2019" |

Annex 5: Coding sheet used in the youth employment EGM

| **Bibliographic Information** | |  |
| --- | --- | --- |
|  | Title |  |
|  | Author(s) |  |
|  | Month |  |
|  | Year |  |
|  | Publication Type |  |
|  | Abstract |  |
|  | Journal title or Report Series |  |
|  | URL |  |
|  | Volume |  |
|  | Publisher |  |
|  | DOI |  |
|  | Short title [First author (date)] |  |
|  | Edition |  |
|  | ISBN/ISSN |  |
|  | Issue |  |
|  | Institution |  |
| **Filters** |  |  |
| Publication status | Complete |  |
|  | On-going |  |
| Region of the World | Sub-Saharan Africa |  |
|  | Latin America and Caribbean |  |
|  | East Asia and Pacific |  |
|  | South Asia |  |
|  | Europe and Central Asia |  |
|  | North America |  |
| World Bank Region | Low income countries |  |
|  | Lower middle income |  |
|  | Upper middle income |  |
|  | High income |  |
| Population | Youth aged 15-19 |  |
|  | Youth aged 20-24 |  |
|  | Youth aged 25-29 |  |
|  | Youth aged 30-35 |  |
|  | Age not reported |  |
|  | Male |  |
|  | Female |  |
|  | Gender not reported |  |
|  | Urban |  |
|  | Rural |  |
|  | Rural/urban not reported |  |
|  | Youth with disabilities |  |
|  | Youth in fragility, conflict and violence (FCV) contexts |  |
|  | Youth from disadvantaged background (low-income families or low education) |  |
|  | Criminal background |  |
|  | Ethnic minority |  |
|  | Humanitarian settings |  |
| Sectors | Agriculture |  |
|  | Services |  |
|  | Industry: Non-manufacturing (construction, mining, quarrying, electricity, gas and water supply) |  |
|  | Industry: Manufacturing |  |
|  | Sector not reported or not–relevant |  |
| Implementer (specify) | NGO, multilateral, government, researcher, private sector (specify in this open field) |  |
| Size of the intervention | Number and units in the programme (firm or individual or school in the programme not necessarily only those sampled for the study) |  |
| Cost | Cost of intervention |  |
| Setting for the intervention | High school |  |
|  | Tertiary education |  |
|  | Training centre |  |
|  | Firm |  |
|  | Other (specify) |  |
| Study design | Experimental designs (i.e. RCTs including cluster randomisation) |  |
|  | Non-experimental matching designs (i.e. RDD, PSM, DID/CBA, ITS) |  |
|  | Regression-based approaches (i.e. IVs; excludes RDD) |  |
| **Interventions** |  |  |
| Training, up-skilling and retraining/ re-skilling | Prior Learning Assessment and Recognition (PLAR) |  |
|  | Education, technical and vocational training (TVET) |  |
|  | Internship and apprenticeship |  |
|  | Training centre accreditation and certification |  |
|  | Training of trainers and teachers |  |
|  | Business skills training |  |
|  | Life skills training |  |
| Support to employment | Employee Mentoring (Work integrated learning; on job training) |  |
|  | Career offices/advisory services/career days |  |
|  | Programme for overseas employment |  |
|  | Public work programs |  |
|  | Support to employee mobility and placements |  |
|  | Wage subsidies |  |
| Decent work policies | Labour standards |  |
|  | Social protection and social security |  |
|  | Accountability systems |  |
| Information | Labour market information |  |
|  | Digital services and SMS coaching |  |
|  | Social media campaigns and awareness campaigns |  |
|  | Value chain development |  |
|  | Access to services and markets (value chains) |  |
| Entrepreneurship promotion and financing | Small and medium sized Enterprise finance (SME) |  |
|  | Microfinance (to individuals) |  |
|  | Social impact bonds |  |
|  | Crowd funding |  |
|  | Loan guarantees |  |
|  | Grants |  |
|  | Self-financing groups |  |
|  | Micro-franchising |  |
| **Outcomes** |  |  |
| Category | Sub-Category |  |
| Economic | Costs |  |
|  | Cost Benefit |  |
|  | Cost effectiveness |  |
|  | Multiplier, displacement and spill over effects (Effects not directly in the programme e.g. youth spending earnings to improve local commerce, job displacement) |  |
| Education and skills | Education completion and qualifications |  |
|  | Access to/in education |  |
|  | Education quality |  |
|  | Technical skills & vocational training |  |
|  | Digital skills |  |
|  | Transferable skills (including life and social skills e.g. networking, negotiation) |  |
| Entrepreneurship | Access to financial services |  |
|  | Business creation |  |
|  | Business performance |  |
|  | Job creation (Jobs for other people e.g. number of employees) |  |
| Employment | Vacancies |  |
|  | Actively seeking employment |  |
|  | Employment expectation |  |
|  | Employment status (including duration) |  |
|  | Employment consistent with education/training |  |
|  | Hours worked |  |
|  | Job quality (include formal vs. informal here) |  |
|  | Earnings and salary |  |
| Welfare | Economic outcomes (except earnings). This also includes income at household level |  |
|  | Criminal and delinquent behaviour (antisocial behaviour) |  |
|  | Citizenship, values and social behaviour [Social behaviour is such things as taking part in community activities (clarifying to distinguish from anti-social behaviour). Social behaviour: alcohol/drugs, hanging out with friends] |  |
|  | Family health & education |  |
|  | Inclusion and empowerment (social network). [Engagement in community activities is here (not social behaviour)] |  |

Annex 6: Results of Systematic Reviews Risk of Bias assessment (report for only AMSTAR 2 critical flaws items)

| **Item** | **Title** | **2*. Did the report of the review contain an explicit statement that the review methods were established prior to the conduct of the review and did the report justify any significant deviations from the protocol?** | **7*. Did the review authors provide a list of excluded studies and justify the exclusions?** | **4*. Did the review authors use a comprehensive literature search strategy?** | **13*. Did the review authors account for RoB in individual studies when interpreting/ discussing the results of the review?** | **15*. If they performed quantitative synthesis did the review authors carry out an adequate investigation of publication bias (small study bias) and discuss its likely impact on the results of the review?** |
| --- | --- | --- | --- | --- | --- | --- |
| Lindsay, (2015) (ID:44031017) | A systematic review of mentorship programs to facilitate transition to post-secondary education and employment for youth and young adults with disabilities | -No | -Yes: if it includes the following | -Yes: Should have all the following | -Yes if |  |
| Arif (2019) (ID:44031030) | Work-focused interventions that promote the labour market transition of young adults with chronic disabling health conditions: a systematic review | -Yes: The authors state that they had a written protocol or guide that included ALL the following | -Yes: if it includes the following | -Yes: Should have all the following | -Yes if |  |
| Cobb (2009) (ID:45913829) | Transition Planning/Coordinating Interventions for Youth with Disabilities: A Systematic Review | -No | -No | -Partial yes: All the following | -No | -No |
| Mawn (2017) (ID:45956688) | Are we failing young people not in employment, education or training (NEETs)? A systematic review and meta-analysis of re-engagement interventions | -Yes: The authors state that they had a written protocol or guide that included ALL the following | -Partial Yes if: | -Yes: Should have all the following | -Yes if | -No |
| Esther (2016) (ID:44473427) | Interventions for promoting reintegration and reducing harmful behaviour and lifestyles in street-connected children and young people: a systematic review | -Yes: The authors state that they had a written protocol or guide that included ALL the following | -Yes: if it includes the following | -Yes: Should have all the following | -Yes if | -No |
| Grimm (2015) (ID:45357776) | Do interventions targeted at micro-entrepreneurs and small and medium-sized firms create jobs? A systematic review of the evidence for low and middle income countries | -No | -Yes: if it includes the following | -Yes: Should have all the following | -No | -No -No meta-analysis conducted |
| Catalano (2019) (ID:45357780) | Positive youth development programs in low-and middle-income countries: a conceptual framework and systematic review of efficacy | -No | -Yes: if it includes the following | -No | -No | -No meta-analysis conducted |
| Hanif, (2017) (ID:50070497) | A systematic review of vocational interventions for youth with physical disabilities. factors in studying employment for persons with disability: how the picture can change. | -Yes: The authors state that they had a written protocol or guide that included ALL the following | -No | -Partial yes: All the following | -No | -No meta-analysis conducted |
| Ke, (2018) (ID:44031025) | Social Skill Interventions for Youth and Adults With Autism Spectrum Disorder: A Systematic Review | -No | -Yes: if it includes the following | -Partial yes: All the following | -No | -No meta-analysis conducted |
| Jennings (2014) (ID:45956199) | Do men need empowering too? A systematic review of entrepreneurial education and microenterprise development on health disparities among inner-city black male youth | -No | -Yes: if it includes the following | -Yes: Should have all the following | -No | -No meta-analysis conducted |
| John (2013) (ID:44473426) | Pre-Graduation Transition Services for Improving Employment Outcomes among Persons with Autism Spectrum Disorders: A Systematic Review | -Yes: The authors state that they had a written protocol or guide that included ALL the following | -Yes: if it includes the following | -Yes: Should have all the following | -No | -No meta-analysis conducted |
| Lindsay (2018) (ID:60940285) | Electronic Mentoring Programs and Interventions for Children and Youth With Disabilities: Systematic Review | -No | -Yes: if it includes the following | -Yes: Should have all the following | -Yes if | -No meta-analysis conducted |
| Janice (2015) (ID:44472911) | Interventions to Improve the Labour Market Situation of Adults with Physical and/or Sensory Disabilities in Lowand Middle-Income Countries: A Systematic Review | -Yes: The authors state that they had a written protocol or guide that included ALL the following | -Yes: if it includes the following | -Yes: Should have all the following | -Yes if | -No meta-analysis conducted |
| Patrick (2013) (ID:44473425) | Mentoring Interventions to Affect Juvenile Delinquency and Associated Problems: A Systematic Review | -Yes: The authors state that they had a written protocol or guide that included ALL the following | -Yes: if it includes the following | -Yes: Should have all the following |  | -Yes if |
| Sandra (2011) (ID:44473430) | Dropout Prevention and Intervention Programs: Effects on School Completion and Dropout among School-aged Children and Youth | -Yes: The authors state that they had a written protocol or guide that included ALL the following | -Yes: if it includes the following | -Yes: Should have all the following | -No | -Yes if |
| Kluve (2017) (ID:44031027) | Interventions to improve the labour market outcomes of youth: a systematic review | -Yes: The authors state that they had a written protocol or guide that included ALL the following | -Yes: if it includes the following |  | -Yes if | -Yes if |
| Marjorie (2017) (ID:44473225) | Vocational and business training to improve women's labour market outcomes in low- and middle-income countries: a systematic review | -Yes: The authors state that they had a written protocol or guide that included ALL the following | -Yes: if it includes the following | -Yes: Should have all the following | -Yes if | -Yes if |
| Matthew (2011) (ID:44473419) | Youth Empowerment Programs for Improving Self-Efficacy and Self-Esteem of Adolescents | -Yes: The authors state that they had a written protocol or guide that included ALL the following | -Yes: if it includes the following | -Yes: Should have all the following | -Yes if | -Yes if |
| Piza (2016) (ID:44473428) | The impact of business support services for small and medium enterprises on firm performance in low- and middle-income countries | -Yes: The authors state that they had a written protocol or guide that included ALL the following | -Yes: if it includes the following | -Yes: Should have all the following | -Yes if | -Yes if |
| Trine (2015) (ID:44473421) | Active Labour Market Programme Participation for Unemployment Insurance Recipients: A Systematic Review | -Yes: The authors state that they had a written protocol or guide that included ALL the following | -Yes: if it includes the following | -Yes: Should have all the following | -Yes if | -Yes if |
| Tripney (2013) (ID:44031812) | Post-basic technical and vocational education and training (TVET) interventions to improve employability and employment of TVET graduates in low-and middle-income countries: A systematic review | -Yes: The authors state that they had a written protocol or guide that included ALL the following | -Yes: if it includes the following | -Yes: Should have all the following | -Yes if | -Yes if |

**Annex 7: The EGM 399 Included studies with quality rating**

| **Study with EPPI Reviewer ID** | **Title** | **Study quality** |
| --- | --- | --- |
| Arif (2019) (ID:44031030) | Work-focused interventions that promote the labour market transition of young adults with chronic disabling health conditions: a systematic review | -Medium and high quality systematic review |
| Esther (2016) (ID:44473427) | Interventions for promoting reintegration and reducing harmful behaviour and lifestyles in street-connected children and young people: a systematic review | -Medium and high quality systematic review |
| Janice (2015) (ID:44472911) | Interventions to Improve the Labour Market Situation of Adults with Physical and/or Sensory Disabilities in Lowand Middle-Income Countries: A Systematic Review | -Medium and high quality systematic review |
| John (2013) (ID:44473426) | Pre-Graduation Transition Services for Improving Employment Outcomes among Persons with Autism Spectrum Disorders: A Systematic Review | -Medium and high quality systematic review |
| Kluve (2017) (ID:44031027) | Interventions to improve the labour market outcomes of youth: a systematic review | -Medium and high quality systematic review |
| Lindsay (2018) (ID:60940285) | Electronic Mentoring Programs and Interventions for Children and Youth With Disabilities: Systematic Review | -Medium and high quality systematic review |
| Lindsay, (2015) (ID:44031017) | A systematic review of mentorship programs to facilitate transition to post-secondary education and employment for youth and young adults with disabilities | -Medium and high quality systematic review |
| Marjorie (2017) (ID:44473225) | Vocational and business training to improve women's labour market outcomes in low- and middle-income countries: a systematic review | -Medium and high quality systematic review |
| Matthew (2011) (ID:44473419) | Youth Empowerment Programs for Improving Self-Efficacy and Self-Esteem of Adolescents | -Medium and high quality systematic review |
| Mawn (2017) (ID:45956688) | Are we failing young people not in employment, education or training (NEETs)? A systematic review and meta-analysis of re-engagement interventions | -Medium and high quality systematic review |
| Patrick (2013) (ID:44473425) | Mentoring Interventions to Affect Juvenile Delinquency and Associated Problems: A Systematic Review | -Medium and high quality systematic review |
| Piza (2016) (ID:44473428) | The impact of business support services for small and medium enterprises on firm performance in low- and middle-income countries | -Medium and high quality systematic review |
| Sandra (2011) (ID:44473430) | Dropout Prevention and Intervention Programs: Effects on School Completion and Dropout among School-aged Children and Youth | -Medium and high quality systematic review |
| Trine (2015) (ID:44473421) | Active Labour Market Programme Participation for Unemployment Insurance Recipients: A Systematic Review | -Medium and high quality systematic review |
| Tripney (2013) (ID:44031812) | Post-basic technical and vocational education and training (TVET) interventions to improve employability and employment of TVET graduates in low-and middle-income countries: A systematic review | -Medium and high quality systematic review |
| Medina, C (2013) (ID:45357846) | The Unemployment Subsidy Program in Colombia: An assessment. | -Medium and high quality primary study |
| Abebe (2018) (ID:50331736) | Job Search and Labour Market Exclusion in a Growing African City | -Medium and high quality primary study |
| Abebe (2019) (ID:50331737) | Job fairs: Matching firms and workers in a field experiment in Ethiopia | -Medium and high quality primary study |
| Acevedo, (2017) (ID:45402347) | Living Up to Expectations: How Job Training Made Women Better Off and Men Worse Off | -Medium and high quality primary study |
| Ahmad, (2009) (ID:45458519) | The effect of sanctions and active labour market programs on the exit rate from unemployment | -Medium and high quality primary study |
| Ahmed (2014) (ID:45357754) | The role of training programs for youth employment in Nepal: Interim impact evaluation report on the Employment Fund | -Medium and high quality primary study |
| Ahmed(2014) (ID:45077288) | The role of skills training for youth employment in Nepal: An impact evaluation of the employment fund. Adolescent Girls Initiative (AGI) | -Medium and high quality primary study |
| Albanese (2017) (ID:45931863) | The Effects of Youth Labor Market Reforms: Evidence from Italian Apprenticeships | -Medium and high quality primary study |
| Alexander (2012) (ID:47955157) | Marketable job skills for high school students: what we learned from an evaluation of After School Matters. | -Medium and high quality primary study |
| Alfonsi (2020) (ID:45357717) | Tackling Youth Unemployment: Evidence from a Labor Market Experiment in Uganda | -Medium and high quality primary study |
| Almeida (2010) (ID:43990644) | Jump-starting self-employment? Evidence for welfare participants in Argentina | -Medium and high quality primary study |
| Alzúa (2014) (ID:44048947) | Youth training programs beyond employment. Experimental evidence from Argentina | -Medium and high quality primary study |
| Alzúa (2019) (ID:47257111) | Demand-Driven Youth Training Programs: Experimental Evidence from Mongolia | -Medium and high quality primary study |
| Arandarenko (2014) (ID:45357755) | Impact evaluation of employment programmes targeting disadvantaged youth in Serbia | -Medium and high quality primary study |
| Araya (2019) (ID:50331775) | The Effects of Working while in School: Evidence from Uruguayan Lotteries | -Medium and high quality primary study |
| Attanasio (2011) (ID:44048933) | Subsidizing vocational training for disadvantaged youth in Colombia: evidence from a randomized trial | -Medium and high quality primary study |
| Attanasio, (2008) (ID:44463946) | Training disadvantaged youth in Latin America: evidence from a randomized trial | -Medium and high quality primary study |
| Attanasio, (2009) (ID:44463947) | Subsidizing vocational training for disadvantaged youth in developing countries: evidence from a randomized trial | -Medium and high quality primary study |
| Balcazar (2012) (ID:47942306) | Improving the Transition Outcomes of Low-Income Minority Youth with Disabilities | -Medium and high quality primary study |
| Bandiera (2015) (ID:50331793) | Women's economic empowerment in action: Evidence from a randomised control trial in Africa | -Medium and high quality primary study |
| Batchuluun, (2017) (ID:47256200) | Impact of short term vocational training on youth unemployment: Evidence from Mongolia | -Medium and high quality primary study |
| Beam, (2016) (ID:45402374) | Do job fairs matter? Experimental evidence on the impact of job fair attendance | -Medium and high quality primary study |
| Behaghel, (2012) (ID:45458521) | Private and public provision of counseling to job seekers: Evidence from a large controlled experiment | -Medium and high quality primary study |
| Bell (2012) (ID:47942361) | Mentoring Transition-Age Youth With Blindness | -Medium and high quality primary study |
| Berg (2009) (ID:50158884) | Youth Action Research for Prevention: A Multi-level Intervention Designed to Increase Efficacy and Empowerment Among Urban Youth | -Medium and high quality primary study |
| Bergin (2007) (ID:47942378) | Effects of a college access program for youth underrepresented in higher education: A randomized experiment | -Medium and high quality primary study |
| Betancourt (2014) (ID:44039985) | A behavioral intervention for war-affected youth in Sierra Leone: a randomized controlled trial | -Medium and high quality primary study |
| Blanco (2013) (ID:45357760) | Bounds on average and quantile treatment effects of Job Corps training on wages | -Medium and high quality primary study |
| Blattman (2011) (ID:45879036) | Employment generation in rural Africa: mid-term results from an experimental evaluation of the Youth Opportunities Program in Northern Uganda | -Medium and high quality primary study |
| Blattman (2014) (ID:44048937) | Employing and Empowering Marginalized Women: A Randomized Trial of Microenterprise Assistance | -Medium and high quality primary study |
| Blattman (2019) (ID:45932368) | Impacts of Industrial and Entrepreneurial Jobs on Youth: 5-year Experimental Evidence on Factory Job Offers and Cash Grants in Ethiopia | -Medium and high quality primary study |
| Blattman, (2014) (ID:44474407) | Generating skilled self-employment in developing countries: Experimental evidence from Uganda. | -Medium and high quality primary study |
| Blattman, (2016) (ID:44480066) | The returns to microenterprise support among the ultrapoor: A field experiment in postwar Uganda. | -Medium and high quality primary study |
| Bockerman (2009) (ID:45932387) | Minimum Wages and Youth Employment: Evidence from the Finnish Retail Trade Sector | -Medium and high quality primary study |
| Botha, (2013) (ID:45357853) | Measuring the effectiveness of the Women Entrepreneurship Programme on potential, start-up and established women entrepreneurs in South Africa. | -Medium and high quality primary study |
| Broecke (2013) (ID:45357764) | Tackling graduate unemployment in North Africa through employment subsidies: A look at the SIVP programme in Tunisia | -Medium and high quality primary study |
| Brudevold-Newman (2017) (ID:47985926) | A firm of one's own: experimental evidence on credit constraints and occupational choice | -Medium and high quality primary study |
| Bruhn (2013) (ID:44048940) | Stimulating managerial capital in emerging markets: the impact of business training for young entrepreneurs | -Medium and high quality primary study |
| Calderone (2017) (ID:50331824) | Are there different spillover effects from cash transfers to men and women? Impacts on investments in education in post-war Uganda | -Medium and high quality primary study |
| Card (2011) (ID:44048990) | The labor market impacts of youth training in the Dominican Republic | -Medium and high quality primary study |
| Chakravorty (2017) (ID:47256391) | Skills Training and Employment Outcomes in Rural Bihar | -Medium and high quality primary study |
| Chong (2006) (ID:44048996) | Training quality and earnings: the effects of competition on the provision of public-sponsored training programs | -Medium and high quality primary study |
| Corseuil (2019) (ID:47256456) | Apprenticeship as a stepping stone to better jobs: Evidence from Brazilian matched employer-employee data | -Medium and high quality primary study |
| Crépon (2018) (ID:50581295) | Creating new positions? direct and indirect effects of a subsidized apprenticeship program | -Medium and high quality primary study |
| Cummings (2018) (ID:45861801) | Forging a Path: Final Impacts and Costs of New York City’s Young Adult Internship Program | -Medium and high quality primary study |
| de (2012) (ID:45402405) | One-Time Transfers of Cash or Capital Have Long-Lasting Effects on Microenterprises in Sri Lanka | -Medium and high quality primary study |
| De (2012) (ID:44480070) | Business training and female enterprise start-up, growth, and dynamics: Experimental evidence from Sri Lanka. | -Medium and high quality primary study |
| De (2013) (ID:44480069) | Testing what works in youth employment: Evaluating Kenya's Ninaweza program. | -Medium and high quality primary study |
| Dhaval (2008) (ID:47256528) | Effects of Welfare Reform on Educational Acquisition of Young Adult Women | -Medium and high quality primary study |
| Dixon (2007) (ID:44048961) | Youth Opportunity Grant Initiative: Impact and Synthesis Report | -Medium and high quality primary study |
| Dorsett (2006) (ID:45357771) | The new deal for young people: effect on the labour market status of young men | -Medium and high quality primary study |
| Ehlert (2012) (ID:45934058) | Temporary Work as an Active Labor Market Policy: Evaluating an Innovative Program for Disadvantaged Youths | -Medium and high quality primary study |
| Espinoza, (2011) (ID:44461274) | Impact evaluation of a job-training programme for disadvantaged youths: the case of Projoven. | -Medium and high quality primary study |
| Fairlie, (2015) (ID:45458492) | Behind the GATE Experiment: Evidence on effects of and rationales for subsidized entrepreneurship training | -Medium and high quality primary study |
| Fiala, (2014) (ID:44480072) | Stimulating microenterprise growth: Results from a loans, grants and training experiment in Uganda. | -Medium and high quality primary study |
| Field, (2010) (ID:44480161) | Do traditional institutions constrain female entrepreneurship? A field experiment on business training in India. American Economic Review, 100(2), 125-129. | -Medium and high quality primary study |
| Fraker, (2018) (ID:50070500) | The youth transition demonstration project in Miami, Florida: Design, implementation, and three-year impacts. | -Medium and high quality primary study |
| Franklin (2016) (ID:45357772) | Location, Search Costs, and Youth Unemployment: Experimental Evidence from Transport Subsidies in Addis Ababa | -Medium and high quality primary study |
| Galdo (2012) (ID:45357767) | Does the quality of public-sponsored training programs matter? Evidence from bidding processes data | -Medium and high quality primary study |
| Godlonton, (2016) (ID:45402474) | Employment Exposure: Employment and Wage Effects in Urban Malawi (Working Paper). | -Medium and high quality primary study |
| Groh (2012) (ID:44048970) | Soft skills or hard cash? The impact of training and wage subsidy programs on female youth employment in Jordan | -Medium and high quality primary study |
| Groh (2014) (ID:44048971) | Testing the importance of search frictions, matching, and reservation prestige through randomized experiments in Jordan | -Medium and high quality primary study |
| Groh (2016) (ID:47984597) | The Impact of Soft Skills Training on Female Youth Employment | -Medium and high quality primary study |
| Groh, (2016) (ID:45404432) | Do wage subsidies provide a stepping-stone to employment for recent college graduates? Evidence from a randomized experiment in Jordan | -Medium and high quality primary study |
| Hicks (2013) (ID:44048977) | Vocational education in Kenya: Evidence from a randomized evaluation among youth | -Medium and high quality primary study |
| Hirshleifer, (2014) (ID:44480453) | The impact of vocational training for the unemployed: Experimental evidence from Turkey. | -Medium and high quality primary study |
| Ibarrarán (2019) (ID:50581290) | Experimental Evidence on the Long-Term Effects of a Youth Training Program | -Medium and high quality primary study |
| Ibarrarán, (2015) (ID:45357844) | Experimental evidence on the long-term impacts of a youth training program. | -Medium and high quality primary study |
| Izzo (2000) (ID:60940284) | Increasing Employment Earnings: Extended Transition Services that Make a Difference | -Medium and high quality primary study |
| Jensen (2012) (ID:44048962) | Do labor market opportunities affect young women's work and family decisions? Experimental evidence from India | -Medium and high quality primary study |
| Juliet (2016) (ID:47256921) | Beyond technical skills training: the impact of credit counselling on entrepreneurial behavior of Ugandan youth | -Medium and high quality primary study |
| Kim (2019) (ID:47256972) | Estimating the effects of independent living services on educational attainment and employment of foster care youth | -Medium and high quality primary study |
| Knight (2002) (ID:44048963) | Evaluation of the Australian Wage Subsidy Special Youth Employment and Training Program, SYETP | -Medium and high quality primary study |
| Latimer, (2008) (ID:45199379) | Individual placement and support programme increases rates of obtaining employment in people with severe mental illness. | -Medium and high quality primary study |
| Lechner, (2000) (ID:45552828) | An evaluation of public-sector-sponsored continuous vocational training programs in East Germany. | -Medium and high quality primary study |
| Lee, (2005) (ID:45078802) | Training, wages, and sample selection: Estimating sharp bounds on treatment effects | -Medium and high quality primary study |
| Leos-Urbel (2014) (ID:45934206) | What Is a Summer Job Worth? The Impact of Summer Youth Employment on Academic Outcomes | -Medium and high quality primary study |
| Loyalka, (2015) (ID:45400527) | The impact of vocational schooling on human capital development in developing countries: Evidence from China. | -Medium and high quality primary study |
| Macours, (2013) (ID:45405612) | Demand versus returns? pro-poor targeting of business grants and vocational skills training | -Medium and high quality primary study |
| Malmberg-Heimonen, (2005) (ID:45552830) | Activation or discouragement-the effect of enforced participation on the success of job-search training Aktivointi vai lannistaminen-Työnhakuryhmään velvoittamisen vaikutukset. | -Medium and high quality primary study |
| McKenzie, (2017) (ID:45405613) | Identifying and Spurring High-Growth Entrepreneurship: experimental evidence from a business plan competition | -Medium and high quality primary study |
| McKenzie, (2017) (ID:45405635) | Growing Markets through Business Training for Female Entrepreneurs | -Medium and high quality primary study |
| Minnis (2014) (ID:45956781) | Yo Puedo - a conditional cash transfer and life skills intervention to promote adolescent sexual health: results of a randomized feasibility study in San Francisco | -Medium and high quality primary study |
| Morton (2011) (ID:50158885) | Empowerment-based non-formal education for Arab youth: A pilot randomized trial | -Medium and high quality primary study |
| Mulas (2018) (ID:47984557) | Coding bootcamps for youth employment: evidence from Colombia, Lebanon, and Kenya | -Medium and high quality primary study |
| Ñopo, (2007) (ID:44463341) | Occupational training to reduce gender segregation: the impacts of ProJoven | -Medium and high quality primary study |
| Nordlund, (2011) (ID:45552832) | What works best when? The role of active labour market policy programmes in different business cycles | -Medium and high quality primary study |
| Premand (2016) (ID:47984622) | Entrepreneurship education and entry into self-employment among university graduates | -Medium and high quality primary study |
| Raihan (2016) (ID:50332025) | Evaluation of a Targeted Private Sector Skill Training Programme in Bangladesh | -Medium and high quality primary study |
| Ranchhod (2016) (ID:47944300) | Estimating the Short Run Effects of South Africa's Employment Tax Incentive on Youth Employment Probabilities using A Difference-in-Differences Approach | -Medium and high quality primary study |
| Rauch (2015) (ID:47985568) | Putting entrepreneurship education where the intention to act lies: An investigation into the impact of entrepreneurship education on entrepreneurial behavior | -Medium and high quality primary study |
| Richmond (2018) (ID:47971880) | Impact evaluation of the Philippine Special Program for Employment of Students | -Medium and high quality primary study |
| Rosas (2017) (ID:45935078) | They got mad skills: the effects of training on youth employability and resilience to the Ebola shock | -Medium and high quality primary study |
| Rotheram-Borus, (2011) (ID:45402217) | Vocational training with HIV prevention for Ugandan youth. AIDS and Behavior | -Medium and high quality primary study |
| Schaeffer (2014) (ID:47955098) | RCT of a promising vocational/employment program for high-risk juvenile offenders. | -Medium and high quality primary study |
| Siaplay (2012) (ID:45935249) | The Impact of Social Cash Transfers on Young Adults' Labor Force Participation, Schooling, and Sexual Behaviors in South Africa | -Medium and high quality primary study |
| Smith (2015) (ID:47955117) | Brief report: vocational outcomes for young adults with autism spectrum disorders at six months after virtual reality job interview training. | -Medium and high quality primary study |
| Valdivia, (2014) (ID:45357848) | Business training plus for female entrepreneurship? Short and medium-term experimental evidence from Peru. | -Medium and high quality primary study |
| Vuori, (2005) (ID:45460732) | The benefits of a preventive job search program on re-employment and mental health at 2-year follow-up | -Medium and high quality primary study |
| Wehman (2013) (ID:50070505) | Competitive employment for youth with autism spectrum disorders: Early results from a randomized clinical trial. | -Medium and high quality primary study |
| Wehman, (2017) (ID:50070499) | Effects of an employer-based intervention on employment outcomes for youth with significant support needs due to autism. | -Medium and high quality primary study |
| Zamora (2018) (ID:50332089) | Are job search assistance programmes always zero-sum games? | -Medium and high quality primary study |
| Catalano (2019) (ID:45357780) | Positive youth development programs in low-and middle-income countries: a conceptual framework and systematic review of efficacy | -Low quality systematic review |
| Cobb (2009) (ID:45913829) | Transition Planning/Coordinating Interventions for Youth with Disabilities: A Systematic Review | -Low quality systematic review |
| Grimm (2015) (ID:45357776) | Do interventions targeted at micro-entrepreneurs and small and medium-sized firms create jobs? A systematic review of the evidence for low and middle income countries | -Low quality systematic review |
| Hanif, (2017) (ID:50070497) | A systematic review of vocational interventions for youth with physical disabilities. factors in studying employment for persons with disability: how the picture can change. | -Low quality systematic review |
| Jennings (2014) (ID:45956199) | Do men need empowering too? A systematic review of entrepreneurial education and microenterprise development on health disparities among inner-city black male youth | -Low quality systematic review |
| Ke, (2018) (ID:44031025) | Social Skill Interventions for Youth and Adults With Autism Spectrum Disorder: A Systematic Review | -Low quality systematic review |
| Forslund, (2004) (ID:45540757) | Employment subsidies-A fast lane from unemployment to work? | -Low quality primary study |
| Kruss G; (2012) (ID:60940283) | Developing skills and capabilities through the learnership and apprenticeship pathway systems synthesis report: assessing the impact of learnerships and apprenticeships under NSDSII | -Low quality primary study |
| Abekah-Nkrumah (2019) (ID:45883749) | Duration of High School Education and Youth Labour Market Outcomes: Evidence from a Policy Experiment in Ghana | -Low quality primary study |
| Adda, (2007) (ID:45503206) | Labour market programmes and labour market outcomes: a study of the Swedish active labour market interventions | -Low quality primary study |
| Addison, (2008) (ID:45357839) | How do different entitlements to unemployment benefits affect the transitions from unemployment into employment? | -Low quality primary study |
| Adoho (2014) (ID:44048944) | The impact of an adolescent girls employment program: the EPAG project in Liberia | -Low quality primary study |
| Aedo (2004) (ID:44463945) | The impact of training policies in Latin American and the Caribbean: the case of Programa Joven. | -Low quality primary study |
| Aflagah (2020) (ID:50331750) | Failed Promises of a Wage Subsidy: Youth and South Africa's Employment Tax Incentive | -Low quality primary study |
| Ahmad (2011) (ID:47256166) | Minimum Wage and Youth Employment The Case Study of Iran's Manufacturing Industries | -Low quality primary study |
| Alameda (2016) (ID:44031816) | Measuring the effectiveness of two public policies on the sectors of labor markets and urban infrastructure: experimental and quasi-experimental evidence from developing countries | -Low quality primary study |
| Alaref (2019) (ID:47984626) | The Short-Term Impact of Inter-Community Volunteering Activities and Soft Skills Training on Self-Reported Social Cohesion Values: Quasi-Experimental Evidence from Lebanon | -Low quality primary study |
| Alcid (2014) (ID:44473801) | A randomised controlled trial of Akazi Kanoze youth in rural Rwanda. United States Agency for International Development. | -Low quality primary study |
| Alegre (2015) (ID:50331757) | The impact of training-intensive labour market policies on labour and educational prospects of NEETs: evidence from Catalonia (Spain) | -Low quality primary study |
| Alexander (2016) (ID:47256184) | The Effects of Youth Employment: Evidence from New York City Lotteries | -Low quality primary study |
| Alzuá, (2006) (ID:44460755) | The impact of training policies in Argentina: an evaluation of Proyecto Joven | -Low quality primary study |
| Alzúa, (2016) (ID:45402349) | Long-run effects of youth training programs: Experimental evidence from Argentina. | -Low quality primary study |
| Amy (2015) (ID:47256209) | Making Summer Matter: The Impact of Youth Employment on Academic Performance | -Low quality primary study |
| Andrén, (2004) (ID:45552817) | Income effects from labor market training programs in Sweden during the 1980s and 1990s. | -Low quality primary study |
| Ardington (2016) (ID:45932005) | Social Protection and Labor Market Outcomes of Youth in South Africa | -Low quality primary study |
| Aslam, (2013) (ID:45357850) | Preparing women of substance? Education, training, and labor market outcomes for women in Pakistan. | -Low quality primary study |
| Attanasio (2017) (ID:45932051) | Vocational Training for Disadvantaged Youth in Colombia: A Long-Term Follow-Up | -Low quality primary study |
| Attanasio (2017) (ID:50331783) | Vocational Training for Disadvantaged Youth in Colombia: An Assessment of Its Long Term Effects on Crime | -Low quality primary study |
| Attanasio, (2015) (ID:45402350) | Long Term Impacts of Vouchers for Vocational Training: Experimental Evidence for Colombia | -Low quality primary study |
| Bailey (2016) (ID:45932109) | Employer-Provided Health Insurance and Job Mobility: Did the Affordable Care Act Reduce Job Lock? | -Low quality primary study |
| Bampasidou (2014) (ID:44048934) | The role of degree attainment in the differential impact of Job Corps on adolescents and young adults | -Low quality primary study |
| Bandiera (2013) (ID:50113144) | Empowering adolescent girls: Evidence from a randomised control trial in Uganda | -Low quality primary study |
| Banerjee (2018) (ID:50331794) | How important are matching frictions in the labor market? Experimental & non-experimental evidence from one Indian firm | -Low quality primary study |
| Baranowska-Rataj (2015) (ID:47256237) | The impact of the minimum wage on job separations and working hours among young people in Poland | -Low quality primary study |
| Bargain (2017) (ID:44031814) | The Effect of Social Benefits on Youth Employment Combining Regression Discontinuity and a Behavioral Model | -Low quality primary study |
| Barría, (2016) (ID:45077290) | The impact of SENAI's vocational training program on employment, wages, and mobility in Brazil: What lessons for Sub Saharan Africa? International Research Initiative on Brazil and Africa (IRIBA). | -Low quality primary study |
| Battistib (2017) (ID:50331801) | The Labour Market Integration of Refugees in Germany: Evidence from a Field Experiment | -Low quality primary study |
| Baumgartner, (2008) (ID:45458520) | Turning unemployment into selfemployment: Effectiveness of two start-up programmes | -Low quality primary study |
| Bausch (2017) (ID:47969374) | The impact of skills training on the financial behaviour, employability and educational choices of rural young people | -Low quality primary study |
| Bazen (2009) (ID:47257549) | The Differential Impact Of Federal And State Minimum Wages On Teenage Employment | -Low quality primary study |
| Beatton (2018) (ID:45932210) | Larrikin Youth: Crime and Queensland's Earning or Learning Reform | -Low quality primary study |
| Bensnes (2019) (ID:45888112) | Earning or Learning? How Extending Closing Time in the Retail Sector Affects Youth Employment and Education | -Low quality primary study |
| Berge, (2012) (ID:45402375) | Business Training in Tanzania: From Research-driven Experiment to Local Implementation | -Low quality primary study |
| Bergemann, (2009) (ID:45552818) | Evaluating the dynamic employment effects of training programs in East Germany using conditional difference-in-differences | -Low quality primary study |
| Bernhardt, (2017) (ID:45402376) | Household Matters: Revisiting the Returns to Capital among Female Micro-entrepreneurs | -Low quality primary study |
| Betcherman, (2010) (ID:45402398) | Do Employment Subsidies Work? Evidence from Regionally Targeted Subsidies in Turkey | -Low quality primary study |
| Betts (2003) (ID:45932304) | The Impact of Grading Standards on Student Achievement, Educational Attainment, and Entry-Level Earnings | -Low quality primary study |
| Betz (2010) (ID:44039987) | Testing the transition preparation training program: a randomized controlled trial | -Low quality primary study |
| Bhorat (2016) (ID:45932317) | Minimum Wages and Youth: The Case of South Africa | -Low quality primary study |
| Bhorat, (2013) (ID:45402399) | The impact of sectoral minimum wage laws on employment, wages, and hours of work in South Africa | -Low quality primary study |
| Bier (2019) (ID:50331806) | Addressing the youth skills gap through university curricula: Evidence from a quasi-experimental evaluation in Rwanda | -Low quality primary study |
| Bishop (2005) (ID:45932344) | Raising Academic Standards and Vocational Concentrators: Are They Better Off or Worse Off? | -Low quality primary study |
| Blanco (2019) (ID:50331807) | Does Youth Training Lead to Better Job Quality? Evidence from Job Corps | -Low quality primary study |
| Blasco, (2011) (ID:45460721) | The impact of active labour market policy on post-unemployment outcomes: evidence from a social experiment in Denmark | -Low quality primary study |
| Blattman (2013) (ID:44048936) | Building Women's Economic and Social Empowerment through Enterprise: An Experimental Assessment of the Women's Income Generating Support Program in Uganda | -Low quality primary study |
| Blattman (2018) (ID:50581308) | The long term impacts of grants on poverty: 9-year evidence from Uganda's Youth Opportunities Program | -Low quality primary study |
| Blattman, (2011) (ID:50158936) | Reintegrating and employment high risk youth in Liberia: Lessons from a randomized evaluation of a Landmine Action an agricultural training program for ex-combatants. | -Low quality primary study |
| Blazquez (2011) (ID:45932372) | Minimum Wage and Youth Employment Rates, 2000-2008 | -Low quality primary study |
| Blundell (2004) (ID:44048938) | Evaluating the employment impact of a mandatory job search program | -Low quality primary study |
| Bonnal (2002) (ID:45932404) | School-to-Work Transition: Apprenticeship versus Vocational School in France | -Low quality primary study |
| Boockmann (2016) (ID:45932411) | Mentoring Disadvantaged Youths during School-to-work Transition: Evidence from Germany | -Low quality primary study |
| Borland (2013) (ID:50136488) | Does coordination of welfare services delivery make a difference for extremely disadvantaged jobseekers? Evidence from the 'YP4'trial. | -Low quality primary study |
| Bozick (2017) (ID:45932460) | Do High School STEM Courses Prepare Non-college Bound Youth for Jobs in the STEM Economy? | -Low quality primary study |
| Bratberg (2000) (ID:45932469) | Transitions from school to work and the early labour market experience | -Low quality primary study |
| Bratti (2018) (ID:45861802) | Vocational Training for Unemployed Youth in Latvia: Evidence from a Regression Discontinuity Design | -Low quality primary study |
| Bravo (2012) (ID:50331818) | Effects of large-scale youth employment subsidies: Evidence from a regression discontinuity design | -Low quality primary study |
| Brooke (2016) (ID:47256349) | Fuelling financial literacy: estimating the impact of youth entrepreneurship training in Tanzania | -Low quality primary study |
| Bruhn, (2016) (ID:45402402) | Can Wage Subsidies Boost Employment in the Wake of an Economic Crisis? Evidence from Mexico | -Low quality primary study |
| Brunetti (2014) (ID:47256355) | Workplace Training Programs: Instruments for Human Capital Improvements or Screening Devices? | -Low quality primary study |
| Burke-Miller (2012) (ID:44031231) | Supported employment outcomes for transition age youth and young adults. | -Low quality primary study |
| Calero (2017) (ID:44048941) | Can arts-based interventions enhance labor market outcomes among youth? Evidence from a randomized trial in Rio de Janeiro | -Low quality primary study |
| Caliendo (2011) (ID:45357765) | Fighting youth unemployment: The effects of active labor market policies | -Low quality primary study |
| Caliendo, (2008) (ID:45465136) | The employment effects of job creation schemes in Germany-a microeconometric evaluation. | -Low quality primary study |
| Caliendo, (2015) (ID:45460702) | Getting back into the labor market: the effects | -Low quality primary study |
| Cappellini (2019) (ID:45932589) | Are Traineeships Stepping-Stones for Youth Working Careers in Italy? | -Low quality primary study |
| Carling (2005) (ID:44048991) | Does early intervention help the unemployed youth? | -Low quality primary study |
| Carter (2009) (ID:47942590) | Evaluation of a Multicomponent Intervention Package to Increase Summer Work Experiences for Transition-Age Youth With Severe Disabilities | -Low quality primary study |
| Cavaco, (2005) (ID:45540756) | Estimating the effect of a retraining program for displaced workers on their transition to permanent jobs. | -Low quality primary study |
| Centeno (2009) (ID:44048992) | Evaluating job-search programs for old and young individuals: Heterogeneous impact on unemployment duration | -Low quality primary study |
| Chakravarty (2017) (ID:47257513) | Vocational Training Programs and Youth Labor Market Outcomes: Evidence from Nepal | -Low quality primary study |
| Charlene (2008) (ID:47256394) | Effects of Minimum Wages on Youth Employment: the Importance of Accounting for Spatial Correlation | -Low quality primary study |
| Chen (2009) (ID:47984537) | Vocational Schooling, Labor Market Outcomes, and College Entry: Vocational Schooling, Labor Market Outcomes, and College Entry | -Low quality primary study |
| Cho (2015) (ID:47256408) | Differences in the effects of vocational training on men and women : constraints on women and drop-out behaviour | -Low quality primary study |
| Chong, (2006) (ID:44460787) | Does the Quality of Training Programs Matter? Evidence from Bidding Processes Data | -Low quality primary study |
| Christl (2017) (ID:45932733) | Effects of Collective Minimum Wages on Youth Employment in Austria | -Low quality primary study |
| Chun (2011) (ID:44591300) | Can Skill Diversification Improve Welfare in Rural Areas? Evidence from the Rural Skills Development Project in Bhutan | -Low quality primary study |
| Clark (2001) (ID:45932756) | The Promise of Workplace Training for Non-College Bound Youth: Theory and Evidence from German Apprenticeship | -Low quality primary study |
| Cmar (2019) (ID:47942696) | Effectiveness of a Job Search Training Program for Youth With Visual Impairments | -Low quality primary study |
| Cockx, (2003) (ID:45460705) | Vocational training of unemployed workers in Belgium | -Low quality primary study |
| Coomer (2013) (ID:45932816) | The Effect of the Minimum Wage on Covered Teenage Employment | -Low quality primary study |
| Corseuil (2014) (ID:47256377) | Youth Turnover in Brazil: Job and Worker Flows and an Evaluation of a Youth-Targeted Training Program | -Low quality primary study |
| Costa (2012) (ID:44472682) | Treatment evaluation with selective participation and ineligibles. | -Low quality primary study |
| Cover (2004) (ID:45932840) | The Effect of Changes in the Real Minimum Wage on Teenage Employment Evidence from Urban-Area Data | -Low quality primary study |
| Crépon (2013) (ID:44048983) | Do labor market policies have displacement effects? Evidence from a clustered randomized experiment | -Low quality primary study |
| Crépon, (2012) (ID:45552822) | Training the unemployed in France: how does it affect unemployment duration and recurrence? | -Low quality primary study |
| Cueto (2017) (ID:47256293) | Evaluation of the Spanish flat rate for young self-employed workers | -Low quality primary study |
| Cueto, (2009) (ID:45552823) | A nonexperimental evaluation of training programmes: regional evidence for Spain. | -Low quality primary study |
| De Giorgi (2005) (ID:45357769) | The new deal for young people five years on | -Low quality primary study |
| Decker, (2000) (ID:45460710) | Assisting unemployment insurance claimants: The long-term impacts of the job search assistance demonstration. US Department of Labor, Employment and Training Administration, Office of Workforce Security | -Low quality primary study |
| Delajara, (2006) (ID:44460789) | An evaluation of training for the unemployed in Mexico | -Low quality primary study |
| Demachi (2017) (ID:47256518) | TVET as the last educational chance : employability and family background of Ethiopian urban youth | -Low quality primary study |
| Dhushyanth (2018) (ID:45954819) | Youth Labor Skill Training in Nepal | -Low quality primary study |
| Díaz (2006) (ID:44048984) | An Evaluation of the Peruvian" Youth Labor Training Program"-PROJOVEN | -Low quality primary study |
| Diaz (2016) (ID:44031233) | Impact evaluation of the job youth training program Projoven | -Low quality primary study |
| Dmitrijeva, (2008) (ID:44463954) | Does unemployed training increase individual employability: evidence from Latvian microdata. | -Low quality primary study |
| Dmitrijeva, (2009) (ID:44460791) | The employment effects of public training programme: Evidence from Latvian micro and macro data. | -Low quality primary study |
| Dolton (2001) (ID:45933065) | The Earnings and Employment Effects of Young People's Vocational Training in Britain | -Low quality primary study |
| Dolton, (2002) (ID:45460713) | The long-run effects of unemployment monitoring and work-search programs: Some experimental evidence from the U.K department of economics | -Low quality primary study |
| Donohue (2005) (ID:47955155) | Financial management and job social skills training components in a summer business institute: a controlled evaluation in high achieving predominantly ethnic minority youth. | -Low quality primary study |
| Duncan (2010) (ID:47256560) | Are Active Labour Market Programmes Least Effective Where They Are Most Needed? The Case of the British New Deal for Young People | -Low quality primary study |
| Ebrahim (2017) (ID:47256207) | The effects of the Employment Tax Incentive on South African employment | -Low quality primary study |
| Egebark (2018) (ID:50331861) | Payroll taxes and youth labor demand | -Low quality primary study |
| Ehlert (2012) (ID:44048986) | Temporary work as an active labor market policy: Evaluating an innovative program for disadvantaged youths | -Low quality primary study |
| Eichler, (2000) (ID:45552824) | Some econometric evidence on the effectiveness of active labour market programmes in East Germany | -Low quality primary study |
| Eichler, (2002) (ID:45552825) | An evaluation of public employment programmes in the East German state of Sachsen-Anhalt | -Low quality primary study |
| Elias (2004) (ID:44048987) | An econometric cost-benefit analysis of Argentina's Youth Training Program | -Low quality primary study |
| Fallesen (2018) (ID:50331873) | The effect of active labor market policies on crime: Incapacitation and program effects | -Low quality primary study |
| Fersterer (2008) (ID:50581321) | Returns to apprenticeship training in Austria: Evidence from failed firms | -Low quality primary study |
| Fitzenberger (2001) (ID:45460715) | Employment effects of the provision of specific professional skills and techniques in Germany | -Low quality primary study |
| Fitzenberger, (2000) (ID:45552826) | Evaluating public sector sponsored training in East Germany | -Low quality primary study |
| Fitzenberger, (2007) (ID:45460714) | Long-run effects of training programs for the unemployed in East Germany | -Low quality primary study |
| Flores (2015) (ID:47979465) | Going beyond late: Bounding average treatment effects of job corps training | -Low quality primary study |
| FLORES-LAGUNES (2010) (ID:44048988) | Learning but not earning? The impact of Job Corps training on Hispanic youth | -Low quality primary study |
| French (2015) (ID:45933352) | What You Do in High School Matters: High School GPA, Educational Attainment, and Labor Market Earnings as a Young Adult | -Low quality primary study |
| Frumento (2012) (ID:45357774) | Evaluating the effect of training on wages in the presence of noncompliance, nonemployment, and missing outcome data | -Low quality primary study |
| Fukunishi (2017) (ID:47256669) | Vocational education and employment outcomes in Ethiopia: displacement effects in local labor markets | -Low quality primary study |
| Galan (2015) (ID:47943066) | Minimum Wages: Do They Really Hurt Young People? | -Low quality primary study |
| Galasso, (2004) (ID:44480162) | Assisting the transition from workfare to work: A randomized experiment. | -Low quality primary study |
| Garcia-Perez (2016) (ID:45933412) | Can Fixed-Term Contracts Put Low Skilled Youth on a Better Career Path? Evidence from Spain | -Low quality primary study |
| Gaure, (2008) (ID:45357792) | The impacts of labor market policies on job search behavior and post-unemployment job quality. | -Low quality primary study |
| Gerfin, (2002) (ID:45460717) | Does subsidised temporary employment get the unemployed back to work? Aneconometric analysis of two different schemes | -Low quality primary study |
| Gerfin, (2006) (ID:45460730) | A microeconometric evaluation of the active labour market policy in Switzerland | -Low quality primary study |
| Giuliano (2011) (ID:45933478) | Minimum Wage Effects on Employment, Substitution, and the Teenage Labor Supply: Evidence from Personnel Data | -Low quality primary study |
| Go (2012) (ID:47256715) | Money savvy youth: evaluating the effectiveness of financial education for fourth and fifth graders | -Low quality primary study |
| Göggel (2012) (ID:50581283) | Heterogeneous wage effects of apprenticeship training | -Low quality primary study |
| Grace, (2014) (ID:50136437) | Improving outcomes for unemployed and homeless young people: Findings of the YP4 clinical controlled trial of joined up case management. | -Low quality primary study |
| Graversen, (2009) (ID:45460724) | How a mandatory activation program reduces unemployment durations; the effects of distance | -Low quality primary study |
| Graversen, (2011) (ID:45460722) | An activation program as a stick to job finding | -Low quality primary study |
| Grenwelge, (2013) (ID:50070516) | The effects of the Texas Youth Leadership Forum summer training on the self-advocacy abilities of high school students with disabilities. | -Low quality primary study |
| Griffith (2001) (ID:45357775) | An approach to evaluating school-to-work initiatives: Post-secondary activities of high school graduates of work-based learning | -Low quality primary study |
| Hägglund, (2006) (ID:45465766) | Job-search assistance using the internet: experiences from a Swedish randomised experiment. | -Low quality primary study |
| Hämäläinen (2014) (ID:44048973) | The labour market impacts of a youth guarantee: lessons for Europe? | -Low quality primary study |
| Hanushek, 2017 (ID:45357720) | General education, vocational education, and labor-market outcomes over the lifecycle. | -Low quality primary study |
| Hawley, (2008) (ID:45398814) | Vocational-technical schooling and occupational matching in Thailand: Differences between men and women. | -Low quality primary study |
| Heckman (2000) (ID:44048975) | The sensitivity of experimental impact estimates (evidence from the national JTPA study) | -Low quality primary study |
| Heller (2014) (ID:44039986) | Summer jobs reduce violence among disadvantaged youth | -Low quality primary study |
| Hemmeter (2014) (ID:45933695) | Earnings and Disability Program Participation of Youth Transition Demonstration Participants after 24 Months | -Low quality primary study |
| Hicks, (2011) (ID:44461275) | Vocational education voucher delivery and labor market returns: a randomized evaluation among Kenyan youth (Report for Spanish Impact Evaluation Fund (SIEF) Phase II) | -Low quality primary study |
| Hollenbeck (2016) (ID:44048978) | Net impact and benefit-cost estimates of the workforce development system in Washington state | -Low quality primary study |
| Honorati, (2015) (ID:44480454) | The impact of private sector internship and training on urban youth in Kenya. | -Low quality primary study |
| Hora (2020) (ID:60940261) | Why targeting matters: The apprenticeship program for youth in the Czech Republic | -Low quality primary study |
| Hujer, (2006) (ID:45460966) | The effects of vocational training programmes on the duration of unemployment in Eastern Germany | -Low quality primary study |
| Hujer, (2007) (ID:45460967) | The effects of job creation schemes on the unemployment duration in Eastern Germany. Zeitschrift fur ArbeitsmarktForschung- | -Low quality primary study |
| Hujer, (2010) (ID:45464857) | How do the employment effects of job creation schemes differ with respect to the foregoing unemployment duration? | -Low quality primary study |
| Humensky (2019) (ID:45933796) | Supported Education and Employment Services for Young People with Early Psychosis in OnTrackNY | -Low quality primary study |
| Humlum (2013) (ID:45933797) | The Responses of Youth to a Cash Transfer Conditional on Schooling: A Quasi-experimental Study | -Low quality primary study |
| Hyslop (2007) (ID:47943377) | Youth minimum wage reform and the labour market in New Zealand | -Low quality primary study |
| Ibarrarán (2009) (ID:45357777) | Evaluating the impact of job training programmes in Latin America: evidence from IDB funded operations | -Low quality primary study |
| Ibarraran, (2006) (ID:44461281) | Impact evaluation of the job training component (PROCAJOVEN) of the assistance program for the building of a training and employment system in Panama (PN0125) (Ex-Post Project Evaluation Report | -Low quality primary study |
| Ibarraran, (2006) (ID:44467815) | Impact evaluation of a youth training program in the Dominican Republic: ex-post project evaluation report of the labor training and modernization project (DR0134) (Ex-Post Project Evaluation Report. | -Low quality primary study |
| Ibarraran, (2007) (ID:44467814) | Impact evaluation of a labor training program in Panama. | -Low quality primary study |
| Ibarraran, (2014) (ID:45357845) | Life skills, employability and training for disadvantaged youth: Evidence from a randomized evaluation design. | -Low quality primary study |
| Ibarraran, (2015) (ID:45404562) | Experimental Evidence on the Long-Term Impacts of a Youth Training Program | -Low quality primary study |
| Iva (2018) (ID:47256820) | Working for 200 euro? The effects of traineeship reform on labor market outcomes in Croatia | -Low quality primary study |
| Ivaschenko (2017) (ID:45933828) | Can Public Works Programs Reduce Youth Crime? Evidence from Papua New Guinea's Urban Youth Employment Project | -Low quality primary study |
| Jagannathan (2020) (ID:50331917) | A Public-Private Partnership Designed to Improve Student Soft Skills: The Johnson & Johnson Bridge-to-Employment Program | -Low quality primary study |
| Jamison (2014) (ID:45933843) | Financial Education and Access to Savings Accounts: Complements or Substitutes? Evidence from Ugandan Youth Clubs | -Low quality primary study |
| Jaramillo, (2007) (ID:44461282) | Do the poorest among the poor benefit from active labor market programs? Evidence from Peru's Projoven. | -Low quality primary study |
| Jespersen, (2008) (ID:45467208) | Costs and benefits of Danish active labour market programmes. Labour economics | -Low quality primary study |
| Jonathan (2017) (ID:47256899) | Rethinking the Benefits of Youth Employment Programs: The Heterogeneous Effects of Summer Jobs | -Low quality primary study |
| Jorge (2004) (ID:47256900) | Education, Gender and Youth in the labor market in Argentina | -Low quality primary study |
| José (2015) (ID:47256902) | Can Fixed-Term Contracts Put Low Skilled Youth on a Better Career path? Evidence from Spain | -Low quality primary study |
| Juznik (2012) (ID:45933915) | Evaluating the Effectiveness of an Institutional Training Program in Slovenia: A Comparison of Methods | -Low quality primary study |
| K?bler (2019) (ID:47943439) | Take Your Time to Grow: A Field Experiment on the Hiring of Youths | -Low quality primary study |
| Karageorgiou (2004) (ID:45933965) | The Impact of Minimum Wage on Youth and Teenage Employment in Greece | -Low quality primary study |
| Karlan, (2011) (ID:45404564) | Microcredit in Theory and Practice: Using Randomized Credit Scoring for Impact Evaluation | -Low quality primary study |
| Karymshakov (2016) (ID:45933981) | Remittances impact on youth labour supply: evidence from Kyrgyzstan | -Low quality primary study |
| Kenneth (2018) (ID:47256958) | Employment Effects of Three Rounds of Federal Minimum Wage Hikes | -Low quality primary study |
| Khan (2010) (ID:45934018) | Empowerment through ICT Education, Access and Use: A Gender Analysis of Muslim Youth in India | -Low quality primary study |
| Kluve, (2008) (ID:45552827) | Disentangling treatment effects of Active Labor Market Policies: The role of labor force status sequences | -Low quality primary study |
| Kopecna (2016) (ID:47256988) | Counterfactual Impact Evaluation of the Project Internships for Young Job Seekers | -Low quality primary study |
| Kugler (2015) (ID:47256772) | Long-Term Direct and Spillover Effects of Job Training: Experimental Evidence from Colombia | -Low quality primary study |
| Kvasnicka, (2008) (ID:45468648) | Does temporary help work provide a stepping stone to regular employment? (No. w13843). National Bureau of Economic Research. | -Low quality primary study |
| Kwauk, (2016) (ID:45405600) | Educate! Riding the Reform Wave to Scale Up Youth Entrepreneurship in Uganda (Case Study). | -Low quality primary study |
| Lalive, (2002) (ID:45468858) | The impact of active labor market programs on the duration of unemployment. Working paper/Institute for Empirical Research in Economics, 41. | -Low quality primary study |
| Lalive, (2008) (ID:45468711) | The impact of active labour market programmes on the duration of unemployment in Switzerland. | -Low quality primary study |
| Laporsek (2013) (ID:45934156) | Minimum Wage Effects on Youth Employment in the European Union | -Low quality primary study |
| Larsson (2003) (ID:45357779) | Evaluation of Swedish youth labor market programs | -Low quality primary study |
| Le Gallo (2012) (ID:50331957) | Does improving geographic mobility of young people promote their social inclusion? Evidence from a controlled experiment in France | -Low quality primary study |
| Le Gallo (2014) (ID:50331958) | Does subsidising young people to learn to drive promote social inclusion? Evidence from a large controlled experiment in France | -Low quality primary study |
| Le Gallo (2017) (ID:45934182) | Does Enhanced Mobility of Young People Improve Employment and Housing Outcomes? Evidence from a Large and Controlled Experiment in France | -Low quality primary study |
| Lee, (2005) (ID:45552829) | Analysis of job-training effects on Korean women | -Low quality primary study |
| Levinsohn (2014) (ID:44048965) | Wage subsidies and youth employment in South Africa: Evidence from a randomised control trial | -Low quality primary study |
| Lissenburgh (2004) (ID:45934272) | New Deal Option Effects on Employment Entry and Unemployment Exit: An Evaluation Using Propensity Score Matching | -Low quality primary study |
| Lleras-Muney (2020) (ID:50331963) | Do Youth Employment Programs Work? Evidence from the New Deal | -Low quality primary study |
| Loke (2016) (ID:47257046) | Boosting the power of youth paychecks: integrating financial capability into youth employment programs | -Low quality primary study |
| López-Acevedo, (2001) (ID:44467817) | An alternative technical education system in Mexico: a reassessment of CONALEP | -Low quality primary study |
| López-Acevedo, (2003) (ID:44461283) | A reassessment of technical education in Mexico. | -Low quality primary study |
| López-Acevedo, (2004) (ID:44467819) | A duration analysis of CONALEP (Mexico's national technical professional school) | -Low quality primary study |
| López-Acevedo, (2005) (ID:44467820) | Evaluation of national school for professional technology education in Mexico | -Low quality primary study |
| Lopus (2019) (ID:45934321) | Improving Financial Literacy of the Poor and Vulnerable in Indonesia: An Empirical Analysis | -Low quality primary study |
| Maibom (2014) (ID:47257079) | Can Active Labour Market Policies Combat Youth Unemployment? | -Low quality primary study |
| Maitra (2017) (ID:44048966) | Learning and earning: Evidence from a randomized evaluation in India | -Low quality primary study |
| Manuel (2011) (ID:44048942) | Effectiveness of Public Training Programs Reducing the Time Needed to Find a Job | -Low quality primary study |
| Marco (2017) (ID:47257097) | The ambiguous effects of public assistance to youth and female start-ups between job creation and entrepreneurship enhancement | -Low quality primary study |
| Maritza (2018) (ID:47257127) | Removing youth sub-minimum wage rates in Belgium: did it affect youth employment? | -Low quality primary study |
| Martins (2014) (ID:44031219) | Reemployment and substitution effects from increased activation: Evidence from times of crisis | -Low quality primary study |
| McClanahan (2004) (ID:44048968) | Enriching Summer Work: An Evaluation of the Summer Career Exploration Program. | -Low quality primary study |
| McDonald (2014) (ID:60848139) | The Long Arm of Mentoring: A Counterfactual Analysis of Natural Youth Mentoring and Employment Outcomes in Early Careers | -Low quality primary study |
| McKenzie (2016) (ID:50581294) | The demand for, and impact of, youth internships: evidence from a randomized experiment in Yemen | -Low quality primary study |
| Meager (2003) (ID:44048950) | An evaluation of business start-up support for young people | -Low quality primary study |
| Medina, (2005) (ID:44463340) | The impact of private and public job training in Colombia | -Low quality primary study |
| Meky (2015) (ID:47984547) | Labor market impacts and effectiveness of skills development programs in 5 states in India: Assam, Andhra Pradesh, Madhya Pradesh, Odisha, and Rajasthan | -Low quality primary study |
| Mensch (2004) (ID:44048969) | The effect of a livelihoods intervention in an urban slum in India: Do vocational counseling and training alter the attitudes and behavior of adolescent girls? | -Low quality primary study |
| Michael (2004) (ID:47257196) | Effective Job Search Practice in the UK's Mandatory Welfare-to-Work Programme for Youth | -Low quality primary study |
| Michaelides (2019) (ID:50331990) | Youth Unemployment and US Job Search Assistance Policy during the Great Recession | -Low quality primary study |
| Miguel (2016) (ID:47969375) | Evaluating the impact of vocational education vouchers on out-of-school youth in Kenya | -Low quality primary study |
| Millenky (2012) (ID:44048951) | Staying on course: Three-year results of the National Guard Youth ChalleNGe evaluation | -Low quality primary study |
| Modestino (2019) (ID:45934552) | How Do Summer Youth Employment Programs Improve Criminal Justice Outcomes, and for Whom? | -Low quality primary study |
| Moeletsi (2017) (ID:50331994) | South Africa's youth unemployment and the employment tax incentive: an empirical re-evaluation | -Low quality primary study |
| Mohrenweiser (2015) (ID:45934558) | Coaching Disadvantaged Young People: Evidence from Firm Level Data | -Low quality primary study |
| Morgan (2019) (ID:45954853) | The Apprenticeship-to-Work Transition: Experimental Evidence from Ghana | -Low quality primary study |
| Müller (2019) (ID:47984607) | Broken Promises: Evaluating an Incomplete Cash Transfer Program | -Low quality primary study |
| Nakasone, (2014) (ID:45357847) | Soap operas for female micro entrepreneur training. | -Low quality primary study |
| Neumark (2000)) (ID:45934674) | Evaluating School-to-Work Programs Using the New NLSY | -Low quality primary study |
| Neumark (2003) (ID:44048953) | School-to-career programs and transitions to employment and higher education | -Low quality primary study |
| Neyt (2018) (ID:47257269) | The Impact of Dual Apprenticeship Programs on Early Labour Market Outcomes: A Dynamic Approach | -Low quality primary study |
| Nivorozhkin, (2007) (ID:45552831) | Do government sponsored vocational training programmes help the unemployed find jobs? Evidence from Russia | -Low quality primary study |
| O'Connell, (2002) (ID:45552833) | Are they working? Market orientation and the effectiveness of active labour-market programmes in Ireland | -Low quality primary study |
| O'Mally (2016) (ID:60848750) | The Effect of Career Mentoring on Employment Outcomes for College Students who Are Legally Blind | -Low quality primary study |
| Osikominu, (2003) (ID:45469795) | Quick job entry or long-term human capital development? The dynamic effects of alternative training schemes. Review of Economic Studies | -Low quality primary study |
| Ouch (2017) (ID:50332009) | Vocational Training and Labour Market Transitions: A Randomized Experiment Among Cambodian Young Adults | -Low quality primary study |
| Pastore (2019) (ID:45865766) | Assessing the impact of off-and on-the-job training on employment outcomes: a counterfactual evaluation of the PIPOL program | -Low quality primary study |
| Pedersen, (2012) (ID:45469810) | Experimental evidence on the effects of early meetings and activation, IZA Discussion Paper 6970. | -Low quality primary study |
| Pereira (2003) (ID:45934869) | The Impact of Minimum Wages on Youth Employment in Portugal | -Low quality primary study |
| Perry, (2008) (ID:45460964) | Economic evaluation of the training opportunities programme in New Zealand. | -Low quality primary study |
| Pessoa (2009) (ID:45078777) | An Illustration of the Returns to Training Programmes: The Evaluation of the" Qualifying Contract" in France. | -Low quality primary study |
| Petreski (2015) (ID:45934885) | Youth Self-Employment in Households Receiving Remittances in the Republic of Macedonia | -Low quality primary study |
| Popescu (2018) (ID:47257074) | Do Apprenticeships Increase Youth Employability in Romania? A Propensity Score Matching Approach | -Low quality primary study |
| Popescu (2018) (ID:47955082) | Vocational training and employability: Evaluation evidence from Romania. | -Low quality primary study |
| Pratomo (2016) (ID:45934941) | How Does the Minimum Wage Affect Employment Statuses of Youths?: Evidence of Indonesia | -Low quality primary study |
| Radoman (2015) (ID:45888151) | Youth Training Programs and Their Impact on Career and Spell Duration of Professional Soccer Players | -Low quality primary study |
| Rankin (2014) (ID:47257409) | The success of learnerships? Lessons from South Africa.s training and education programme | -Low quality primary study |
| Reynolds, (2010) (ID:45460919) | Evaluating the impact of the winning new jobs programme on the re-employment and mental health of a mixed profile of unemployed people. International Journal of Mental Health Promotion, 12(2), 32-41. | -Low quality primary study |
| Richardson, (2002) (ID:45480021) | The effect of vocational employment training on the individual transition rate from unemployment to work | -Low quality primary study |
| Riley (2001) (ID:47257411) | Does welfare-to-work policy increase employment?: Evidence from the UK New Deal for Young People | -Low quality primary study |
| Riphahn (2015) (ID:45935047) | Apprenticeship, Vocational Training and Early Labor Market Outcomes--in East and West Germany | -Low quality primary study |
| Rodriguez-Planas (2012) (ID:44048955) | Longer-term impacts of mentoring, educational services, and learning incentives: Evidence from a randomized trial in the United States | -Low quality primary study |
| Roger (2011) (ID:44048956) | Hiring young, unskilled workers on subsidized open-ended contracts: a good integration programme? | -Low quality primary study |
| Rosas (2016) (ID:47257452) | Can you work it? Evidence on the productive potential of public works from a youth employment program in Sierra Leone | -Low quality primary study |
| Rosas-Shady, (2006) (ID:44467822) | Impact evaluation of PROJoven youth labor training program in Peru (Ex-Post Project Evaluation Report: OVE/EPPER-04/-06). | -Low quality primary study |
| Rosas-Shady, (2006) (ID:44468035) | Review of the youth worker training sub program in Colombia (CO0247) (Ex-Post Project Evaluation Report. | -Low quality primary study |
| Rosholm (2019) (ID:47955111) | Bridging the gap from welfare to education: Propensity score matching evaluation of a bridging intervention. | -Low quality primary study |
| Rotar (2012) (ID:45935091) | How Effective Is the Slovenian Institutional Training Program in Improving Youth's Chances of Reemployment? | -Low quality primary study |
| Rueda (2018) (ID:50332034) | Wage Subsidies in Labor Markets with High Informality: Evidence from Colombia's First Job Act | -Low quality primary study |
| Sacklén, (2002) (ID:45503109) | An evaluation of the Swedish trainee replacement schemes. IFAU-Institute for Labour Market Policy Evaluation | -Low quality primary study |
| Schochet (2001) (ID:50158887) | National Job Corps Study: the impacts of job corps on participants' employment and related outcomes | -Low quality primary study |
| Schochet (2008) (ID:44031221) | Does job corps work? Impact findings from the national job corps study | -Low quality primary study |
| Schochet, (2003) (ID:45078754) | National Job Corps Study: Findings using administrative earnings records data. | -Low quality primary study |
| Shahnaz, (2008) (ID:45357849) | Providing microfinance and social space to empower adolescent girls: An evaluation of BRAC's ELA centres. | -Low quality primary study |
| Shiner (2004) (ID:60848137) | Mentoring Disaffected Young People: An Evaluation of Mentoring Plus | -Low quality primary study |
| Sørensen, (2012) (ID:45460725) | Effects of intensifying labor market programs on post-unemployment wages: Evidence from a controlled experiment | -Low quality primary study |
| Speckesser, (2004) (ID:45460736) | Using social insurance data for the evaluation of active labour market policy: Employment effects of further training for the unemployed in Germany | -Low quality primary study |
| Stephan, (2006) (ID:45460735) | The effects of active labor market programs in Germany: An investigation using different definitions of non-treatment | -Low quality primary study |
| Stephan, (2008) (ID:45550699) | The relative effectiveness of selected active labour market programmes and the common support problem. | -Low quality primary study |
| Strickland (2013) (ID:47985475) | JobTIPS: A transition to employment program for individuals with autism spectrum disorders | -Low quality primary study |
| Stromback (2010) (ID:45935353) | Earnings, Schooling and Vocational Education and Training | -Low quality primary study |
| Swain, (2014) (ID:45402219) | Evaluating the impact of training in self-help groups in India. | -Low quality primary study |
| Tattara (2009) (ID:45935397) | Can Employment Subsidies and Greater Labour Market Flexibility Increase Job Opportunities for Youth? Revisiting the Italian On-the-Job Training Programme | -Low quality primary study |
| Torun (2019) (ID:45935449) | Ex-Ante Labor Market Effects of Compulsory Military Service | -Low quality primary study |
| Tuomala (2007) (ID:47257627) | Vocational Labour Market Training in Promoting Youth Employment | -Low quality primary study |
| Upward (2002) (ID:45935485) | Evaluating Outcomes from the Youth Training Scheme Using Matched Firm-Trainee Data | -Low quality primary study |
| Valiente (2020) (ID:60927739) | Evaluating dual apprenticeship effects on youth employment: A focus on the mechanisms | -Low quality primary study |
| van (2006) (ID:45460734) | Treatment effect of job-training programmes on unemployment duration in Slovakia | -Low quality primary study |
| van Ours (2006) (ID:47257642) | Duration of unemployment benefits and quality of post-unemployment jobs : evidence from a natural experiment | -Low quality primary study |
| Van Reenen (2004) (ID:44031223) | Active labor market policies and the British New Deal for the young unemployed in context | -Low quality primary study |
| Vernon (2015) (ID:47257654) | Increasing Youth Financial Capability: An Evaluation of the MyPath Savings Initiative | -Low quality primary study |
| Vimal (2015) (ID:47257665) | Estimating the Effects of South Africa's Youth Employment Tax Incentive – An Update | -Low quality primary study |
| Wagner (2016) (ID:47944854) | The Benefits of High School Career and Technical Education (CTE) for Youth With Learning Disabilities | -Low quality primary study |
| Webb (2014) (ID:44048958) | How targeted is targeted tax relief? Evidence from the unemployment insurance youth hires program | -Low quality primary study |
| Webb (2016) (ID:45935582) | Targeting Tax Relief at Youth Employment | -Low quality primary study |
| Wilkinson (2003) (ID:45077289) | New Deal for Young People: Evaluation of unemployment flows. | -Low quality primary study |
| Wolff, (2001) (ID:45357843) | The Hungarian unemployment insurance benefit system and incentives to return to work. | -Low quality primary study |
| Yuen (2003) (ID:45935687) | The Effect of Minimum Wages on Youth Employment in Canada: A Panel Study | -Low quality primary study |

Annex 8: Supplementary information, Tables and Figures

Table 16: Publication period of included studies

| **Period** | **Studies** | **%** |
| --- | --- | --- |
| 2000-2004 | 53 | 13.3 |
| 2005-2009 | 73 | 18.3 |
| 2010-2014 | 116 | 29.1 |
| 2015-2019 | 157 | 39.4 |
| Total | 399 | 100 |

Table 17: Country studied and randomised controlled trial

| Geographical region |  | RCTs | Share (%) | |
| --- | --- | --- | --- | --- |
| World Bank | Sub-Saharan Africa | 42 | 27.6 | |
|  | North America | 38 | 25.0 | |
|  | Latin America and Caribbean | 24 | 15.8 | |
|  | Europe and Central Asia | 22 | 14.5 | |
|  | East Asia and Pacific | 9 | 5.9 | |
|  | Middle East and North Africa | 9 | 5.9 | |
|  | South Asia | 8 | 5.3 | |
|  | **Sub-total** | **152** | **100** | |
| Country | USA | 38 | 25.0 | |
|  | Uganda | 16 | 10.5 | |
|  | Dominican Republic | 8 | 5.3 | |
|  | Colombia | 7 | 4.6 | |
|  | Kenya | 7 | 4.6 | |
|  | Other countries | 89 | 58.6 | |
|  | **Sub-total** | **165** | **108.6** | |
| *Note: Total percentage adds to more than 100 as a cross-country Study or RCT may be coded for more than one country.* | | | |  |

Table 18: Countries studied and systematic reviews

| Region | Studies | Share (%) |
| --- | --- | --- |
| USA | 13 | 61.9 |
| UK | 8 | 38.1 |
| India | 6 | 28.6 |
| Argentina | 5 | 23.8 |
| Chile | 5 | 23.8 |
| Colombia | 5 | 23.8 |
| Dominican Republic | 5 | 23.8 |
| Mexico | 5 | 23.8 |
| Peru | 5 | 23.8 |
| Total | **21** | **100** |
| *Note: Most Systematic Reviews were conducted in multiple countries* | | |

Table 19: Geographical Regions, World Bank classification

| **Geographical Regions, World Bank classification** | | |
| --- | --- | --- |
| **Region** | **Studies** | **(%)** |
| Sub-Saharan Africa | 65 | 19.2 |
| Latin America & Caribbean | 66 | 19.5 |
| East Asia and Pacific | 32 | 9.4 |
| South Asia | 27 | 8.0 |
| Europe and Central Asia | 152 | 44.8 |
| North America | 85 | 25.1 |
| Middle East & North Africa (MENA) | 17 | 5.0 |
| **Total** | **399** | **131.0** |
| *Note: Total percentage add to more than 100, as a region may be coded more than once. For instance, cross-country studies such as systematic reviews may be coded for more than one region.* | | |

Table 20: Top 10 countries studied

| **Region** | **Studies** | **(%)** |
| --- | --- | --- |
| USA | 85 | 21.3 |
| Germany | 31 | 7.8 |
| UK | 26 | 6.5 |
| Uganda | 20 | 5.0 |
| Peru | 18 | 4.5 |
| Colombia | 16 | 4.0 |
| India | 16 | 4.0 |
| France | 15 | 3.8 |
| South Africa | 15 | 3.8 |
| Argentina | 14 | 3.5 |
| **Total** | **256/399** | **64.2** |

Table 21: Authors with >2 publications (n=45)

| **Region** | **Authors** | **%** |
| --- | --- | --- |
| Asia | 2 | 4.4 |
| Africa | 2 | 4.4 |
| Latin America | 4 | 8.9 |
| North America | 16 | 35.6 |
| Europe | 21 | 46.7 |
| **Total** | **45** | **100** |

Table 22: Authors with >3 publications by geographical regions, (n=13)

| Author | Publications | Country | Region |
| --- | --- | --- | --- |
| Ibarraren P | 8 | USA | North America |
| Blattman C | 8 | USA | North America |
| Attanasio O | 6 | UK | Europe |
| Alzua M L | 4 | Argentina | South America |
| Groh M | 4 | USA | North America |
| Lopez A | 4 | USA | North America |
| Crepon B | 3 | France | Europe |
| Caliendo M | 3 | Germany | Europe |
| Fitzenberger B | 3 | Germany | Europe |
| Hujer R | 3 | Germany | Europe |
| Le Gallo J | 3 | France | Europe |
| Mckenzie D | 3 | USA | North America |
| Schochet P | 3 | USA | North America |
| Total | 55 | - | - |

Table 23: Distribution of studies by population sub-groups

| Population sub-group |  | Studies | Share (%) |
| --- | --- | --- | --- |
| Demographic factors | **Gender** |  |  |
|  | Female | 309 | 77.4 |
|  | Male | 326 | 81.7 |
|  | **Age (Years)** |  |  |
|  | 15-19 | 308 | 77.2 |
|  | 20-24 | 302 | 75.7 |
|  | 25-29 | 209 | 52.4 |
|  | 30-35 | 128 | 32.1 |
| Socio-economic factors | **Location status^§^** |  |  |
|  | Rural | 134 | 33.6 |
|  | Urban | 75 | 18.8 |
|  | **Social status** |  |  |
|  | Disadvantaged background* | 139 | 34.8 |
|  | Disability | 33 | 8.3 |
|  | Fragility, Conflict & Violence | 8 | 2.0 |
|  | Humanitarian settings | 6 | 1.5 |
|  | Criminal background | 3 | 0.8 |
|  | Ethnic minority | 3 | 0.8 |
| Total |  | **399** | **100%** |

**Disadvantaged background (low-income families or low education)*

*§ Substantial number of studies did not report location status*

Table 24: Evidence types by biological population factors

|  |  |  |  |  |  |  |
| --- | --- | --- | --- | --- | --- | --- |
| Studies | **Age (years)** | | | | **Gender** | |
|  | **15-19** | **20-24** | **25-29** | **30-35** | **Male** | **Female** |
| Experimental | 135 | 137 | 91 | 61 | 137 | 153 |
| Non-experimental matching | 138 | 131 | 94 | 53 | 131 | 133 |
| Other regression | 19 | 18 | 16 | 12 | 28 | 28 |
| Systematic reviews | 17 | 17 | 9 | 3 | 14 | 13 |
| Total | **309** | **303** | **210** | **129** | **310** | **327** |

Figure 13: Interventions by economic outcomes

**
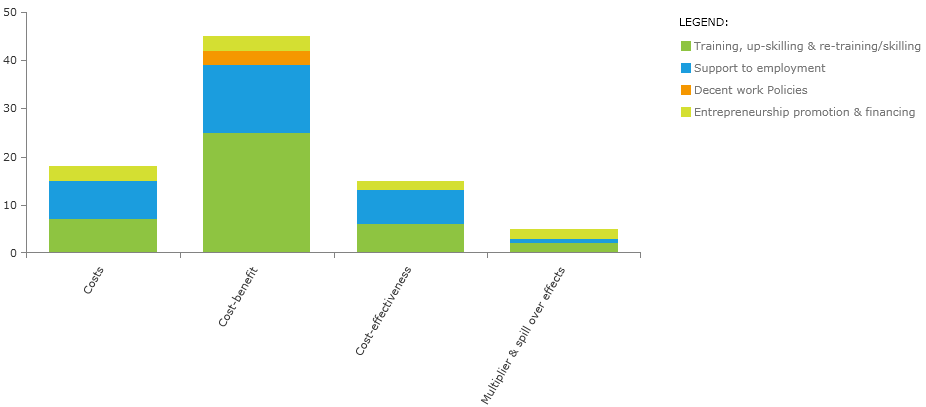
**

Figure 14: Interventions by education & skills outcomes


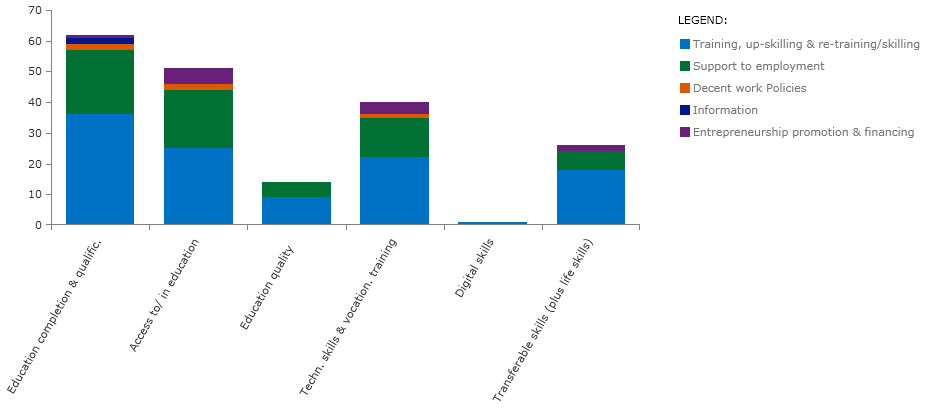


Figure 15: Interventions by entrepreneurship outcomes

**
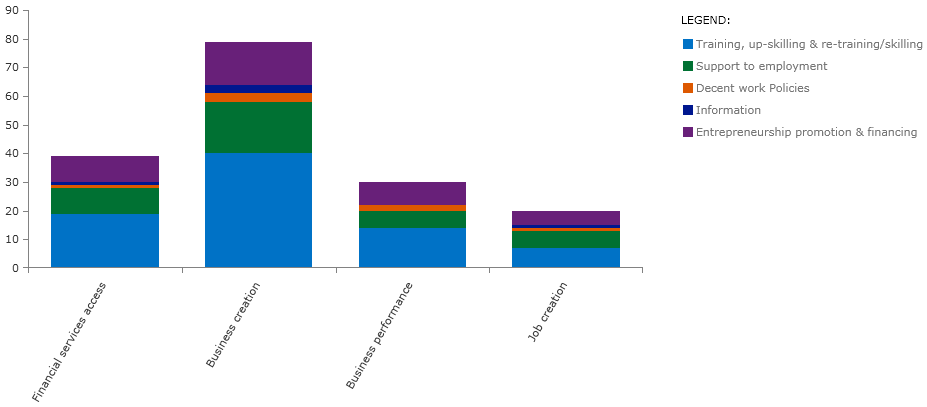
**

Figure 16: Interventions by welfare outcomes


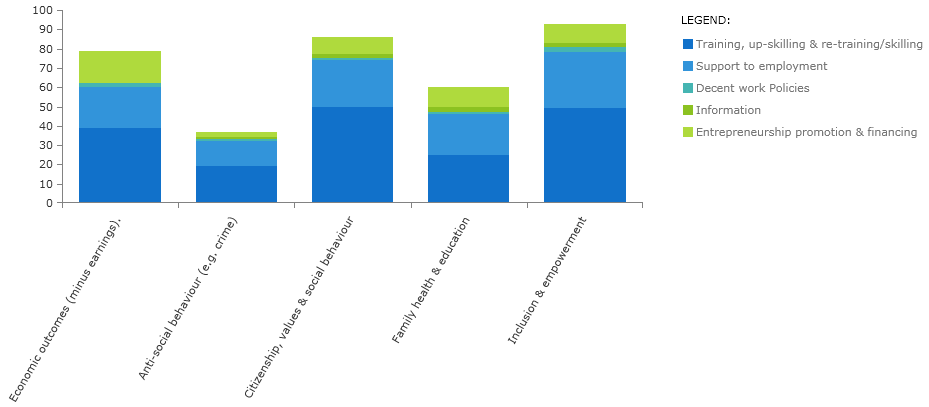


**Entrepreneurship promotion and financing**

**Entrepreneurship promotion and financing**

**Support to employment**

**Support to employment**

**Training, up-skilling and retraining/ re-skilling**

**Training, up-skilling and retraining/ re-skilling**

**Activities**

**Activities**

**Outputs**

**Outputs**

**Impact**

**Impact**

**Outcomes**

**Outcomes**

**Inputs**

**Inputs**

**Interventions**

**Interventions**

-Government policies on employment

-Education

-Partnerships with firms

- Job-marketing

-Government policies on employment

-Education

-Partnerships with firms

- Job-marketing

-Policies created

-Hard and soft skills acquired

-On job skills acquired

-Job opportunities got

-Policies created

-Hard and soft skills acquired

-On job skills acquired

-Job opportunities got

Employability of youths

Employability of youths

-Employee mentoring (OTJ)

-Wage subsidies

-Career guidance

-Employee mobility and placements

-Government policies

-Employee mentoring (OTJ)

-Wage subsidies

-Career guidance

-Employee mobility and placements

-Government policies

-Funds

-Education centres

-Marketing platforms

-Firms

-Funds

-Education centres

-Marketing platforms

-Firms

Youth Employment

Youth Employment

-Government creates MF fund centre

-Attracting donors

-Establishing business training centres

-Training of trainers

-Matching youths with mentors

-Government putting policies on loans

-Government creates MF fund centre

-Attracting donors

-Establishing business training centres

-Training of trainers

-Matching youths with mentors

-Government putting policies on loans

-MF centres created

-Grants provided

-Trained youths

-Policies created

-Life skills acquired

-MF centres created

-Grants provided

-Trained youths

-Policies created

-Life skills acquired

Entrepreneurship

Entrepreneurship

-Funds

-Trainers

-Mentors

-Donors

-Funds

-Trainers

-Mentors

-Donors

-Grants

-Loan guarantees

-Microfinance

-Business mentoring

-Crowd funding

-Micro-franchising

-Grants

-Loan guarantees

-Microfinance

-Business mentoring

-Crowd funding

-Micro-franchising

Youths getting employment skills

Youths getting employment skills

-Hard skills acquired

-soft skills acquired

-Life skills attained

-Hard skills acquired

-soft skills acquired

-Life skills attained

-Government create skills institutions

-Training of Trainers

-Curriculum review to improve employability

- Establish vocational schools

-Government create skills institutions

-Training of Trainers

-Curriculum review to improve employability

- Establish vocational schools

-Skills institutions

-Trainers

-Vocational schools

-Skills institutions

-Trainers

-Vocational schools

-TVET

Training of Trainers

-Business skills training

-Internship & Apprenticeship

-PLAR

-Life skills training

- Youth are willing to learn or participate in intervention and therefore low attrition rates are expected.
- Trainers have the needed skills and are available to teach
- Funds are available to finance interventions
- Government buy-in especially with reference to interventions implemented in partnership models
- Favorable regulatory and peaceful environment
- Presence of well-equipped training centers and firms
- Youth are willing to learn or participate in intervention and therefore low attrition rates are expected.
- Trainers have the needed skills and are available to teach
- Funds are available to finance interventions
- Government buy-in especially with reference to interventions implemented in partnership models
- Favorable regulatory and peaceful environment
- Presence of well-equipped training centers and firms

Figure 17: Basic Theory of Change for the Youth Employment Interventions

1. CAPLA = Canadian Association for Prior Learning Assessment [↑](#footnote-ref-1)
